# Visible‐Light Switchable Rings and Chains in Dynamic Covalent Imine Chemistry

**DOI:** 10.1002/chem.202501047

**Published:** 2025-04-07

**Authors:** Jona Voss, Yannic Hartmann, Esther Nieland, Andreas Mix, Bernd M. Schmidt

**Affiliations:** ^1^ Institut für Organische Chemie und Makromolekulare Chemie Heinrich‐Heine‐Universität Düsseldorf Universitätsstr. 1 Düsseldorf Germany; ^2^ Organische und Bioorganische Chemie Universität Bielefeld Universitätsstr. 25 Bielefeld Germany; ^3^ Institut für Anorganische Chemie und Strukturchemie Universität Bielefeld Universitätsstr. 25 Bielefeld Germany

**Keywords:** aldehydes, amines, macrocycles, photochemistry, supramolecular chemistry

## Abstract

The self‐assembliy of red‐light switchable, functionalised *ortho*‐difluoroazobenzene isomers *E*‐/*Z*‐**A** with aliphatic diamines exhibits an alternating match/mismatch behaviour depending on the diamine's chain length. While even‐numbered diamines exclusively form imine macrocycles with the *Z*‐configured azobenzene isomer, odd‐numbered diamines form photoswitchable, defined macrocycles. The formation of *Z*,*Z*‐**A^2^X^2^**‐type macrocycles was demonstrated with butane‐1,4‐ (**B**) and hexane‐1,6‐diamine (**H**), while additionally *E*,*E*‐**A^2^X^2^**‐type macrocycles were obtained with propane‐1,3‐ (**Pr**), pentane‐1,5‐diamine (**Pe**), and diethylene glycol bisamine (**O**), validated by single‐crystal X‐ray structures of *E*,*E*‐**A^2^Pr^2^** and *E*,*E*‐**A^2^O^2^**. The observed reactivity differences arise from the preferred conformations of the flexible diamines in solution, which alternate predictably with odd and even numbers of methylene groups, investigated by ^19^F‐DOSY NMR experiments, MALDI‐MS measurements, and UV/Vis spectroscopy. These findings provide detailed insight into photoresponsive self‐assembled systems and highlight the potential of azobenzenes in dynamic covalent chemistry, offering new opportunities for controlling structure and function in adaptive materials.

## Graphical abstract

The combination of a visible‐light switchable azobenzene **A** with aliphatic diamines **X** of variable chain lengths gives access to various macrocyclic isomers. While diamines with even numbers of methylene groups exclusively create *Z*,*Z*‐**A^2^X^2^**‐type macrocycles, both *Z*,*Z*‐ and *E*,*E*‐**A^2^X^2^**‐type macrocycles are accessible starting from odd‐numbered diamines. This study gives a deepened overview of the accessibility of macrocyclic isomers governed by a rational even–odd alteration of simple diamines.

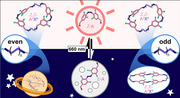

## 1 Introduction

One of the essential concepts in supramolecular chemistry is the spontaneous self‐assembly of multi‐component molecular structures. Self‐assembly has been used to access functional nanoarchitectures with a variety of shapes and sizes, largely depending on the building blocks and conditions employed.^[^ ^1^ ^]^ In addition to this, a very important approach for creating reconfigurable or multifunctional devices and nanomaterials is the ability to precisely alter the structure and function of complex supramolecular assemblies using internal or external triggers after assembly.^[^ ^2^ ^]^ Numerous responsive systems have been reported in the literature, employing a variety of stimuli such as chemical triggers,^[^ ^2a^ ^]^ temperature,^[^ ^2b^ ^]^ mechanical force,^[^ ^2c, d^ ^]^ and light.^[^ ^3^ ^]^ For any type of supramolecular structure, albeit being complex three‐dimensional assemblies, yields are often high under equilibrium conditions when employing dynamic covalent bonds.^[^ ^1^ ^]^ This is because unstable kinetic intermediates decompose, undergo error correction, and can react again. Entropically, closed architectures are preferred as long as the precursors are sufficiently preorganised.^[^ ^4^ ^]^ Being able to influence these delicate equilibria using light is of great interest. One of the most commonly used classes of photoswitches, azobenzenes, bear several advantages, such as a distinct geometry change upon isomerisation, minimal photodegradation, and, in some cases, the ability to switch using visible light.^[^ ^3^, ^5^, ^6^ ^]^ In the last years, different supramolecular systems have been investigated that undergo reversible assembly and disassembly upon excitation with light, e.g., ranging from dissipative metal–organic cages^[^ ^4^, ^7^ ^]^ to dynamic covalent organic assemblies.^[^ ^8^ ^]^ In addition, dynamic exchange of imines has also been widely used in preparing responsive dynamic covalent polymers,^[^ ^1d^ ^]^ and the reversible covalent imine bonds have also been applied to facilitate the recyclability of polymers,^[^ ^9^ ^]^ as this class of polymers combines intrinsic reversibility with the robustness of covalent bonds.^[^ ^1a,d^ ^]^ Intriguingly, the formation of oligomeric or polymeric species is not desirable when aiming for the assembly of discrete supramolecular species, and vice versa. However, there are fascinating studies dealing with the chemistry at this interface, especially by the group of Gottfried focusing on on‐surface polymerisation phenomena,^[^ ^10^ ^]^ in addition to the works of Moore on shape‐persistent arylene ethynylene macrocycles, competing with the formation of oligomeric species.^[^ ^4^, ^11^ ^]^ The group of Dichtel reported the formation of covalent organic framework (COF)‐like materials from macrocyclic precursors that convert to aggregated macrocycles, whose imines undergo exchange much more slowly via the formation of intermediate oligomers.^[^ ^12^ ^]^

## 2 Results and Discussion

**Scheme 1:**
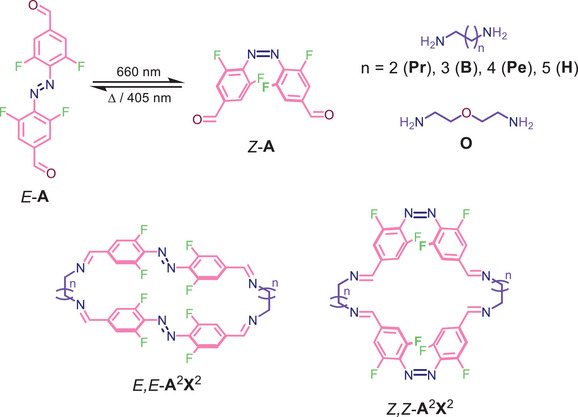
Structures of the employed building blocks azobenzenes *E*‐/*Z*‐**A** and diamines **Pr**, **B**, **Pe**, **H**, **O**, and macrocycles *E*,*E*‐ and *Z*,*Z*‐**A** ^**2**^ **X** ^**2**^.

We present herein the formation of a dissipative dynamic covalent system using red light as a stimulus, allowing for discrimination between acyclic imine oligomers and macrocycles based on both *E*‐ and *Z*‐**A**. The *ortho*‐fluorinated, red‐light switchable 4,4′‐(diazene‐1,2‐diyl)bis(3,5‐difluorobenzaldehyde) (**A**),^[^ ^8^, ^13^ ^]^ which was first synthesised by the group of Pianowski,^[^ ^14^ ^]^ was used as a ditopic aldehyde and combined with *α*,*ω*‐diamines of variable chain lengths (Scheme 1).

**Figure 1:**
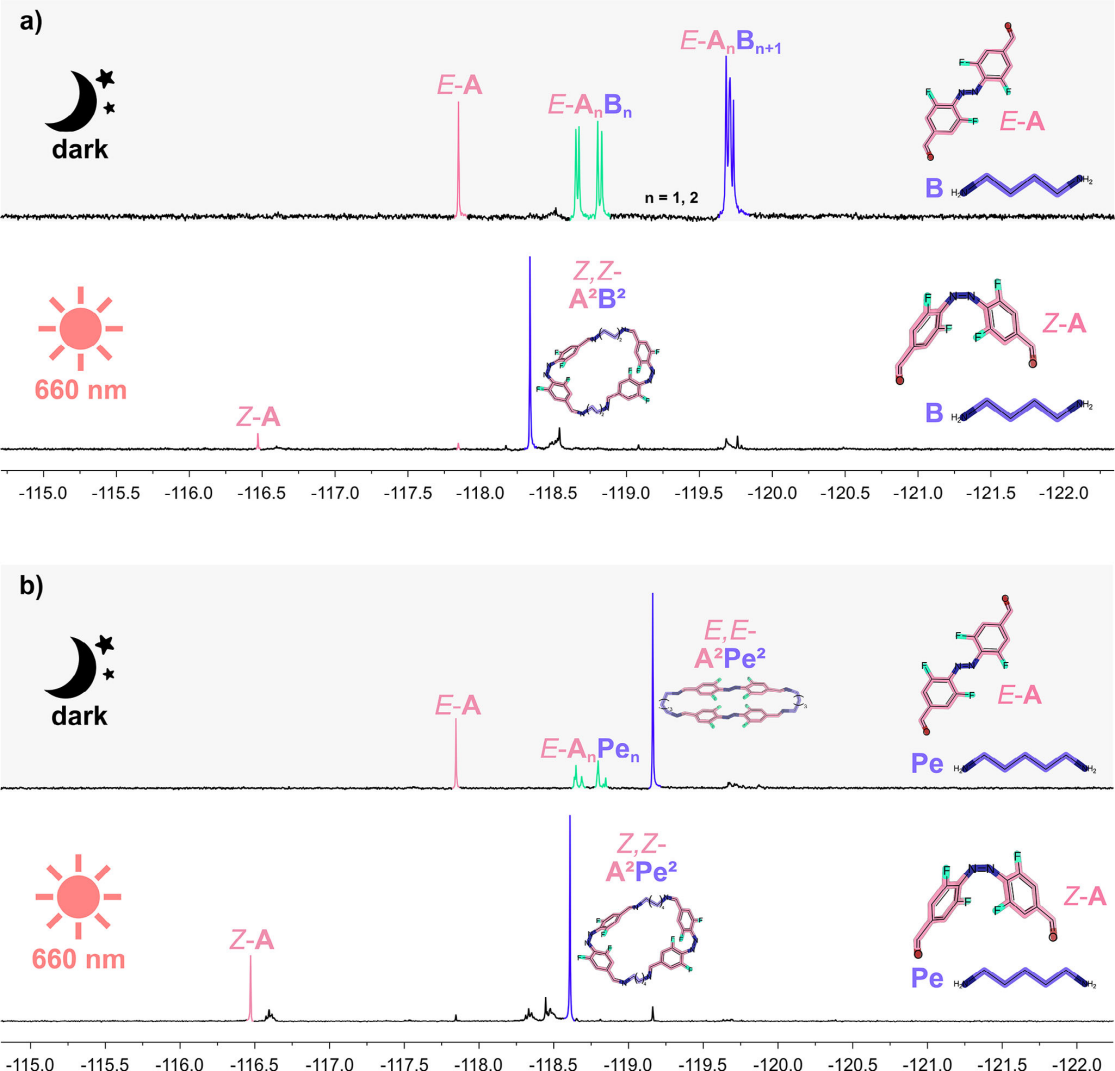
^19^F{^1^H} NMR spectra (5.0 mM, CDCl_3_, 282 MHz). (a) Combination of azobenzene **A** and diamine **B**. Top: reaction of *E*‐**A** and diamine **B** yielding different acyclic species in the dark after 3 days; bottom: ***Z*,*Z*‐A^2^B^2^** macrocycle assembly under continuous irradiation with red light (660 nm) for further 3 days. (b) Combination of **A** and diamine **Pe**. Top: reaction of *E*‐**A** and diamine **Pe** yielding the *E*,*E*‐**A^2^Pe^2^** macrocycle in the dark after 3 days; bottom: *Z*,*Z*‐**A^2^Pe^2^** macrocycle assembly under continuous irradiation with red light (660 nm) for further 3 days. The molecular geometries were obtained from UFF (Universal Force Field) force field calculations embedded in Avogadro.^[^ ^17^ ^]^

Previously, we reported the reaction between **A** and the simple ethylene‐1,2‐diamine (**E**),^[^ ^8d^ ^]^ since halogen‐bonding motifs are binding too weakly in solution to study photoswitching.^[^ ^15^ ^]^ While linear *E*‐**A** formed insoluble oligomers with **E**, the employment of pincer‐like *Z*‐**A** led to the preferential formation of macrocyclic *Z*,*Z*‐**A** ^**2**^ **E** ^**2**^.^[^ ^16^ ^]^ In contrast, more rigid 1,2‐diaminocyclohexane (**E**) formed macrocycles of various shapes with both *E*‐ and *Z*‐**A**. Inspired by these findings, we hypothesized that the structure of entities formed between A and flexible, thus non‐precoordinated, *α*,*ω*‐diamines would be mostly affected by the shape of the azobenzene's isomers. Such assemblies might be switched between ring and chain entities using visible light. Expecting a similar behavior to the combination of E with *E*‐**A**, we first combined azobenzene *E*‐**A** with butane‐1,4‐diamine (**B**) in CDCl_3_ at 5.0 mM (Figure 1a).

**Figure 2:**
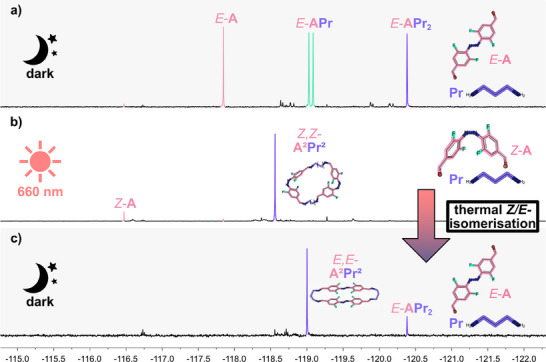
^19^F{^1^H} NMR spectra (5.0 mM, CDCl_3_, 282 MHz) of the reaction of *E*‐**A** and diamine **Pr**. Top: Formation of different acyclic imine oligomers in the dark after 3 days. Middle: *Z*,*Z*‐**A^2^Pr^2^** macrocycle assembly under continuous irradiation with red light (660 nm) for further 3 days. Bottom: Thermal *Z*/*E*‐isomerisation of in situ‐generated *Z*,*Z*‐**A^2^Pr^2^** to obtain the *E*,*E*‐**A^2^Pr^2^** macrocycle. The molecular geometries were obtained from UFF (Universal Force Field) force field calculations embedded in Avogadro.^[^ ^17^ ^]^

However, contrary to previous findings when employing *E* **‐A**, we did not observe the formation of insoluble imine oligomers^[^ ^8d^ ^]^ after three days in the dark, but distinct acyclic species. Although we employed 1.2 equivalents of the diamine, we observed unreacted *E*‐**A** alongside a monofunctionalised aldehyde **A** *_**n**_* **B** *_**n**_* (likely **AB** and **A**_**2**_ **B**_**2**_ due to mass spectrometric data and their well‐defined ^19^F{^1^H} NMR spectroscopic signals (see Figures S10–S12) and bisimine species **AB**_**2**_ and tetraamine **A**_**2**_ **B**_**3**_. The species were detected in a molar ratio of 8:40:42 (*E*‐**A**:**A** *_**n**_* **B** *_**n**_*:**A** *_**n**_* **B** *_**n**_*_**+** **1**_, integration from ^19^F{^1^H} NMR spectrum, Supporting Information chapter I). Analyses by ^19^F diffusion‐ordered (DOSY) NMR spectroscopy revealed the expected larger sizes of **AB** (solvodynamic radius *r*_s_ between 6.60 and 6.86 Å) and **A** *_**n**_* **B** *_**n**_*_ **+** **1**_ with *r*_s_ = 8.16 Å compared to *E*‐**A** with *r*_s_ = 3.68 Å (Figure S53 and Table S6). Irradiation of this ternary mixture with red light (660 nm) for further three days led to the preferred formation of a single species with a distinct singlet at −118.32 ppm in the ^19^F{^1^H} NMR spectrum. A matrix‐assisted laser desorption/ionisation (MALDI)‐mass spectrum revealed it as *Z*,*Z*‐**A** ^**2**^ **B** ^**2**^ with *r*_s_ = 6.11 Å. The formation of *Z*,*Z*‐**A** ^**2**^ **B** ^**2**^ was also achieved when adding **B** to a *Z*‐**A**‐enriched solution (by prior irradiation with red light). The selective formation of *Z*,*Z*‐**A** ^**2**^ **B** ^**2**^ starting from this *E*‐**A**‐based mixture under irradiation indicates that intermediates containing both *E*‐ and *Z*‐**A** (e.g., bisimines or macrocycles) are only transient kinetic species that readily undergo derivatisation to *Z*,*Z*‐**A** ^**2**^ **B** ^**2**^. Indeed, all other discussed systems herein with different diamines behave similarly (see below), contrasting the previously reported system between **A** and **D**.^[^ ^8d^ ^]^ A nearly identical behaviour, in the dark and under irradiation, was observed when **A** was combined with the longer diamine hexane‐1,6‐diamine (**H**) (Figures S37–S39). A ternary mixture containing *E*‐**A**, **A** *_**n**_* **H** *_**n**_*, and **A** *_**n**_* **H** *_**n**_*_ **+** **1**_ (molar ratio of 7:35:58) was converted into *Z*,*Z*‐**A** ^**2**^ **H** ^**2**^ (resonating at −118.4 to −118.6 ppm in the ^19^F{^1^H} NMR spectrum with *r*_s_ between 6.45 and 8.61 Å) under irradiation with red light as observed by ^1^H‐, ^19^F{^1^H} NMR spectroscopy and MALDI‐mass spectrometry (MS) and again reproduced by adding **H** to a *Z*‐**A**‐enriched solution. The presence of an additional smaller signal at −118.52 ppm, which is formed in both cases, could be due to the increased flexibility of the macrocycle, allowing it to adapt to different conformations in solution, or it originates from another macrocycle that varies in size. Attempts were made to isolate the generated imine macrocycles after reduction to the corresponding amine macrocycles with NaBH_4_;^[^ ^8d^ ^]^ however, all efforts failed due to the inherent and substantial reduction of the diazenyl units to hydrazines, leading to inseparable product mixtures. Diamines **E**,^[^ ^8d^ ^]^ **B**, and **H** with an even number of carbon atoms all formed acyclic species with *E*‐**A** in the dark, with a tendency towards **A** *_**n**_* **X** *_**n**_* and **A** *_**n**_* **X** *_**n**_*_ **+** **1**_ species (**X** = **B**, **H**) of distinct size, rather than polymeric A*_n_* E*_n_*. Employing *Z*‐**A** leads to the preferred formation of *Z*,*Z*‐**A** ^**2**^ **X** ^**2**^ macrocycles (**X** = **E**, **B**, **H**). In fact, similar reactivities of aliphatic diamines **E**, **B**, and **H** with an even number of carbon atoms for cage‐forming reactions with tritopic aldehydes were reported by Cooper et al.^[^ ^18^ ^]^ Analogously to Cooper's observations, we expected an inherently different reactivity when employing propane‐1,3‐diamine (**Pr**) and pentane‐1,5‐diamine (**Pe**) with an odd number of carbon atoms. Following the reaction between *E*‐**A** and **Pr** in the dark by ^19^F{^1^H} NMR spectroscopy (Figure 2), we observed a plethora of different species (partly precipitating out of solution), some of which again were identified as *E*‐**A**, monofunctionalised **A** *_**n**_* **Pr** *_**n**_* (*r*_s_ between 4.29 and 4.35 Å), and bisimine **A** *_**n**_* **Pr** *_**n**_*_ **+** **1**_ (*r*_s_ = 5.24 Å).

**Figure 3:**
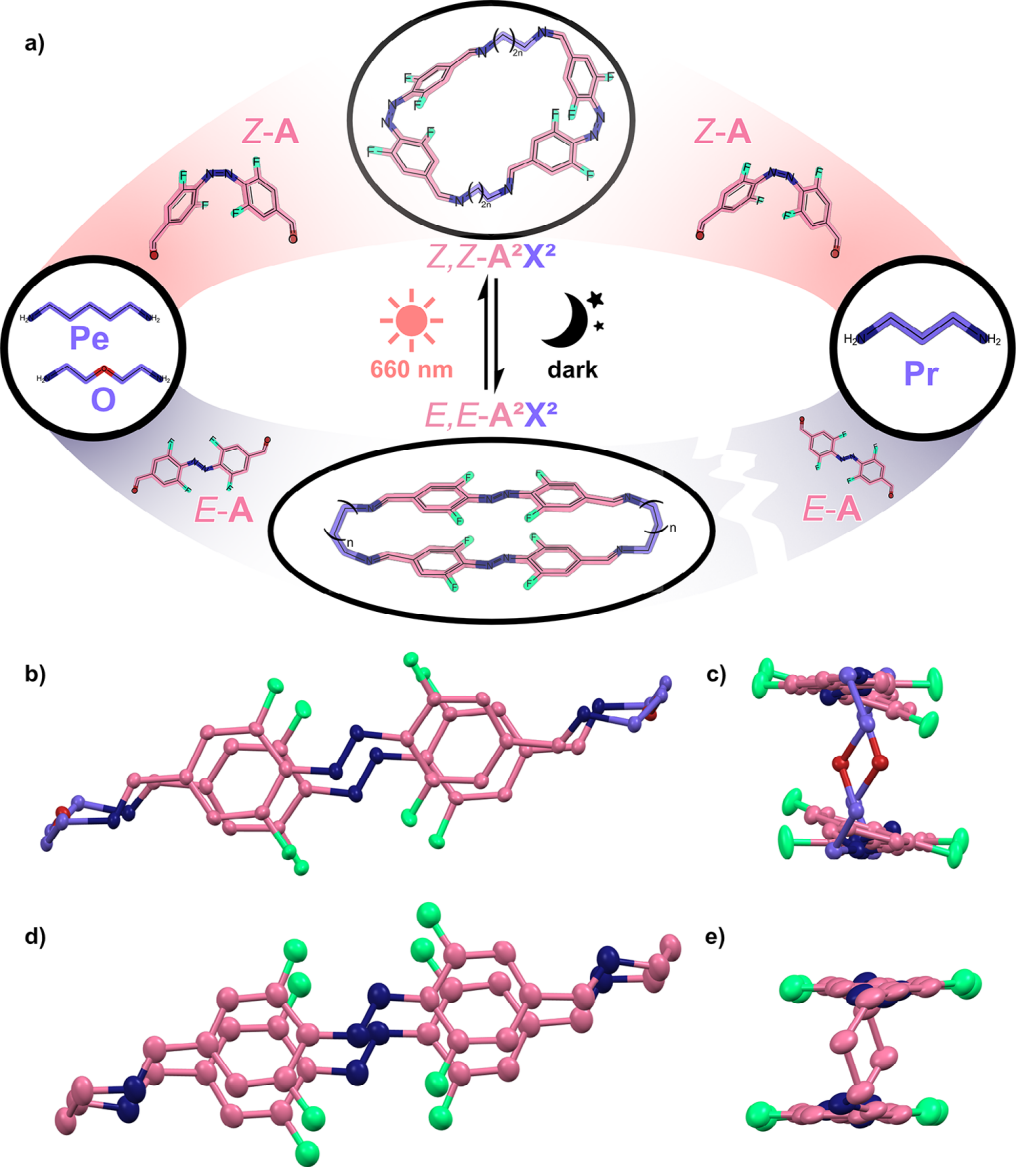
(a) Dynamic covalent assembly of **A^2^X^2^**‐type macrocycles from azobenzene **A** and diamines **Pr**, **Pe**, and **O**. *E*,*E*‐**A^2^X^2^**‐type macrocycles are only accessible with **Pe** and **O** in the dark, while **Pr** only gives access to oligomers. However, the kinetic *E*,*E*‐**A^2^Pr^2^** is accessible by a *Z*‐**A**‐mediated pathway from *Z*,*Z*‐**A^2^Pr^2^**. The molecular geometries were obtained from force field calculations;^[^ ^17^ ^]^ (b,c) one of the two polymorphs (*E*,*E*‐**A^2^O^2^**‐α and *E*,*E*‐**A^2^O^2^**‐β) obtained for the macrocycle *E*,*E*‐**A^2^O^2^** by SC‐XRD measurements.^[^ ^20^ ^]^ Here, *E*,*E*‐**A^2^O^2^**‐β is depicted; (d,e) molecular structure of *E*,*E*‐**A^2^Pr^2^** obtained by SC‐XRD measurements.^[^ ^20^ ^]^ All thermal ellipsoids set at 50% probability.

However, the majority of signals could not be attributed to distinct species but to oligomers varying in size and geometry (*r*_s_ between 5.27 and 9.40 Å). Likely, a mixture of different acyclic (rather than cyclic) imine oligomers of varying chain lengths formed (Figures S4–S9), which is in line with an increasing relative amount of acyclic species at higher concentrations.^[^ ^19^ ^]^ Thus, the condensation behavior of **Pr** with *E*‐**A** clearly differs from even‐numbered diamines **E**, **B**, and **H**. Irradiation of this complex mixture with red light led to the nearly sole formation of the *Z*,*Z*‐**A^2^Pr^2^** macrocycle (*r*_s_ = 5.59 Å), confirmed by a combination of NMR experiments and MS. This entity was also accessible by the addition of **Pr** to a *Z*‐**A**‐enriched solution and surprisingly served as a gateway to access *E*,*E*‐**A^2^Pr^2^**: *Z*/*E* isomerization of transient *Z*,*Z*‐**A^2^Pr^2^** led to the formation of kinetically trapped *E*,*E*‐**A^2^Pr^2^**. The formation of *E*,*E*‐**A^2^Pr^2^** was closely followed by ^19^F{^1^H} NMR experiments (Figure 2) and ultimately confirmed by single‐crystal x‐ray diffraction (SC‐XRD) measurement^[^ ^20^ ^]^ (Figure 3d,e). Investigating the reaction of *E*‐**A** with the next odd‐numbered homologue, pentane‐1,5‐diamine (**Pe**) (Figure 1b), again showed pronounced differences to **E**, **B**, and **H**. The major product of the reaction caused a sharp singlet at −119.15 ppm in the ^19^F{^1^H} NMR spectrum.

The shape of the signal as well as the smaller upfield shift, in comparison to the signals for the **A** *_**n**_* **Pe** *_**n**_* species, varied distinctively from the results obtained from the reactions of the other aliphatic diamines, which led to different overlapping signals with a larger upfield shift. This indicated that the preferred product of the reaction of diamine **Pe** and azobenzene isomer *E*‐**A** is not a mixture of different oligoimine species but a distinct macrocycle. This was further confirmed by MALDI‐MS, which showed a signal at *m*/*z* = 753.323, aligning with [**A** ^**2**^ **Pe** ^**2** ^+ H]^+^ (calc. 753.270), selectively forming a distinct macrocycle with *E*‐**A** in high NMR spectroscopic yields. Irradiation of *E*,*E*‐**A** ^**2**^ **Pe** ^**2**^ with red light leads to the quantitative isomerisation to *Z*,*Z*‐**A** ^**2**^ **Pe** ^**2**^, as can be seen by the ^19^F{^1^H} NMR singlet's downfield shift to −118.60 ppm, a practically identical mass spectrum, and comparable solvodynamic radii (*r*_s_(*E*,*E*‐**A** ^**2**^ **Pe** ^**2**^) = 6.12 Å and *r*_s_(*Z*,*Z*‐**A** ^**2**^ **Pe** ^**2**^) = 6.14 Å) obtained. Again, *Z*,*Z*‐**A** ^**2**^ **Pe** ^**2**^ was also accessible by the addition of **Pe** to a *Z*‐**A**‐enriched solution. A similar behaviour was previously observed by our group when combining **A** with the more rigid diamine **D**.^[^ ^8d^ ^]^ Since most attempts to isolate or crystallise macrocycles based on flexible diamines and **A** failed in our hands, with the exception of one structure obtained for *E*,*E*‐**A** ^**2**^ **Pr** ^**2**^, we also examined the reaction of diethylene glycol bisamine (**O**) with *E*‐**A**. **O** should be structurally comparable to **Pe**, however, with a certain degree of pre‐coordination, which led to the successful isolation of **A** ^**2**^ **X** ^**2**^‐type imine macrocycles in the past.^[^ ^21^ ^]^ As envisioned, the treatment of a solution of *E*‐**A** in CH_2_Cl_2_/EtOH with **O** led to the preferred formation of *E*,*E*‐**A** ^**2**^ **O** ^**2**^ that could also be crystallised (Figure 3b,c). The crystal structure of *E*,*E*‐**A** ^**2**^ **O** ^**2**[^ ^20^ ^]^ contains two crystallographically independent half macrocycles, one of which consists of noticeably twisted azobenzene units, the other one of more linear ones. A closer look at the **O** units reveals the reason for the preferred formation of *E*,*E*‐**A** ^**2**^ **O** ^**2**^ and *E*,*E*‐**A** ^**2**^ **Pe** ^**2**^: all torsion angles range between 58.7° and 70.8°, being very close to the stable *gauche* conformation with 60°. The *gauche* effect, which was thoroughly studied for similar structures like polyethylene oxide,^[^ ^22a^ ^]^ leads to a higher stability of the *gauche* conformation in comparison to the normally preferred *anti* conformation due to stereoelectronic effects.^[^ ^22b,c^ ^]^ *E*,*E*‐**A** ^**2**^ **O** ^**2**^ was switched to *Z*,*Z*‐**A** ^**2**^ **O** ^**2**^ with both red (660 nm) and green (565 nm) and vice versa using UV (405 nm) light (Figures S1–S3). The selective formation of both *E*,*E*‐ and *Z*,*Z*‐**A** ^**2**^ **X** ^**2**^ (**X** = **Pe**, **O**) and the photoswitching behaviour of **A** ^**2**^ **O** ^**2**^ indicate a similar behaviour to the structurally more rigid macrocycle **A** ^**3**^ **D** ^**3**[^ ^8d^ ^]^ and potentially expand the repertoire of out‐of‐equilibrium supramolecular assemblies with an efficient external mode of control. For *E*,*E*‐ and *Z*,*Z*‐**A** ^**2**^ **X** ^**2**^ (**X** = **Pe**, **O**), the macrocycle geometry can be controlled by visible light. A particularly remarkable case is the macrocycle *E*,*E*‐**A** ^**2**^ **Pr** ^**2**^, which cannot be formed from *E*‐**A** and **Pr** under given conditions (Figure 3a). However, it can be accessed after irradiating the system to the transient *Z*,*Z*‐**A** ^**2**^ **Pr** ^**2**^, which will then relax to *E,E*‐**A** ^**2**^ **Pr** ^**2**^, where this macrocycle may correspond to a kinetically trapped state rather than the global thermodynamic minimum on the potential energy surface of the system.

## 3 Conclusion

In conclusion, we have showcased the dynamic covalent chemistry of azobenzene **A** with *α*,*ω*‐diamines of variable chain lengths. While it is possible to generate box‐like macrocycles *Z*,*Z*‐**A^2^X^2^** between pincer‐like *Z*‐**A** and even‐numbered diamines **B** and **H** from either an oligomeric *E*‐**A**‐based resting state under irradiation or from *Z*‐**A**‐enriched solution, odd‐numbered homologues **Pr**, **Pe**, and **O** give access to a rich photochemistry of macrocyclic compounds. Both **Pe** and **O** form interconvertible **A^2^X^2^**‐type macrocycles with *E*‐ and *Z*‐**A**. The favourable formation of such assemblies with odd‐numbered diamines might be explained by the *gauche* effect^[^ ^22b,c^ ^]^ as shown in the crystal structure of *E*,*E*‐**A^2^O^2^**. However, the behaviour of diamine **Pr** is the odd one out for the observed even–odd alteration. It is possible that **Pr**, like **E**,^[^ ^8d^ ^]^ creates a mismatch with *E*‐**A** and is not flexible enough to form discrete and well‐defined macrocycles but requires a partially precoordinated match like *Z*‐**A** for macrocyclisation.^[^ ^22a^ ^]^ We believe that investigating the even–odd alteration of the reactivity between **A** and different diamines contributes to the development of various out‐of‐equilibrium supramolecular assemblies with diverse geometries, based on structurally simple but interconvertible monomers, giving access to a library of functional systems of increasing complexity.

## Supporting Information

The authors have cited additional references within the Supporting Information.^[^ ^23^, ^24^, ^25^, ^26^, ^27^, ^28^ ^]^

## Conflict of Interests

The authors declare no conflicts of interest.

## Supporting information



Supporting Information
