# Supplementary material for: Visible‐Light Switchable Rings and Chains in Dynamic Covalent Imine Chemistry
Source: Chemistry. 2025 Apr 7;31(27):e202501047. doi: 10.1002/chem.202501047 (PMC12080302; doi:10.1002/chem.202501047)
Supplement: Supplementary file 1 — Supporting Information [file CHEM-31-e202501047-s001.pdf]

Table of Contents

Abbreviations S1

I. General S2

II. Syntheses S3

III. UV/VIS spectroscopy S6

IV. Irradiation Studies S8

V. ^{19}F DOSY NMR measurements S41

VI. Crystallographic details S50

VII. NMR and MS Spectra S54

Abbreviations

A	Azobenzene: 4,4'-(diazene-1,2-diyl)bis(3,5-difluorobenzaldehyde)
B	Butane-1,4-diamine
E	Ethane-1,2-diamine
H	Hexane-1,6-diamine
O	Diethylene gycol bisamine
Pe	Pentane-1,5-diamine
Pr	Propane-1,3-diamine

I. General

Solvents and commercial starting materials were purchased from Sigma Aldrich, TCI, Fisher Scientific, J&K scientific, fluorochem, and abcr GmbH, and used as received. Dry solvents were obtained from an MBraun solvent purification system. Reactions were monitored by thin layer chromatography (TLC) carried out on silica gel plates (ALUGRAM® Xtra SIL G/UV254, Macherey Nagel) using UV light for detection. Column chromatography was carried out with silica gel (Silica 60 M, 0.04-0.063 mm, Macherey Nagel) using eluents as specified. Flash column chromatography was carried out on a Biotage® Selekt system using the SNAP Sphär60 columns.

NMR measurements

NMR spectra were recorded on a Bruker Avance III 300 and a Bruker Avance III 600 spectrometer at 25 °C, using residual protonated solvent signals as internal standards for ^{1}H spectra (^{1}H: δ($CDCl_3$) = 7.26 ppm), ^{19}F NMR spectra were referenced to external $CFCl_3$ at δ = 0.0 ppm.[1] Splitting patterns are abbreviated as follows: singlet (s), doublet (d), triplet (t), multiplet (m), and broad (br). ^{19}F DOSY NMR spectra were recorded on a Bruker Avance NEO 600 spectrometer at 20 °C.

The molar ratios discussed in this manuscript were determined by integration from ^{19}F{^{1}H} NMR spectra. Although decoupling potentially distorts the integrals due to the nuclear Overhauser effect (NOE), we have previously found that the molar ratios extracted from ^{19}F{^{1}H} NMR spectra are not significantly different from those extracted from ^{1}H NMR spectra (Schmidt et al., Supporting Information, Table S1–S2).[3]

Irradiation experiments

A 405 nm LED (M405L3), a 470 nm LED (M470L3), a 565 nm LED (M565L3), and a 660 nm LED (M660L4), together with an LED driver (LEDD1B), from Thorlabs were applied for photoisomerization. Irradiation times were determined using a stopwatch.

II. Syntheses

Azobenzene **A** was synthesised in analogy to Leistner *et al*.[2] as reported previously by our group.[3] Diethylene glycol bisamine (**O**) was synthesised following the procedure of Chiba *et al*.[4] with slight modifications, as described in detail below.

Diethylene glycol ditosylate (S1)

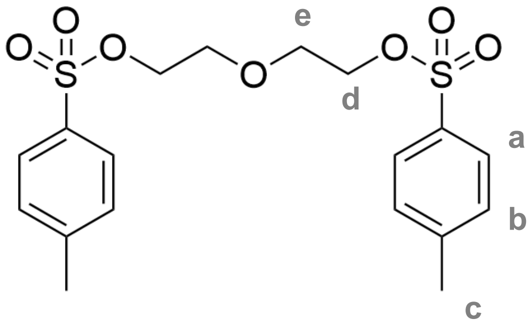

To a solution of diethylene glycol (5.7 mL, 60 mmol, 1.00 eq.) in CH_2Cl_2 (80 mL) were added tosyl chloride (34 g, 180 mmol, 3.00 eq.) and triethylamine (17.0 mL, 120 mmol, 2.00 eq.). The mixture turned green upon addition, and while stirring, the colour changed to black. The mixture was stirred for 19 hours at room temperature, during which the reaction turned brown. Water (100 mL) was added, and the layers were separated. The aqueous phase was extracted with EtOAc (2 x 50 mL), and the combined organic layers were dried over Na_2SO_4. The solvent was removed under reduced pressure, and the crude product was purified by column chromatography using CyHex and EtOAc (3:1). Tosylate **S1** (20 g, 49 mmol, 82%) was isolated as off-white crystals.

^{1}H NMR (300 MHz, $CDCl_3$, 298 K): δ [ppm] = 7.80 – 7.76 (m, 4H, H_a), 7.37 – 7.32 (m, 4H, H_b), 4.11 – 4.08 (m, 4H, H_e), 3.62 – 3.59 (m, 4H, H_d), 2.45 (s, 6H, H_c).
The spectra are in accordance with literature.[4]

Diethylene glycol bisazide (S2)

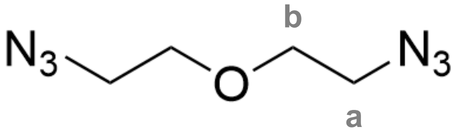

Tosylate **S1** (20.35 g, 49.10 mmol, 1.00 eq.) was dissolved in DMF (164 mL), and NaN_3 (7.980 g, 122.8 mmol, 2.50 eq.) was added in small portions. The mixture was stirred at rt for

62 hours, during which a colourless solid precipitated. Water (200 ml) was added, redissolving the solid, and the mixture was extracted with EtOAc (2 x 200 ml). The combined organic layers were dried over Na_2SO_4, and the solvents were removed under reduced pressure. Column chromatography using CyHex:EtOAc (1:1) yielded azide **S2** (7.200 g, 46.11 mmol, 94%) as a light yellow oil.

^{1}H NMR (300 MHz, $CDCl_3$, 298 K): δ [ppm] = 3.70 – 3.66 (m, 4H, H_b), 3.42 – 3.39 (m, 4H, H_a).
The spectra are in accordance with literature.[4]

Diethylene glycol bisamine (O)

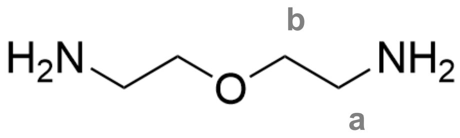

Azide **S2** (2.03 g, 13.0 mmol, 1.00 eq.) was dissolved in MeOH (25 mL), and 10wt% Pd/C (138 mg, 0.130 mmol, 10 mol%) was added. The mixture was stirred at rt under an H_2-atmosphere for 24 hours. The precipitate was filtered off, and the solvent was removed under reduced pressure. As ^{1}H NMR spectroscopic analysis of the crude material indicated incomplete conversion, the raw material was redissolved in EtOH (70 mL). 10wt% Pd/C (138 mg, 0.130 mmol, 10 mol%) was added, and the mixture was stirred at rt under an H_2-atmosphere for 3 days. The suspension was filtered and concentrated under reduced pressure, yielding diamine **O** (1.14 g, 11.0 mmol, 85%) as a light-yellow oil. The obtained product was stored at -20 °C under an N_2-atmosphere.

^{1}H NMR (300 MHz, CD_3OD, 298 K): δ [ppm] = 3.59 (t-like, 4H, H_b), 2.95 (s, 4H, H_a).
The spectra are in accordance with literature.[4]

E,E-**A²O²**

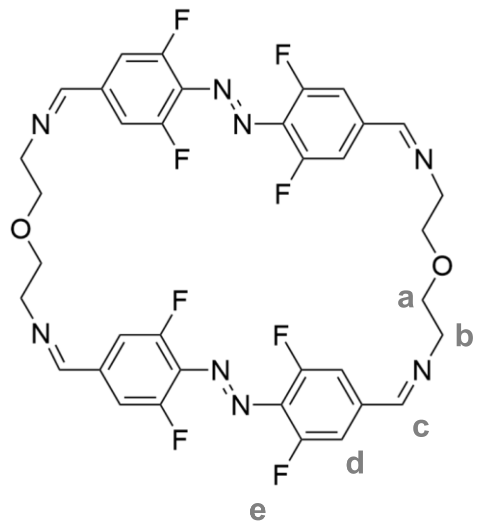

Azobenzene **A** (31.0 mg, 100 µmol, 1.00 eq.) was suspended in CH_2Cl_2 (5 mL), and a solution of **O** (12.0 mg, 120 µmol, 1.20 eq.) in EtOH (5 mL) was added in one portion. After stirring at rt for 5 days, the precipitate that had formed was collected by filtration and dried under reduced pressure. The crude product was recrystallised by vapor diffusion of Et_2O into a solution of the crude material in CH_2Cl_2 and EtOH, yielding *E,E*-**A²O²** (8.40 mg, 11.1 µmol, 22%) as dark red crystals.

^{1}H NMR (300 MHz, $CDCl_3$, 298 K): δ [ppm] = 8.04 (s, 4H, H_c), 7.14 – 7.11 (m, 8H, H_d), 3.79 (s, 16H, $H_{a\text{-}b}$); **^{19}F{^{1}H} NMR** (282 MHz, $CDCl_3$, 298 K): δ [ppm] = -118.74 (s, 4F, F_e); due to the low solubility of the compound and low stability in high concentrations, sufficient ^{13}C NMR data could not be obtained; high resolution **MS** (ESI) calc. for $[C_{36}H_{29}N_8F_8O_2]^+$: 757.2280, meas.: 757.2277; calc. for $[C_{36}H_{29}N_8F_8O_2]^{2+}$: 379.1177, meas.: 379.1176; **IR** $\tilde{\nu}$ [cm^{-1}]: 3082 (w), 2935 (w), 2922 (m), 2852 (m), 2803 (w), 1705 (m), 1647 (m), 1618 (m), 1570 (s), 1543 (s), 1375 (m), 1325 (m), 1196 (m), 1118 (m), 1042 (s), 924 (m), 858 (s), 795 (w), 748 (w), 696 (w), 633 (m).

III. UV/VIS spectroscopy

UV/VIS spectroscopy was performed on a Cary 60 equipped with a Peltier thermostatted cell holder. Quartz cuvettes (*d* = 10 mm) and solvents of spectrophotometric grade were used. Irradiation times were determined using a stopwatch. UV/VIS spectra were recorded at 25 °C unless otherwise stated. The concentration of *E*,*E*-**A²O²** employed was 16.7 μM.

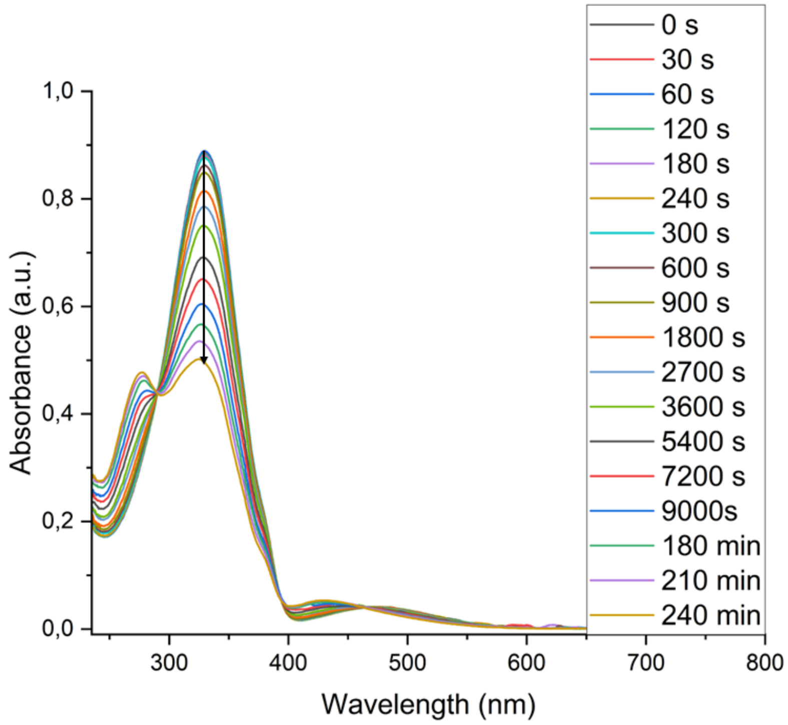

Figure S1: UV/VIS spectra of **A²O²** after irradiation with red light (660 nm) in CH_2Cl_2.

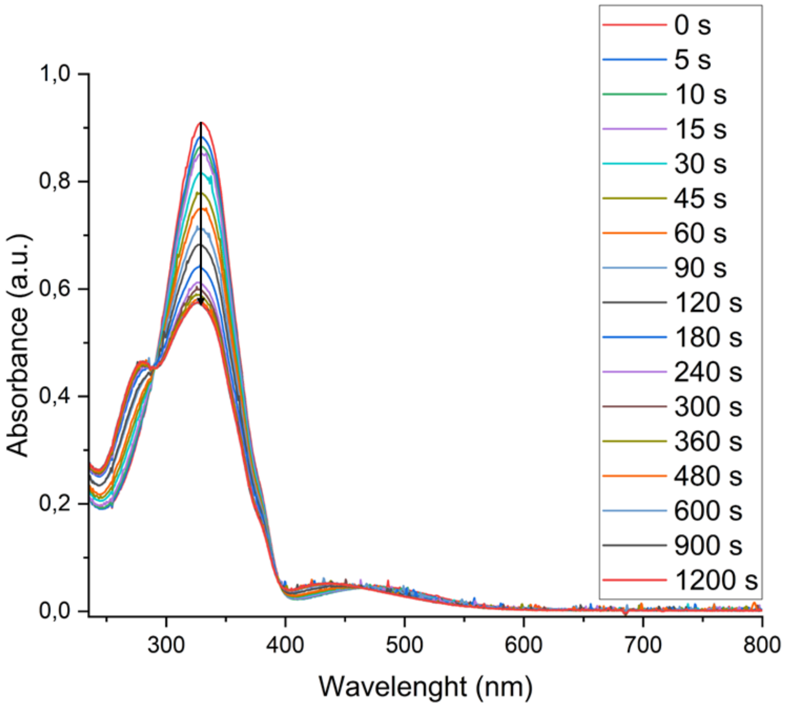

Figure S2: UV/VIS spectra of **A²O²** after irradiation with green light (565 nm) in CH_2Cl_2.

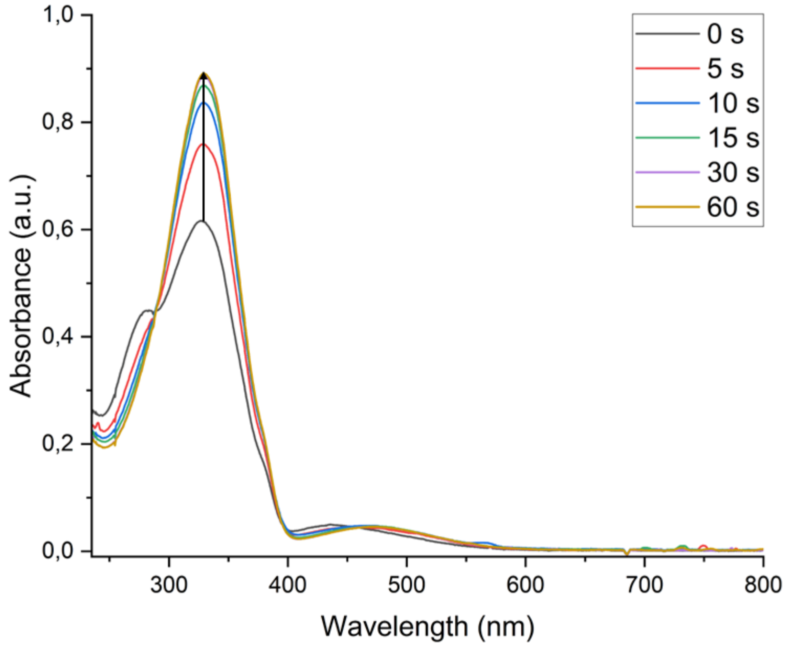

Figure S3: UV/VIS spectra of $\mathbf{A^2O^2}$. A solution that was *Z*-enriched by irradiation with green light was irradiated with light of the wavelength of 405 nm in CH_2Cl_2.

Table S1: Wavelengths of the absorption maxima λ_{max} and molar attenuation coefficients ε of the macrocycle *E*,*E*-$\mathbf{A^2O^2}$.

	E,E-$\mathbf{A^2O^2}$	
	π→π*	n→π*
λ_{max} / nm	330	486
ε / 10^3 L mol^{-1} cm^{-1}	54.4	3.44

IV. Irradiation Studies

Reactions of A with Pr, B, Pe and H at 5.0 mM

Stock solutions of the different diamines **Pr**, **B**, **Pe**, and **H** were prepared in $CDCl_3$. A stock solution of **A** was prepared in $CDCl_3$, and distributed into two parts; one part was kept in the dark while the other part was irradiated with red light (660 nm) until the percentage of *Z*-isomer was approximately 77% as determined by $^{19}F\{^1H\}$ NMR. The stock solutions of *E*-**A** and *Z*-**A** were mixed in an aldehyde to amine ratio of 1.0 to 1.2, and the mixtures were diluted to a total concentration of 5.0 mM in regard to the aldehyde **A**. The reactions containing *E*-**A** were stirred in the dark and then stirred under irradiation with red light. The vials containing *Z*-**A** were stirred under continuous irradiation. The reactions were monitored using ^1H and $^{19}F\{^1H\}$ NMR spectroscopy and MALDI-mass spectrometry. Reaction times and parameters are summarised in Table S2.

Table S2: Amount of starting materials and solvents employed in each reaction and reaction times.

Amine	Aldehyde	V($CDCl_3$)	Reaction time
**Pr** (0.44 mg, 6.0 µmol, 1.2 eq.)	*E*-**A** (1.6 mg, 5.0 µmol, 1.0 eq.)	1.0 mL	3 days (dark),<br>3 days (660 nm)
	Z-***A*** (1.6 mg, 5.0 µmol, 1.0 eq.)	1.0 mL	3 days
**B** (0.53 mg, 6.0 µmol, 1.2 eq.)	*E*-**A** (1.6 mg, 5.0 µmol, 1.0 eq.)	1.0 mL	3 days (dark),<br>3 days (660 nm)
	Z-***A*** (1.6 mg, 5.0 µmol, 1.0 eq.)	1.0 mL	3 days
**Pe** (1.2 mg, 12 µmol, 1.2 eq.)	*E*-**A** (3.1 mg, 10 µmol, 1.0 eq.)	2.0 mL	4 days (dark),<br>3 days (660 nm)
	Z-***A*** (3.1 mg, 10 µmol, 1.0 eq.)	2.0 mL	4 days
**H** (0.70 mg, 6.0 µmol, 1.2 eq.)	*E*-**A** (1.6 mg, 5.0 µmol, 1.0 eq.)	1.0 mL	3 days (dark),<br>3 days (660 nm)
	Z-***A*** (1.6 mg, 5.0 µmol, 1.0 eq.)	1.0 mL	3 days

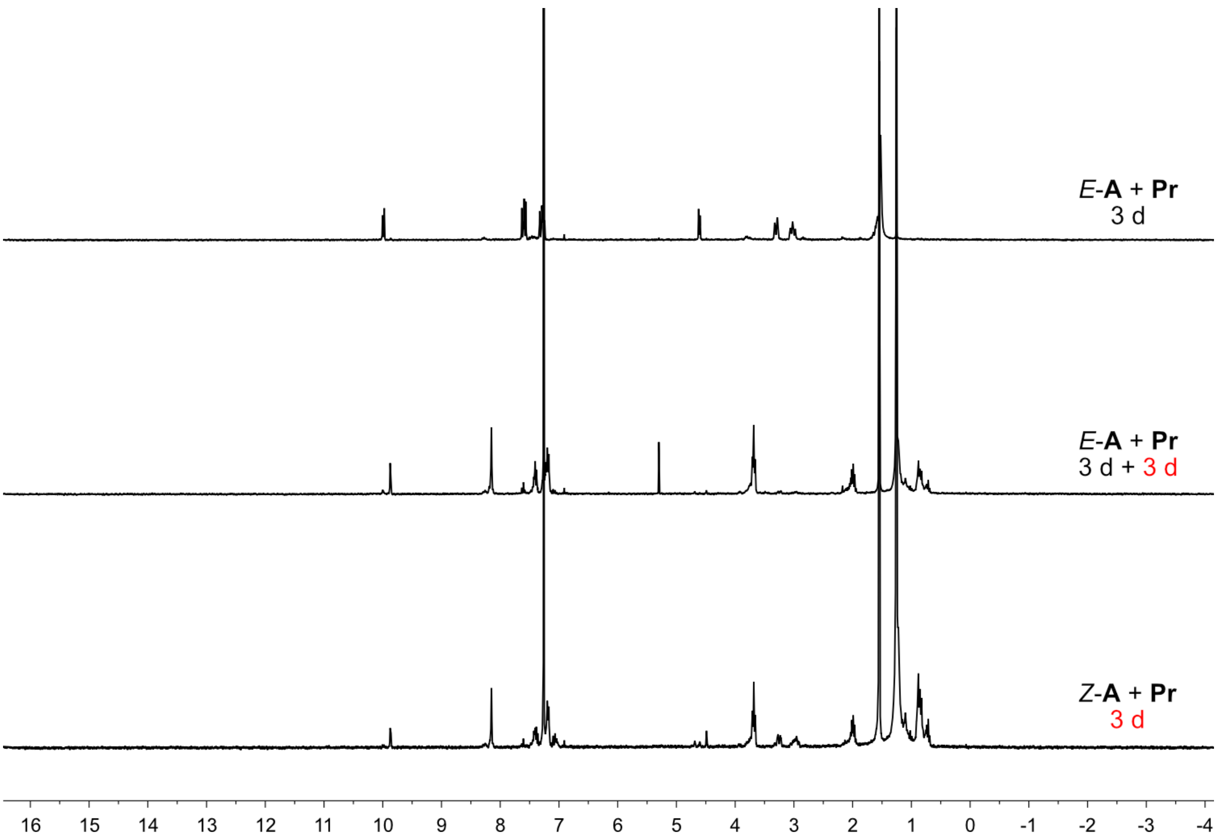

Figure S4: Comparison of ^{1}H NMR spectra ($CDCl_3$, 300 MHz) of *E*-**A** and **Pr** in the dark, after irradiation with red light (660 nm), and of *Z*-**A** and **Pr** after irradiation with red light.

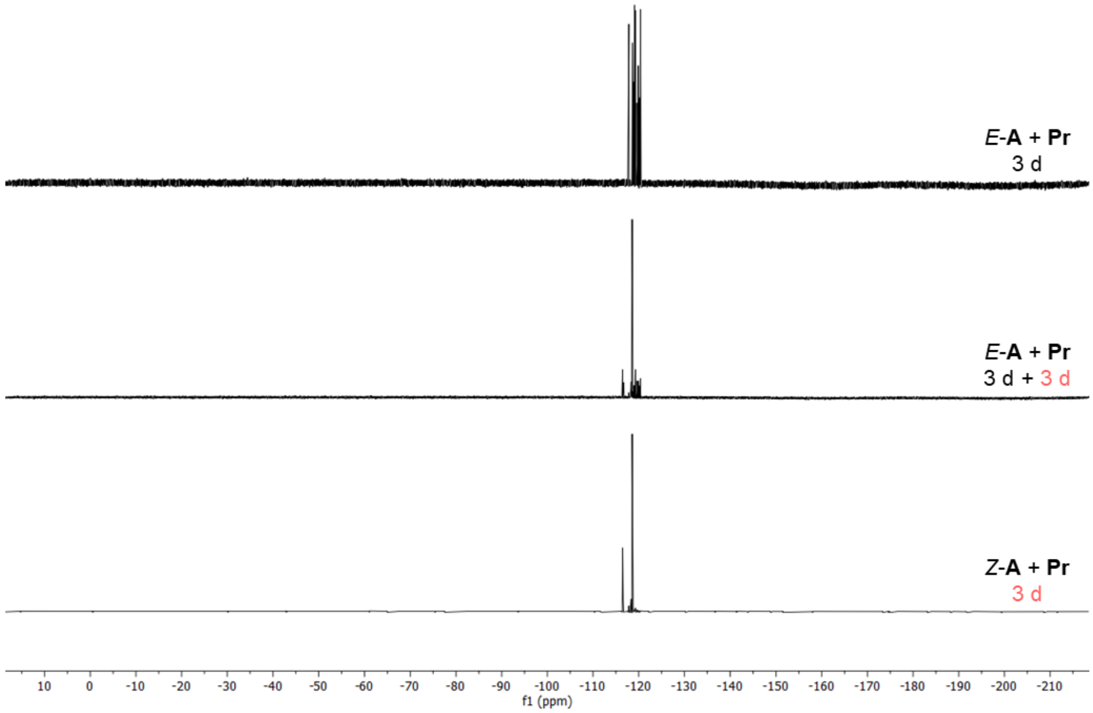

Figure S5: Comparison of ^{19}F{^{1}H} NMR spectra ($CDCl_3$, 282 MHz) of *E*-**A** and **Pr** in the dark, after irradiation with red light (660 nm), and of *Z*-**A** and **Pr** after irradiation with red light.

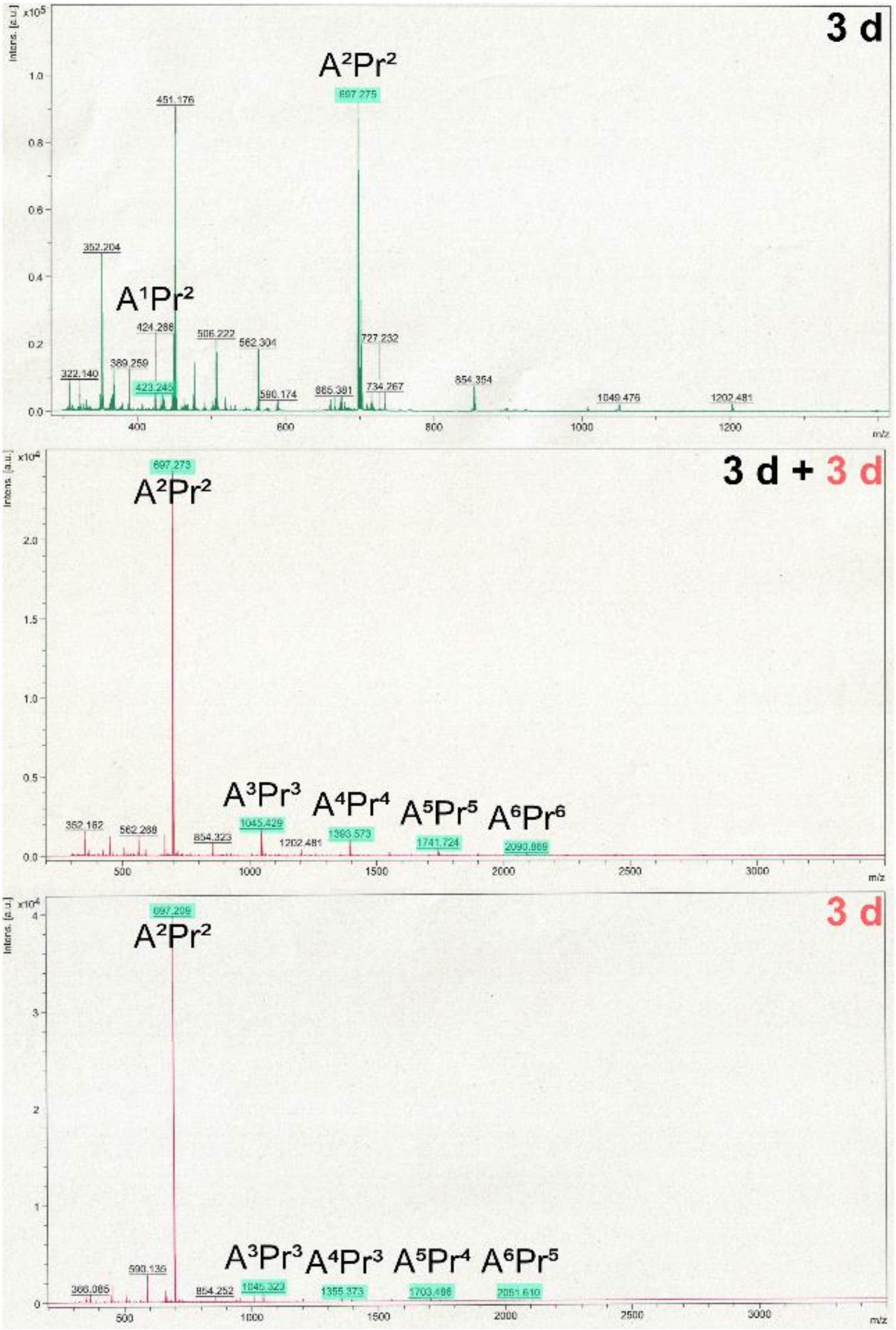

Figure S6: Comparison of MALDI-mass spectra of the reaction of *E*-**A** and **Pr** in the dark and under irradiation with red light (660 nm) and of the reaction of *Z*-**A** and **Pr** under continuous irradiation with red light. Peaks that can be attributed to **A^{n}Pr^n** compounds are highlighted in turquoise.

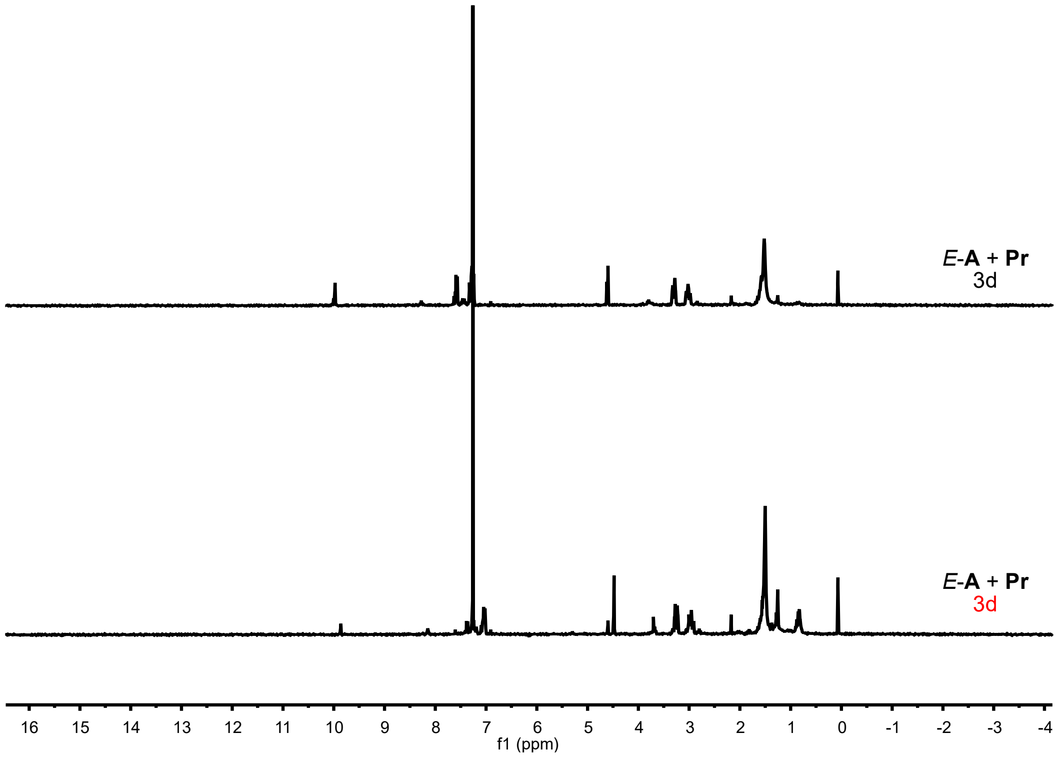

Figure S7: Comparison of ^{1}H NMR spectra ($CDCl_3$, 300 MHz) of *E*-**A** and **Pr** in the dark and after irradiation with red light (660 nm) with incomplete isomerisation.

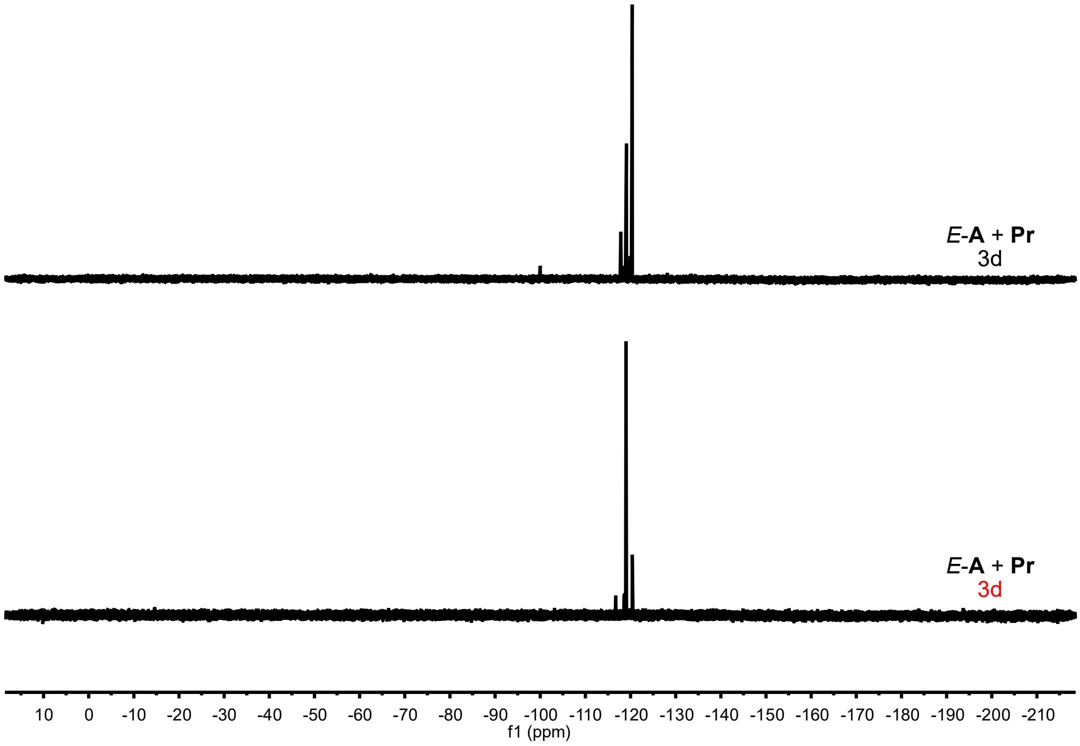

Figure S8: Comparison of ^{19}F{^{1}H} NMR spectra ($CDCl_3$, 282 MHz) of *E*-**A** and **Pr** in the dark and after irradiation with red light (660 nm) with incomplete isomerisation.

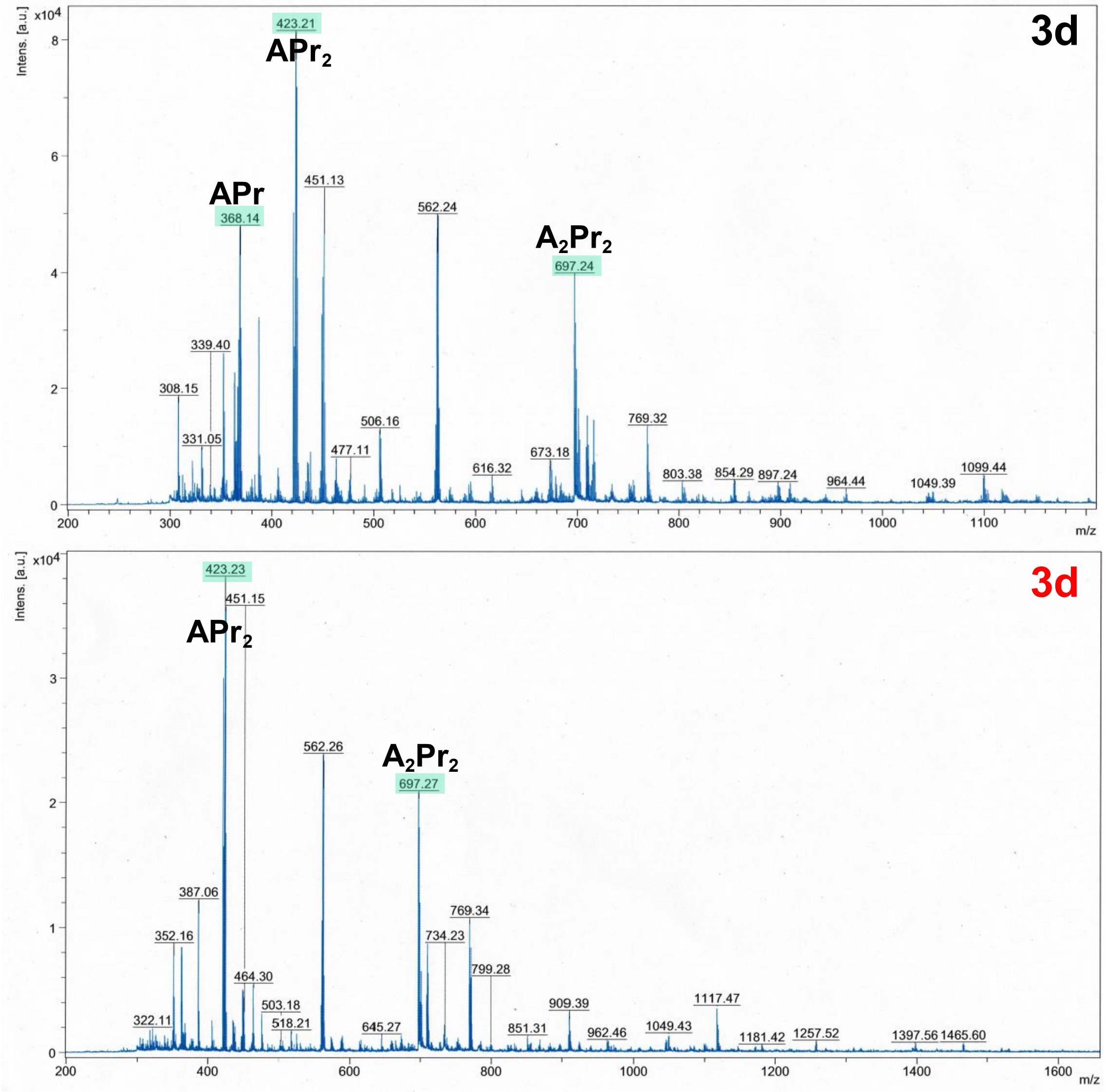

Figure S9: Comparison of MALDI-mass spectra of the reaction of *E*-**A** and **Pr** in the dark and after irradiation with red light (660 nm) with incomplete isomerisation. Peaks that can be attributed to **A**^{n}**Pr**^{n} compounds are highlighted in turquoise.

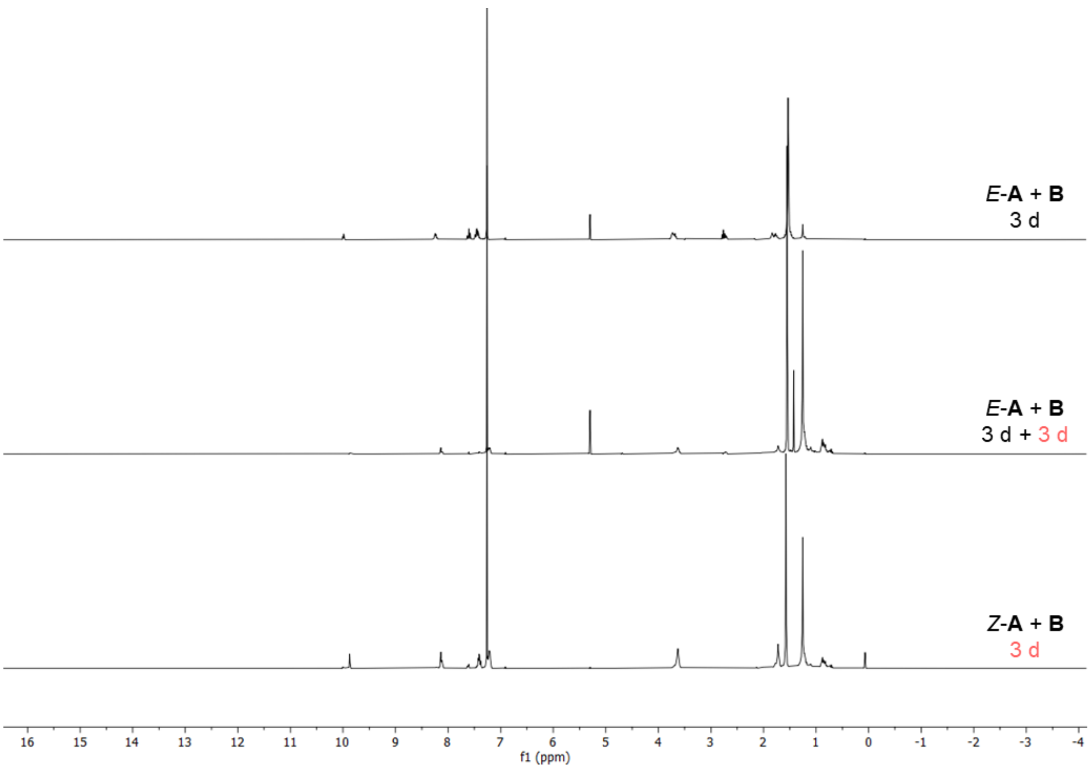

Figure S10: Comparison of ^{1}H NMR spectra ($CDCl_3$, 300 MHz) of *E*-**A** and **B** in the dark, after irradiation with red light (660 nm), and of *Z*-**A** and **B** after irradiation with red light.

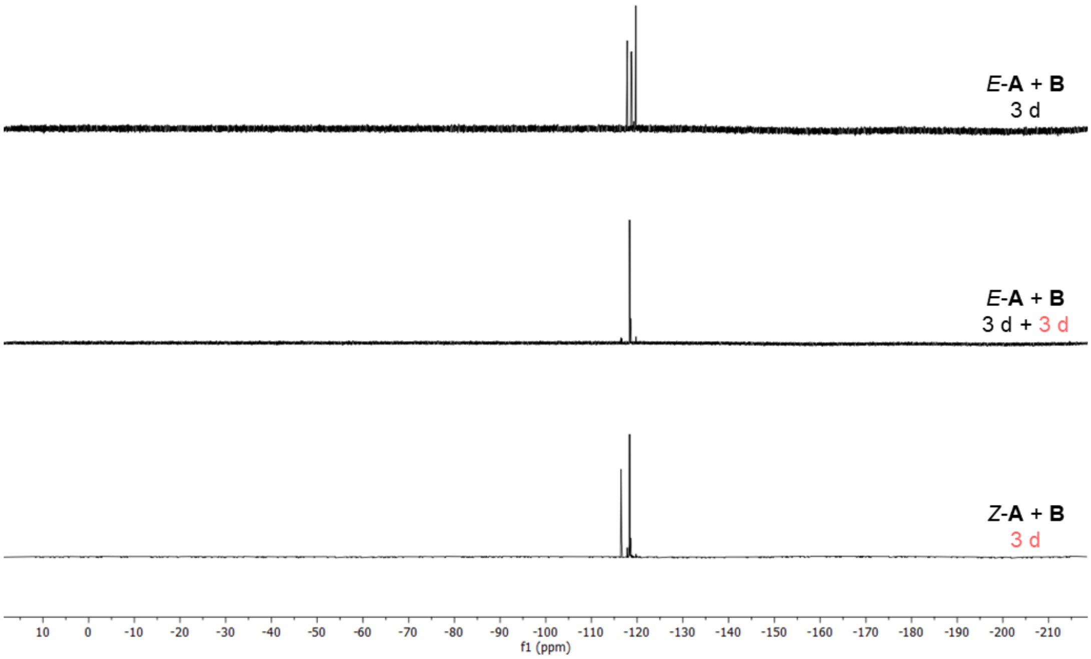

Figure S11: Comparison of $^{19}F\{^{1}H\}$ NMR spectra ($CDCl_3$, 282 MHz) of *E*-**A** and **B** in the dark, after irradiation with red light (660 nm), and of *Z*-**A** and **B** after irradiation with red light.

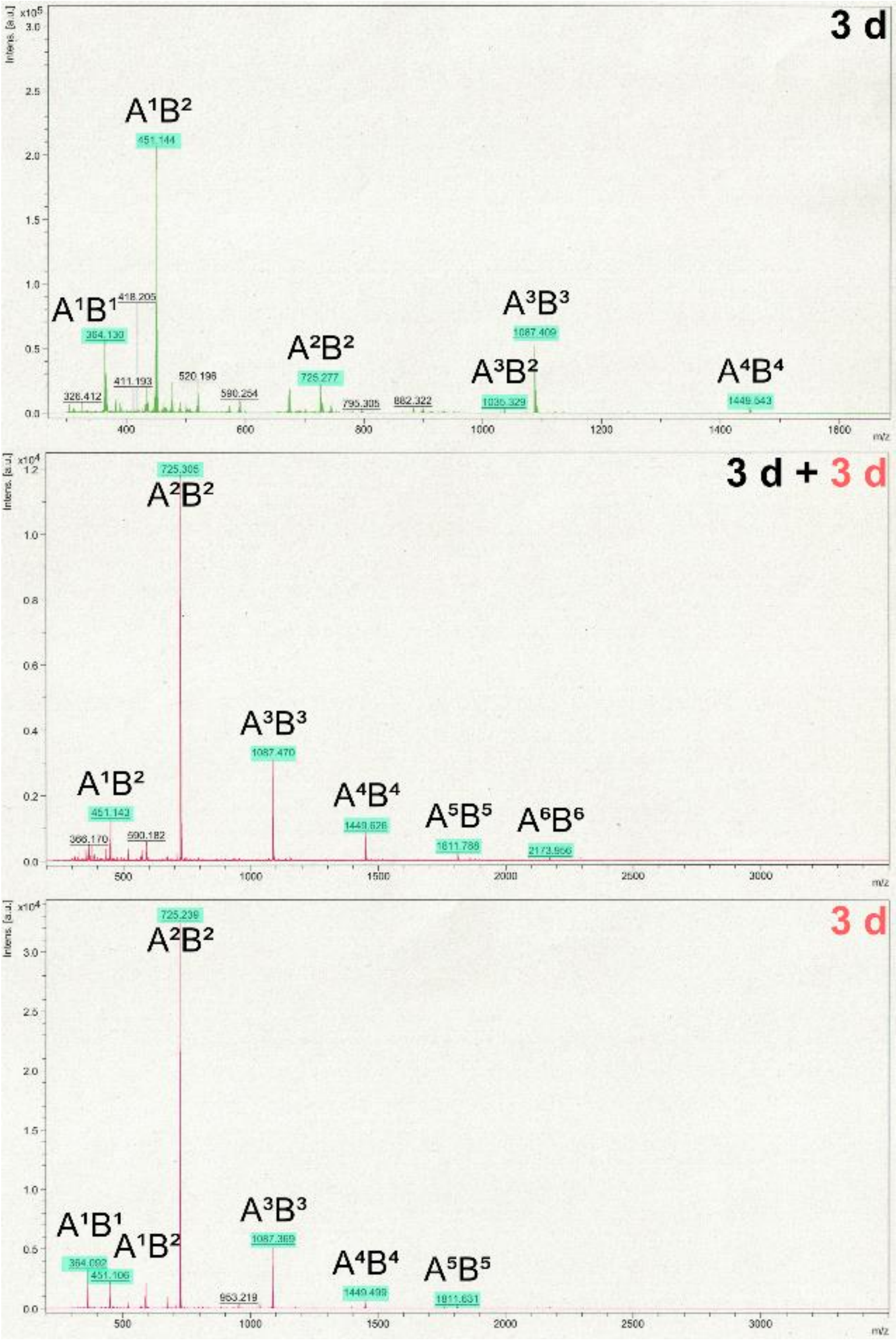

Figure S12: Comparison of MALDI-mass spectra of the reaction of *E*-**A** and **B** in the dark and under irradiation with red light (660 nm) and of the reaction of *Z*-**A** and **B** under continuous irradiation with red light. Peaks that can be attributed to $\mathbf{A^nB^n}$ compounds are highlighted in turquoise.

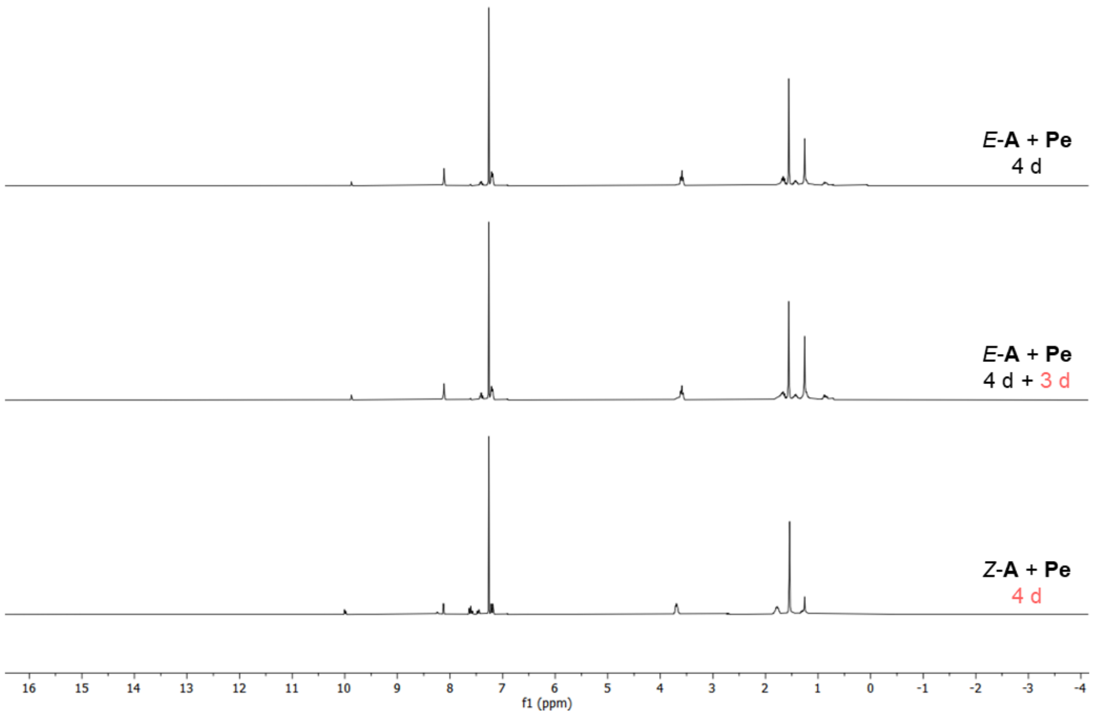

Figure S13: Comparison of ^{1}H NMR spectra ($CDCl_3$, 300 MHz) of *E*-**A** and **Pe** in the dark, after irradiation with red light (660 nm), and of *Z*-**A** and **Pe** after irradiation with red light.

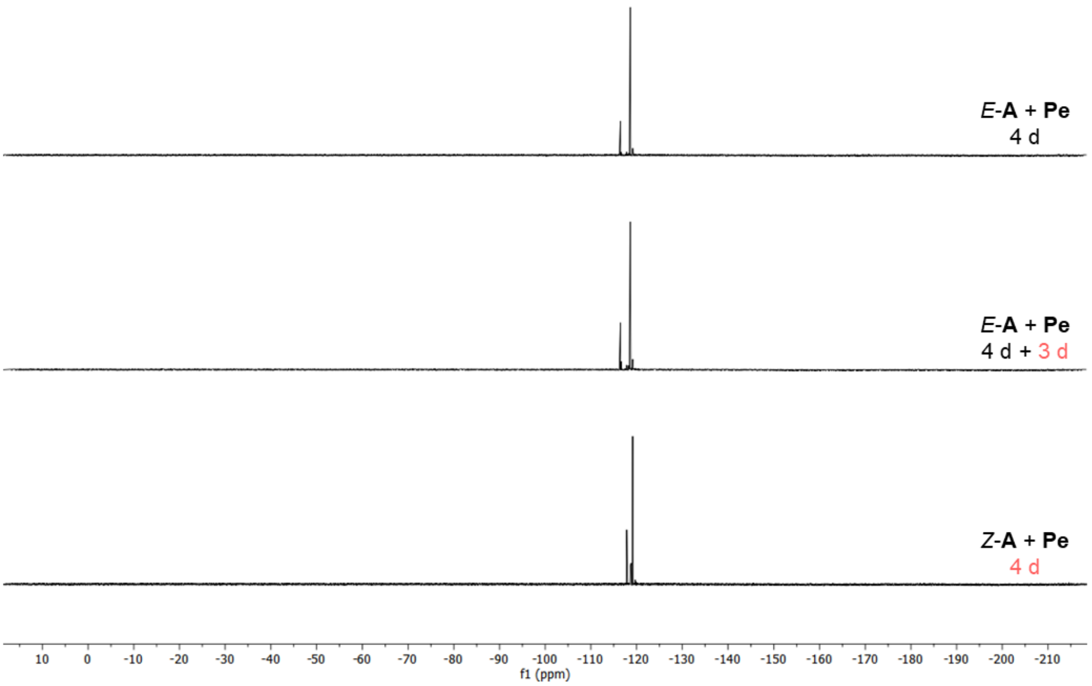

Figure S14: Comparison of $^{19}F\{^{1}H\}$ NMR spectra ($CDCl_3$, 282 MHz) of *E*-**A** and **Pe** in the dark, after irradiation with red light (660 nm), and of *Z*-**A** and **Pe** after irradiation with red light.

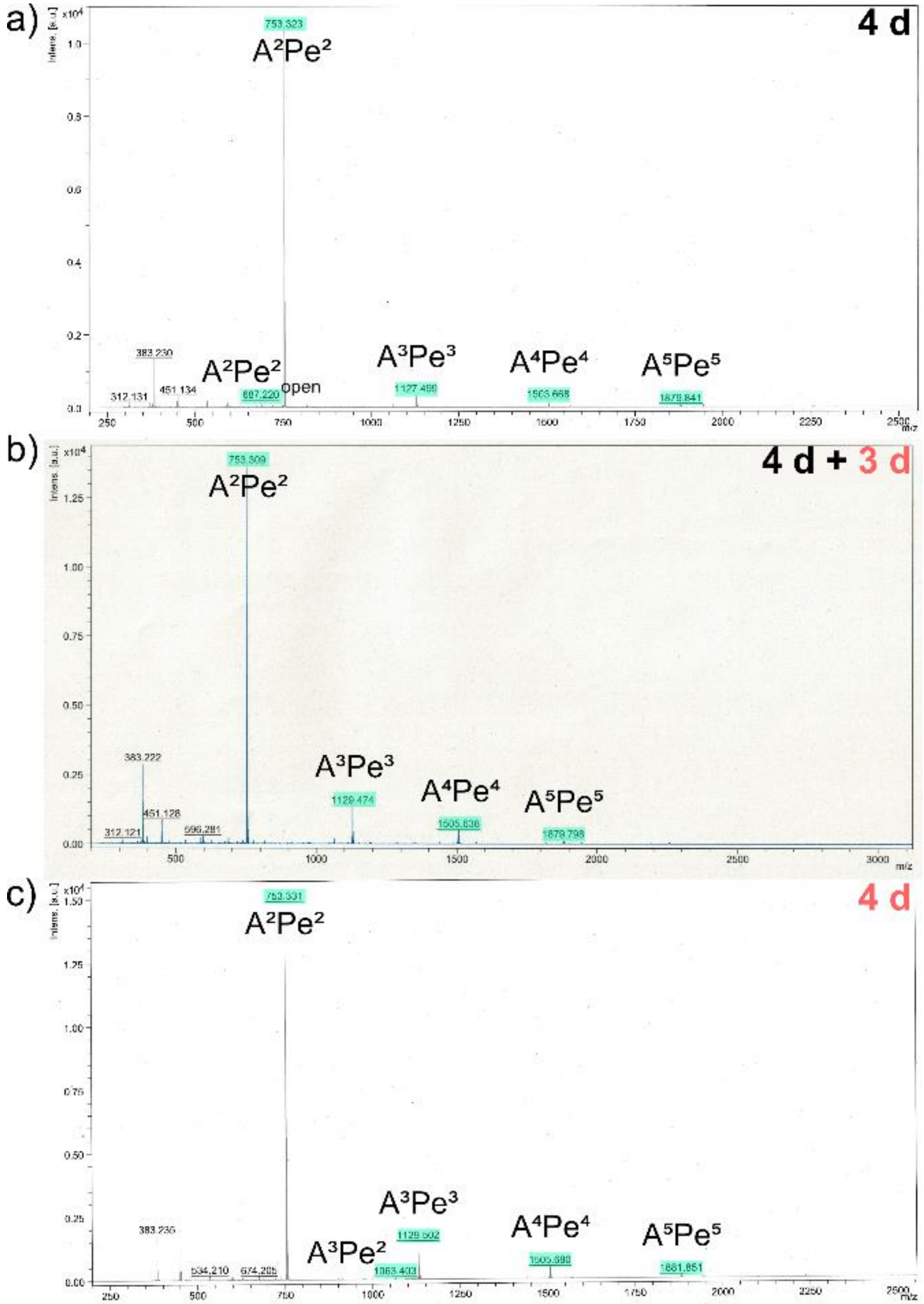

Figure S15: Comparison of MALDI-mass spectra of the reaction of *E*-**A** and **Pe** in the dark and under irradiation with red light (660 nm) and of the reaction of *Z*-**A** and **Pe** under continuous irradiation with red light. Peaks that can be attributed to $\mathbf{A}^n\mathbf{Pe}^n$ compounds are highlighted in turquoise.

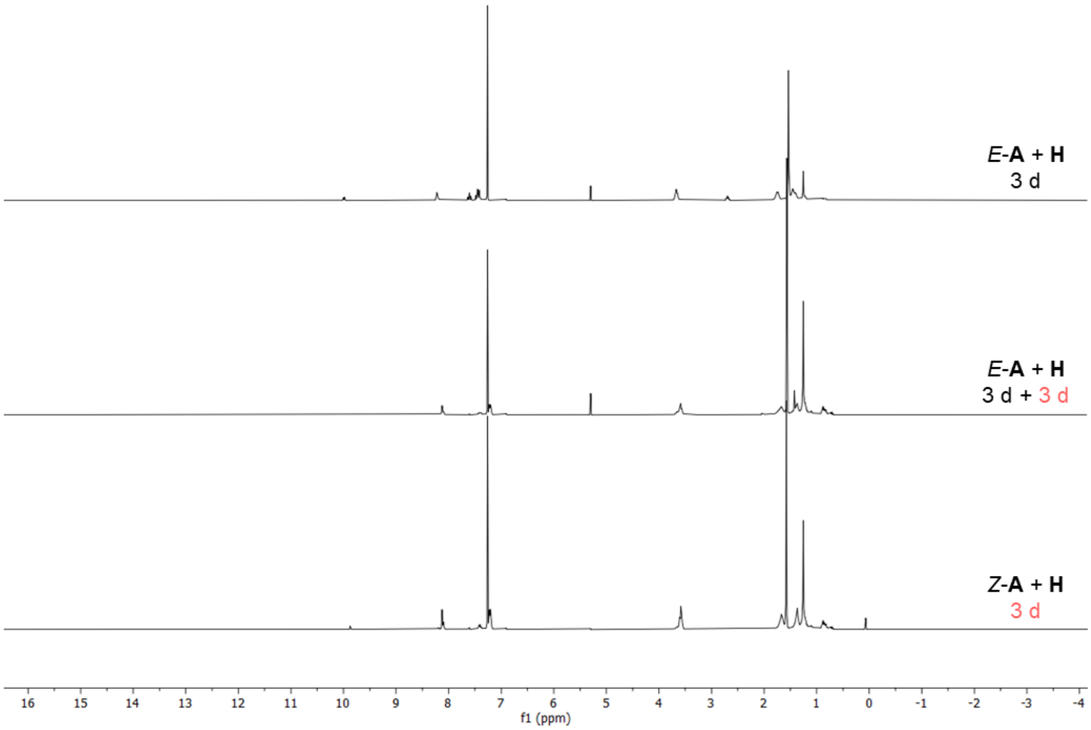

Figure S16: Comparison of ^{1}H NMR spectra ($CDCl_3$, 300 MHz) of *E*-**A** and **H** in the dark, after irradiation with red light (660 nm), and of *Z*-**A** and **H** after irradiation with red light.

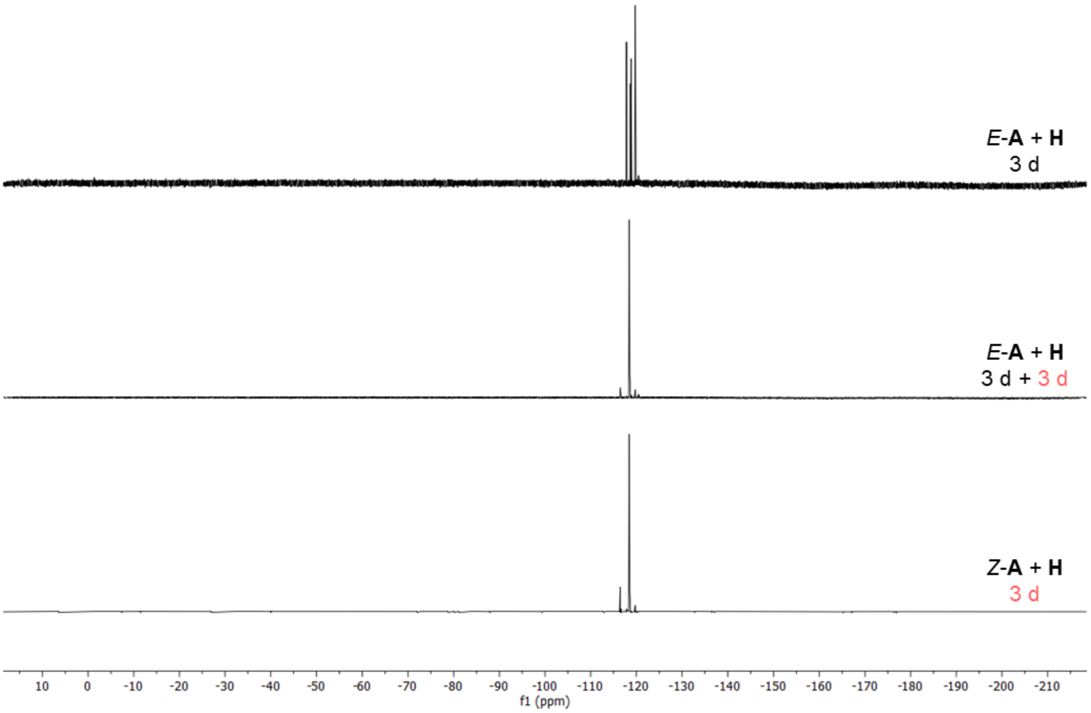

Figure S17: Comparison of $^{19}F\{^{1}H\}$ NMR spectra ($CDCl_3$, 282 MHz) of *E*-**A** and **H** in the dark, after irradiation with red light (660 nm), and of *Z*-**A** and **H** after irradiation with red light.

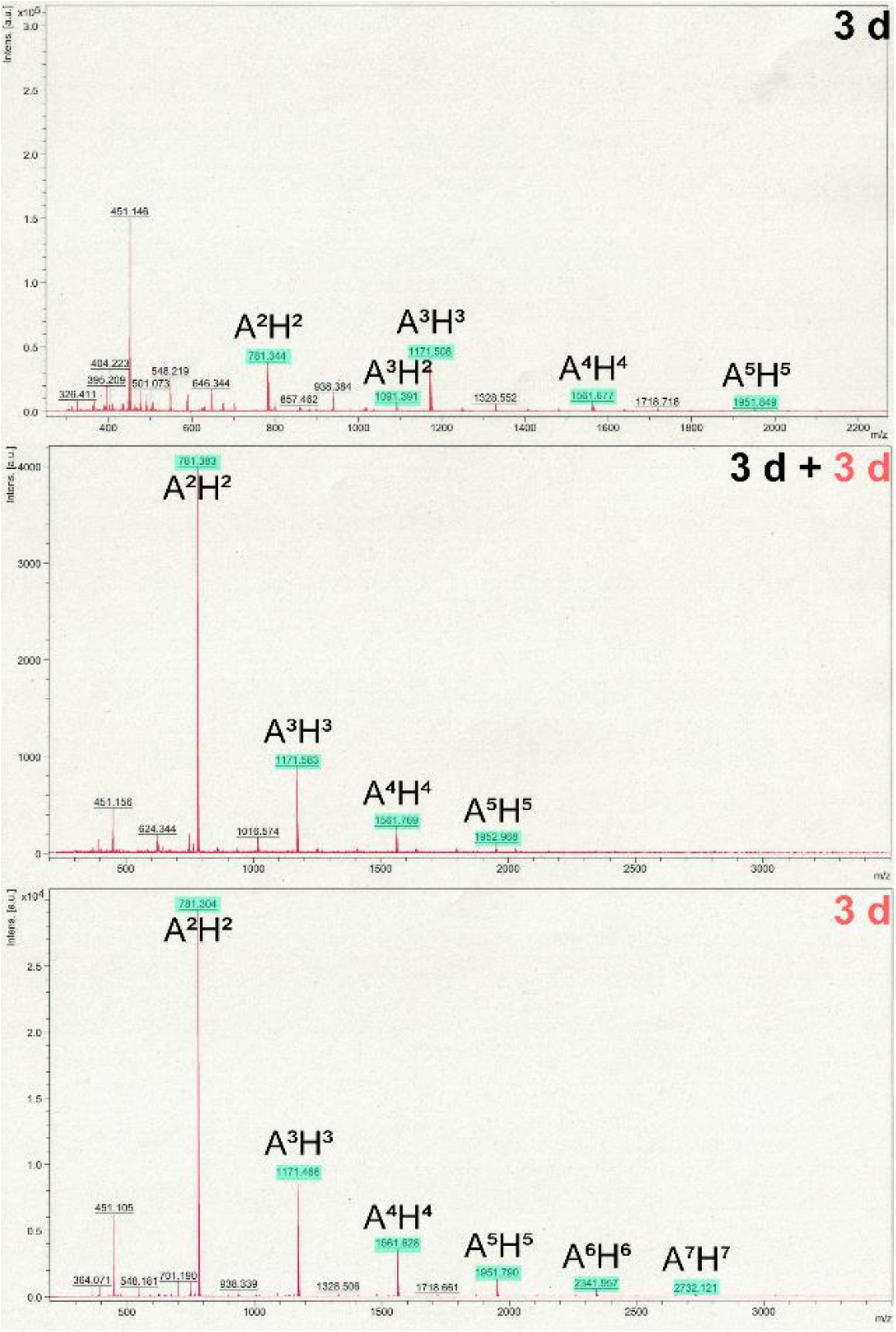

Figure S18: Comparison of MALDI-mass spectra of the reaction of *E*-**A** and **Pr** in the dark and under irradiation with red light (660 nm) and of the reaction of *Z*-**A** and **Pr** under continuous irradiation with red light. Peaks that can be attributed to $\mathbf{A}^n\mathbf{Pr}^n$ compounds are highlighted in turquoise.

Reaction of A with Pr, B, Pe and H at different concentrations

Stock solutions of the different diamines **Pr**, **B**, **Pe**, and **H** were prepared in $CDCl_3$. A stock solution of **A** was prepared in $CDCl_3$ for the samples with concentrations between 1 and 5 mM, and azobenzene **A** was weighed in for the samples with higher concentrations. Two samples of each combination and concentration were prepared according to the details listed in Table S3. The first half of those samples was kept in the dark, while the other half was irradiated with red light. The reactions were monitored by ^{1}H and ^{19}F NMR spectroscopy and MALDI mass spectrometry.

Table S3: Amount of starting materials and solvents employed in each reaction and reaction times.

Concentration	Aldehyde	Amine	V($CDCl_3$)	Reaction time
1.0 mM	**A** (0.25 mg, 0.80 µmol, 1.0 equiv.)	**Pr** (0.059 mg, 0.80 µmol, 1.0 equiv.)	0.8 mL	3 days (dark)
				3 days (660 nm)
		**B** (0.071 mg, 0.80 µmol, 1.0 equiv.)		3 days (dark)
				3 days (660 nm)
		**Pe** (0.082 mg, 0.80 µmol, 1.0 equiv.)		3 days (dark)
				3 days (660 nm)
		**H** (0.093 mg, 0.80 µmol, 1.0 equiv.)		3 days (dark)
				3 days (660 nm)
2.5 mM	**A** (0.62 mg, 2.0 µmol, 1.0 equiv.)	**Pr** (0.15 mg, 2.0 µmol, 1.0 equiv.)	0.8 mL	3 days (dark)
				3 days (660 nm)
		**B** (0.18 mg, 2.0 µmol, 1.0 equiv.)		3 days (dark)
				3 days (660 nm)
		**Pe** (0.20 mg, 2.0 µmol, 1.0 equiv.)		3 days (dark)
				3 days (660 nm)
		**H** (0.23 mg, 2.0 µmol, 1.0 equiv.)		3 days (dark)
				3 days (660 nm)
5.0 mM	**A** (1.2 mg, 24.0 µmol, 1.0 equiv.)	**Pr** (0.30 mg, 4.0 µmol, 1.0 equiv.)	0.8 mL	3 days (dark)
				3 days (660 nm)

		**B** (0.35 mg, 4.0 μmol, 1.0 equiv.)		3 days (dark)
				3 days (660 nm)
		**Pe** (0.41 mg, 4.0 μmol, 1.0 equiv.)		3 days (dark)
				3 days (660 nm)
		**H** (0.46 mg, 4.0 μmol, 1.0 equiv.)		3 days (dark)
				3 days (660 nm)
10 mM	**A** (2.5 mg, 8.0 μmol, 1.0 equiv.)	**Pr** (0.59 mg, 8.0 μmol, 1.0 equiv.)	0.8 mL	3 days (dark)
				3 days (660 nm)
		**B** (0.71 mg, 8.0 μmol, 1.0 equiv.)		3 days (dark)
				3 days (660 nm)
		**Pe** (0.82 mg, 8.0 μmol, 1.0 equiv.)		3 days (dark)
				3 days (660 nm)
		**H** (0.93 mg, 8.0 μmol, 1.0 equiv.)		3 days (dark)
				3 days (660 nm)
20 mM	**A** (5.0 mg, 16 μmol, 1.0 equiv.)	**Pr** (1.2 mg, 16 μmol, 1.0 equiv.)	0.8 mL	3 days (dark)
				3 days (660 nm)
		**B** (1.4 mg, 16 μmol, 1.0 equiv.)		3 days (dark)
				3 days (660 nm)
		**Pe** (1.6 mg, 16 μmol, 1.0 equiv.)		3 days (dark)
				3 days (660 nm)
		**H** (1.9 mg, 16 μmol, 1.0 equiv.)		3 days (dark)
				3 days (660 nm)

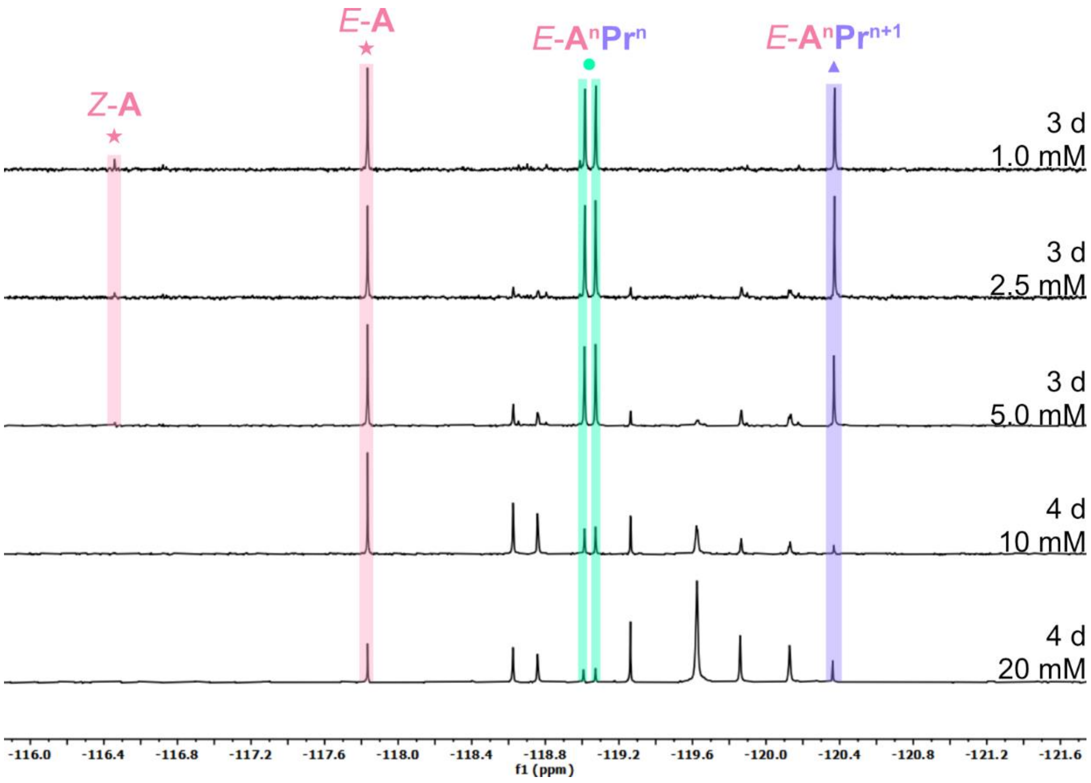

Figure S19: Comparison of $^{19}F\{^{1}H\}$ NMR spectra ($CDCl_3$, 282 MHz) of *E*-**A** and **H** at different concentrations after stirring in the dark.

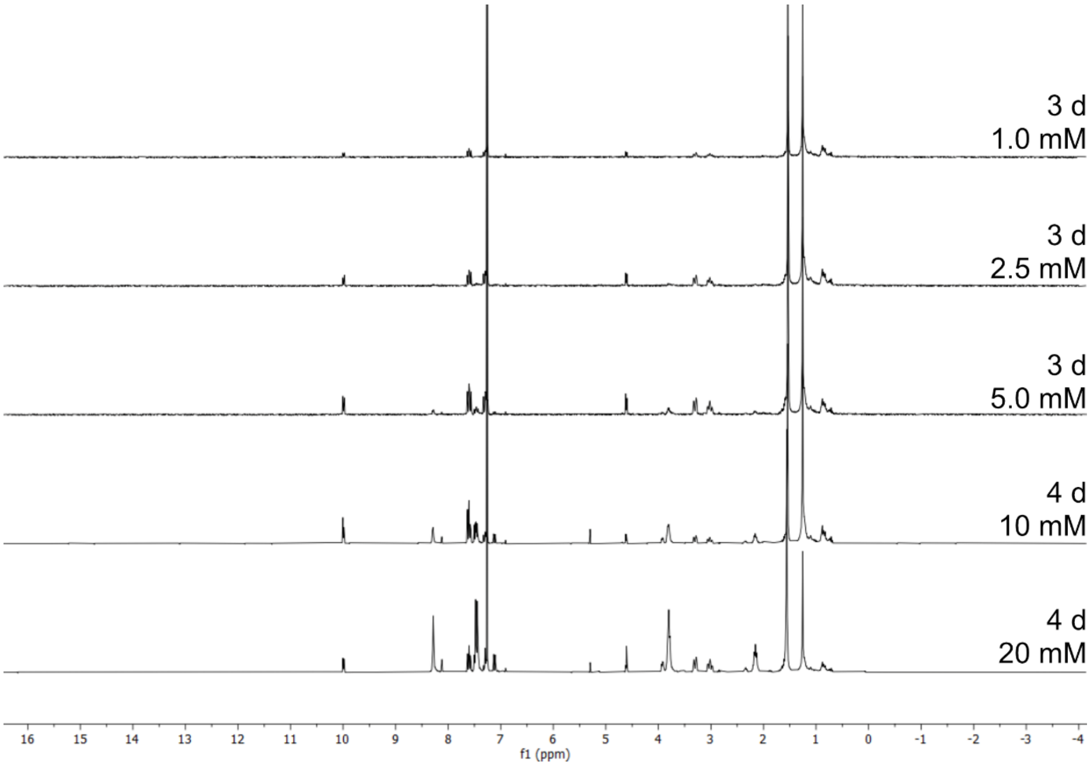

Figure S20: Comparison of ^{1}H NMR spectra ($CDCl_3$, 300 MHz) of *E*-**A** and **Pr** at different concentrations after stirring in the dark.

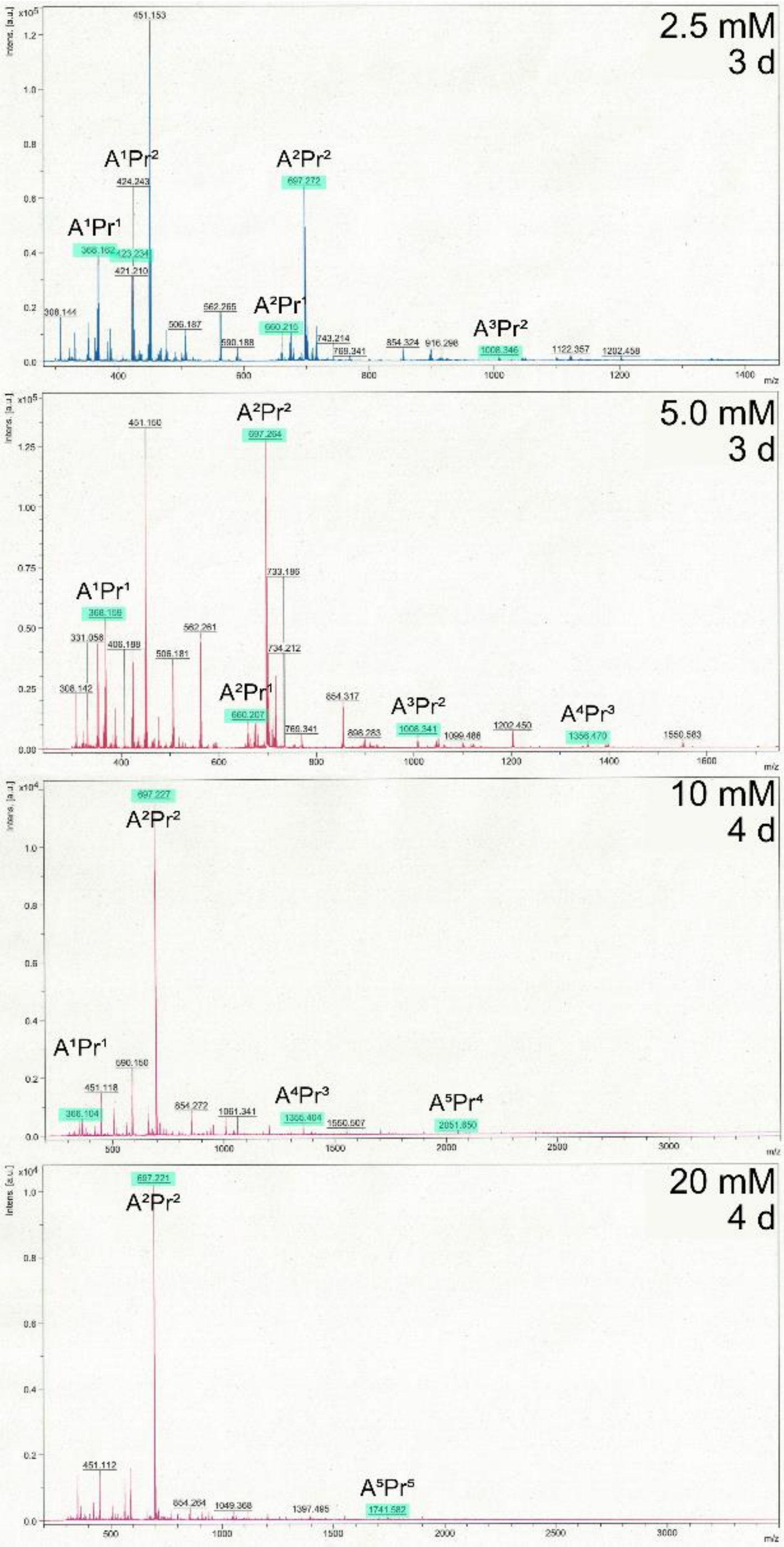

Figure S21: Comparison of MALDI-mass spectra of the reaction of *E*-**A** and **Pr** at different concentrations in the dark. Peaks that can be attributed to $\mathbf{A^nPr^n}$ compounds are highlighted in turquoise.

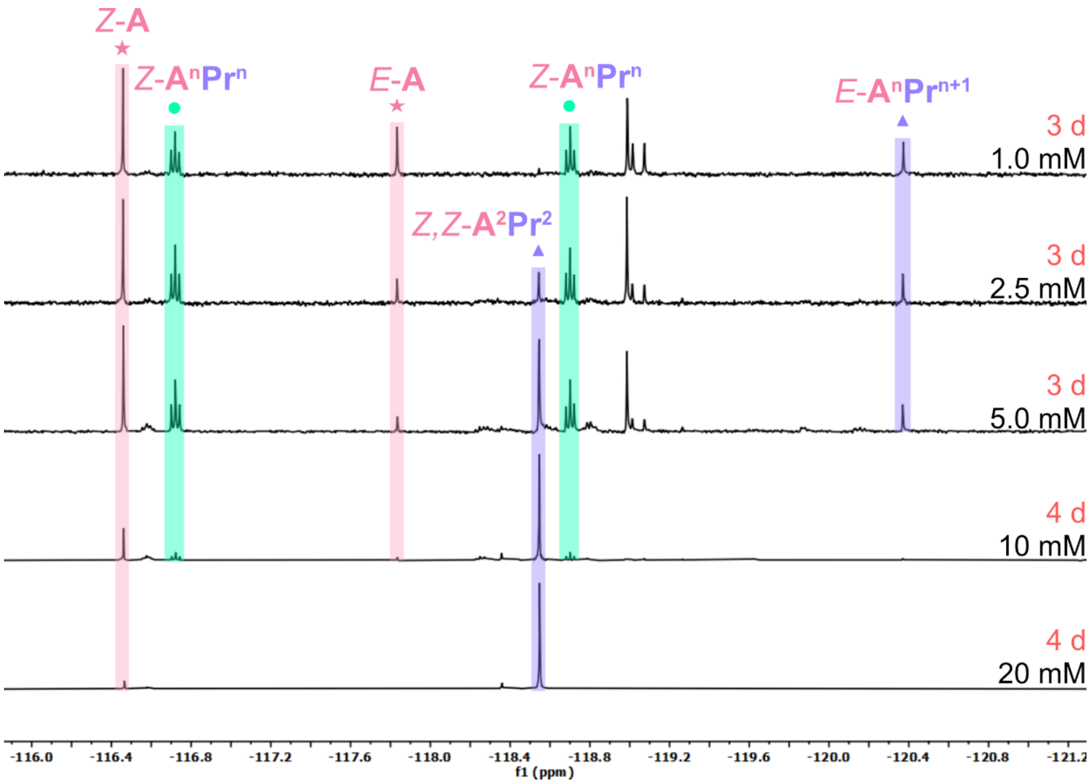

Figure S22: Comparison of ^{19}F{^{1}H} NMR spectra ($CDCl_3$, 282 MHz) of *E*-**A** and **Pr** at different concentrations after stirring under irradiation with red light.

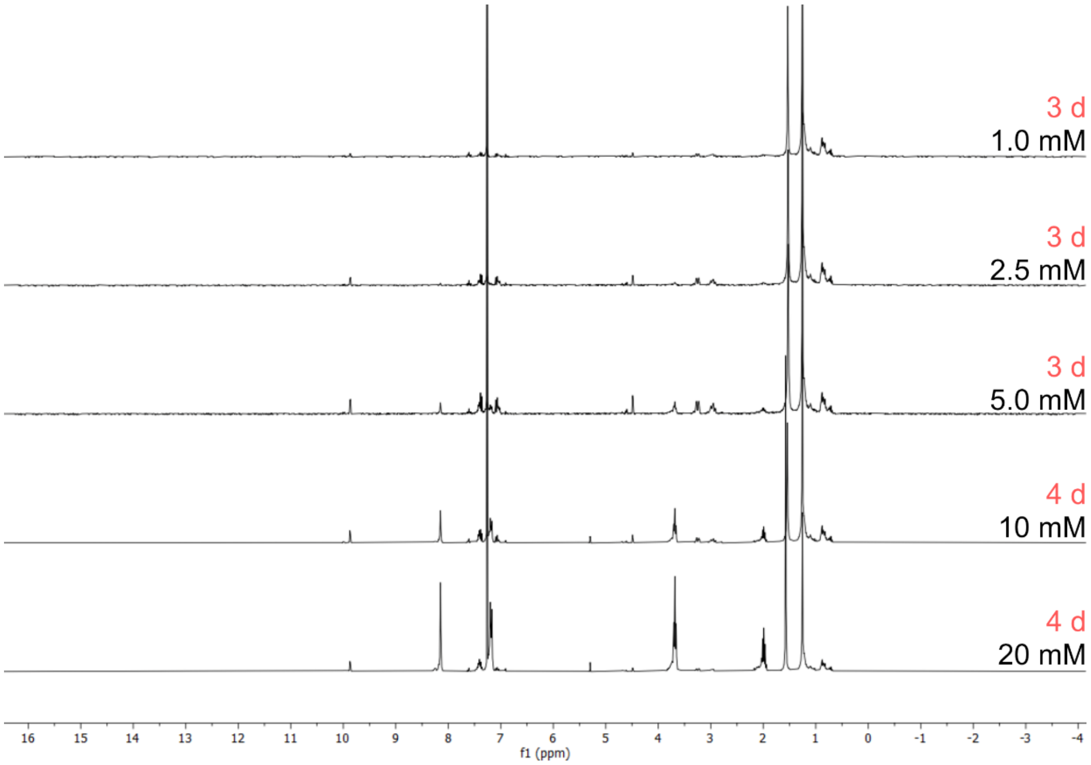

Figure S23: Comparison of ^{1}H NMR spectra ($CDCl_3$, 300 MHz) of *E*-**A** and **Pr** at different concentrations after stirring under irradiation with red light.

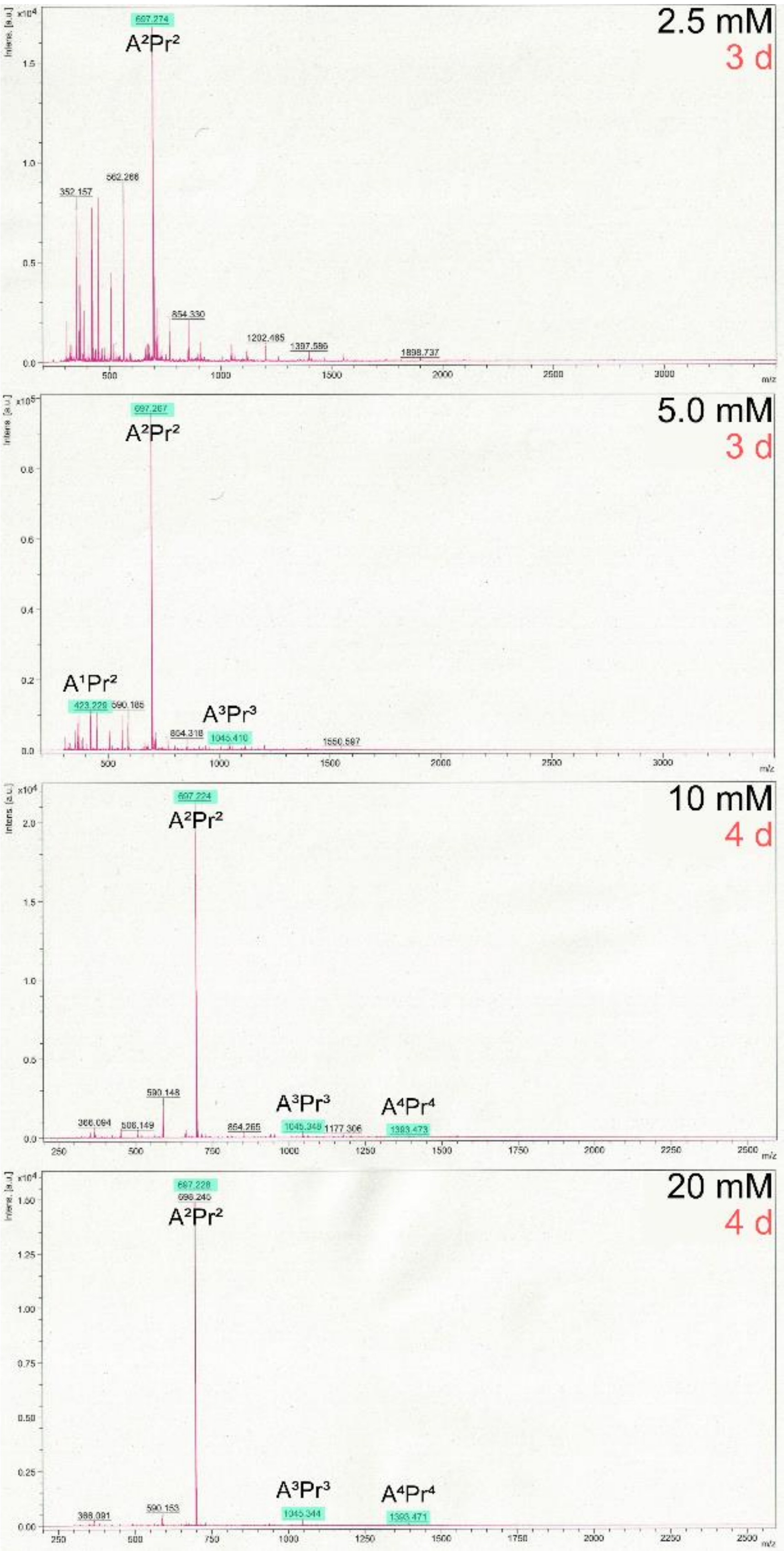

Figure S24: Comparison of MALDI-mass spectra of the reaction of *E*-**A** and **Pr** at different concentrations under irradiation with red light (660 nm). Peaks that can be attributed to $\mathbf{A^nPr^n}$ compounds are highlighted in turquoise.

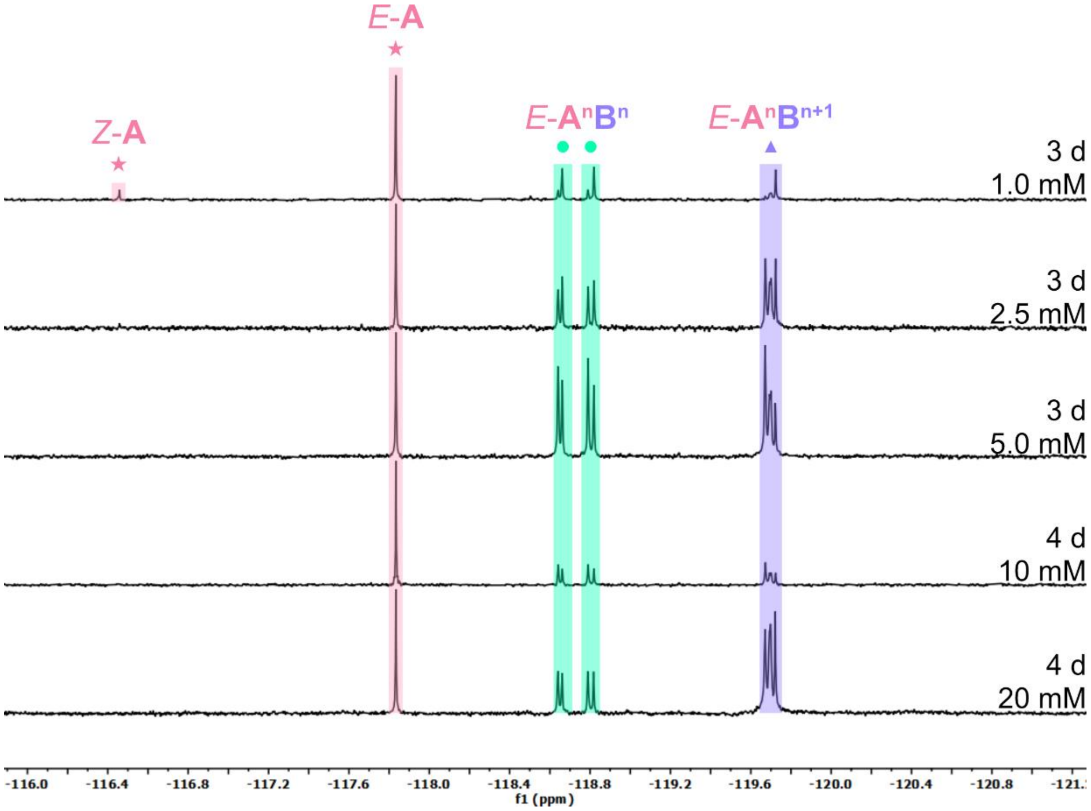

Figure S25: Comparison of $^{19}F\{^{1}H\}$ NMR spectra ($CDCl_3$, 282 MHz) of *E*-**A** and **B** at different concentrations after stirring in the dark.

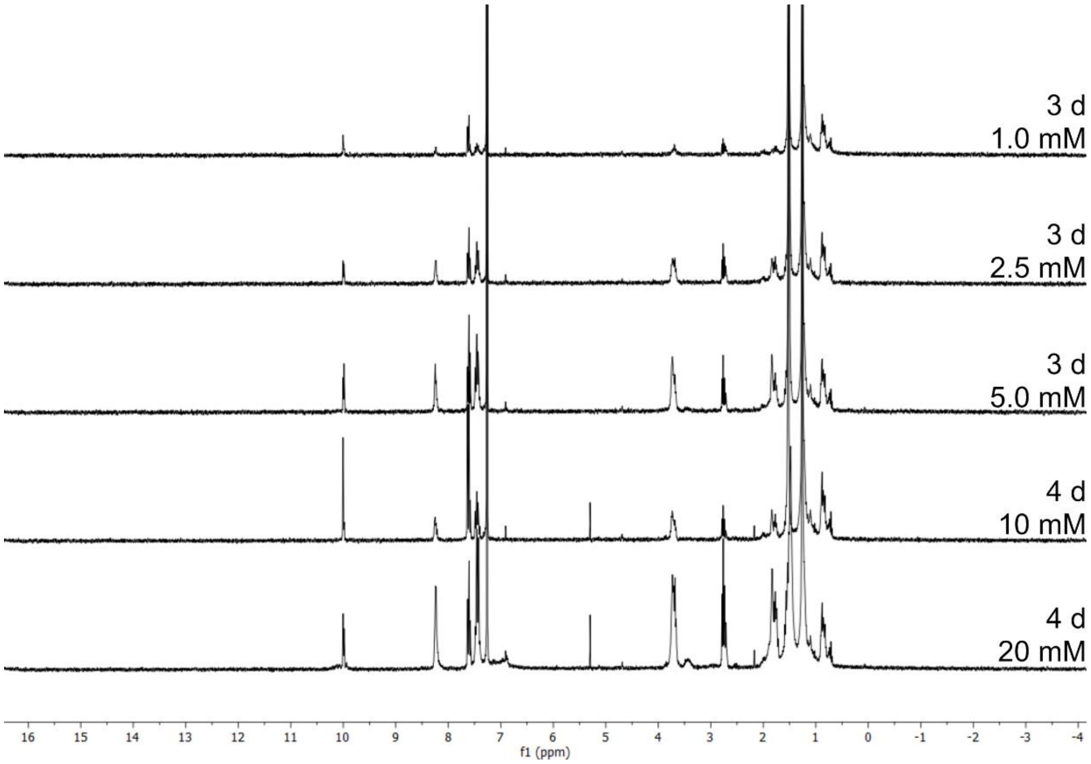

Figure S26: Comparison of ^{1}H NMR spectra ($CDCl_3$, 300 MHz) of *E*-**A** and **B** at different concentrations after stirring in the dark.

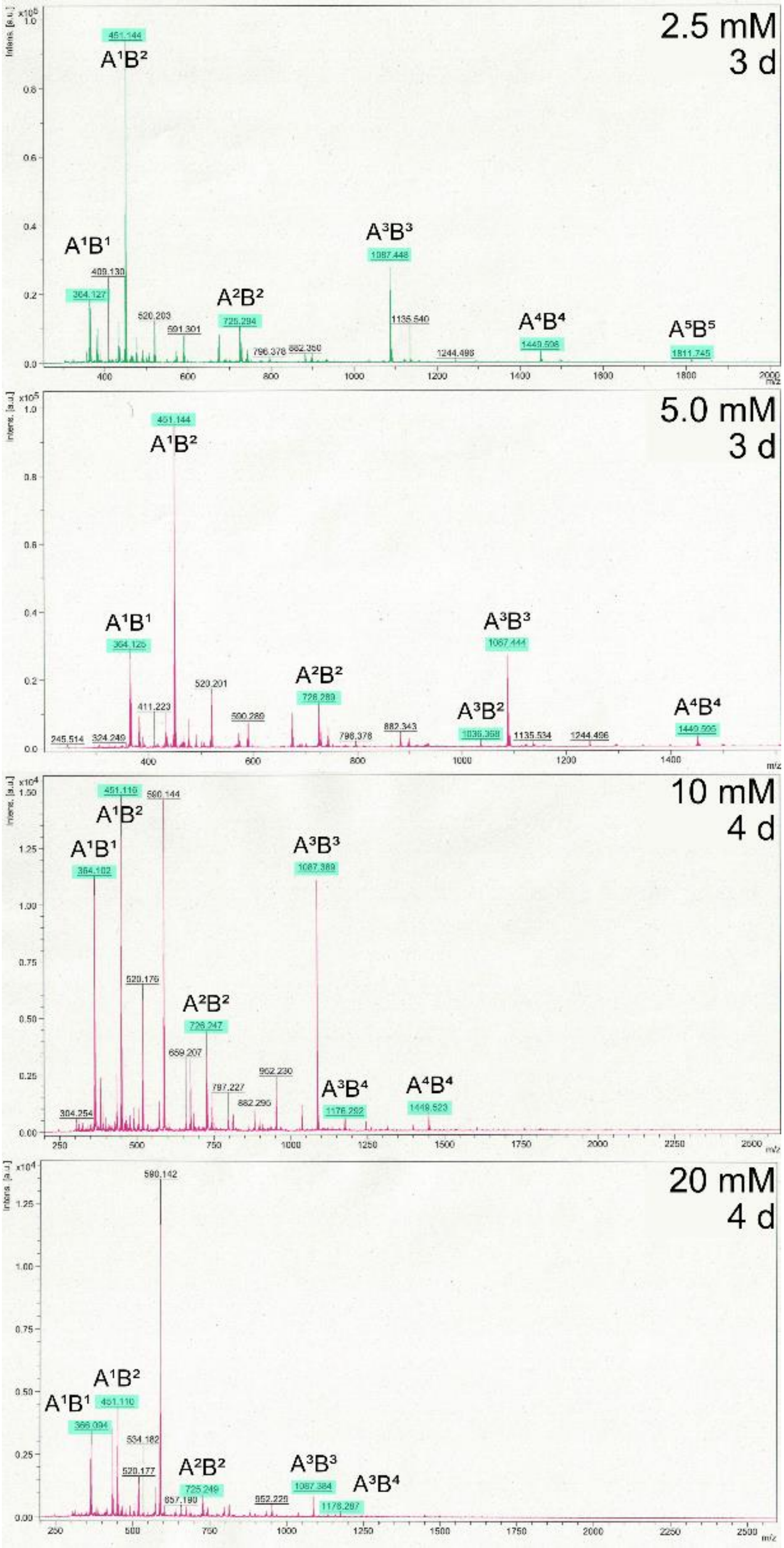

Figure S27: Comparison of MALDI-mass spectra of the reaction of *E*-**A** and **B** at different concentrations in the dark. Peaks that can be attributed to $\mathbf{A}^n\mathbf{B}^n$ compounds are highlighted in turquoise.

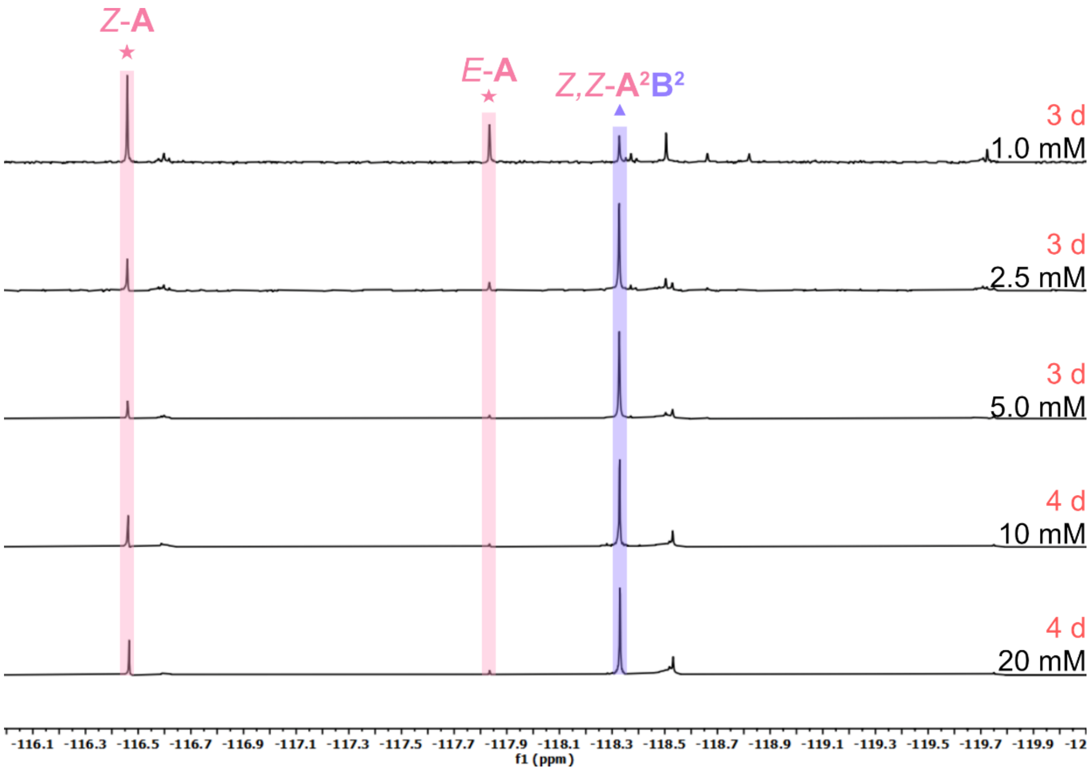

Figure S28: Comparison of $^{19}F\{^{1}H\}$ NMR spectra ($CDCl_3$, 282 MHz) of *E*-**A** and **B** at different concentrations after stirring under irradiation with red light.

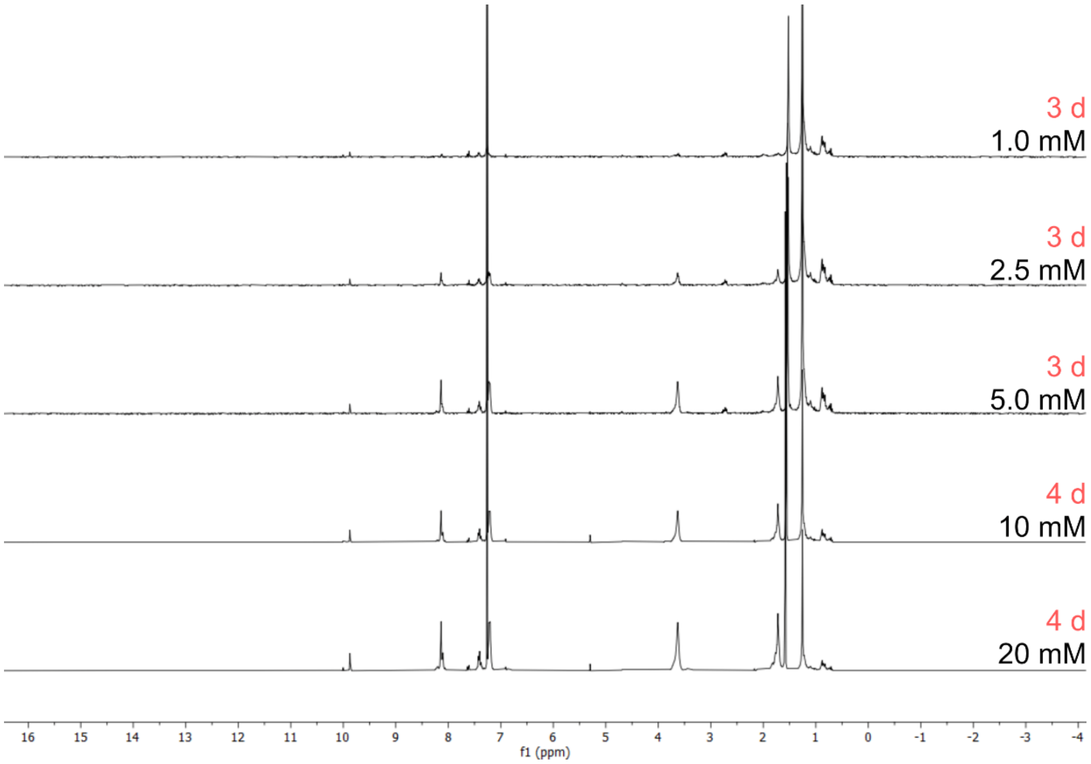

Figure S29: Comparison of ^{1}H NMR spectra ($CDCl_3$, 300 MHz) of *E*-**A** and **B** at different concentrations after stirring under irradiation with red light.

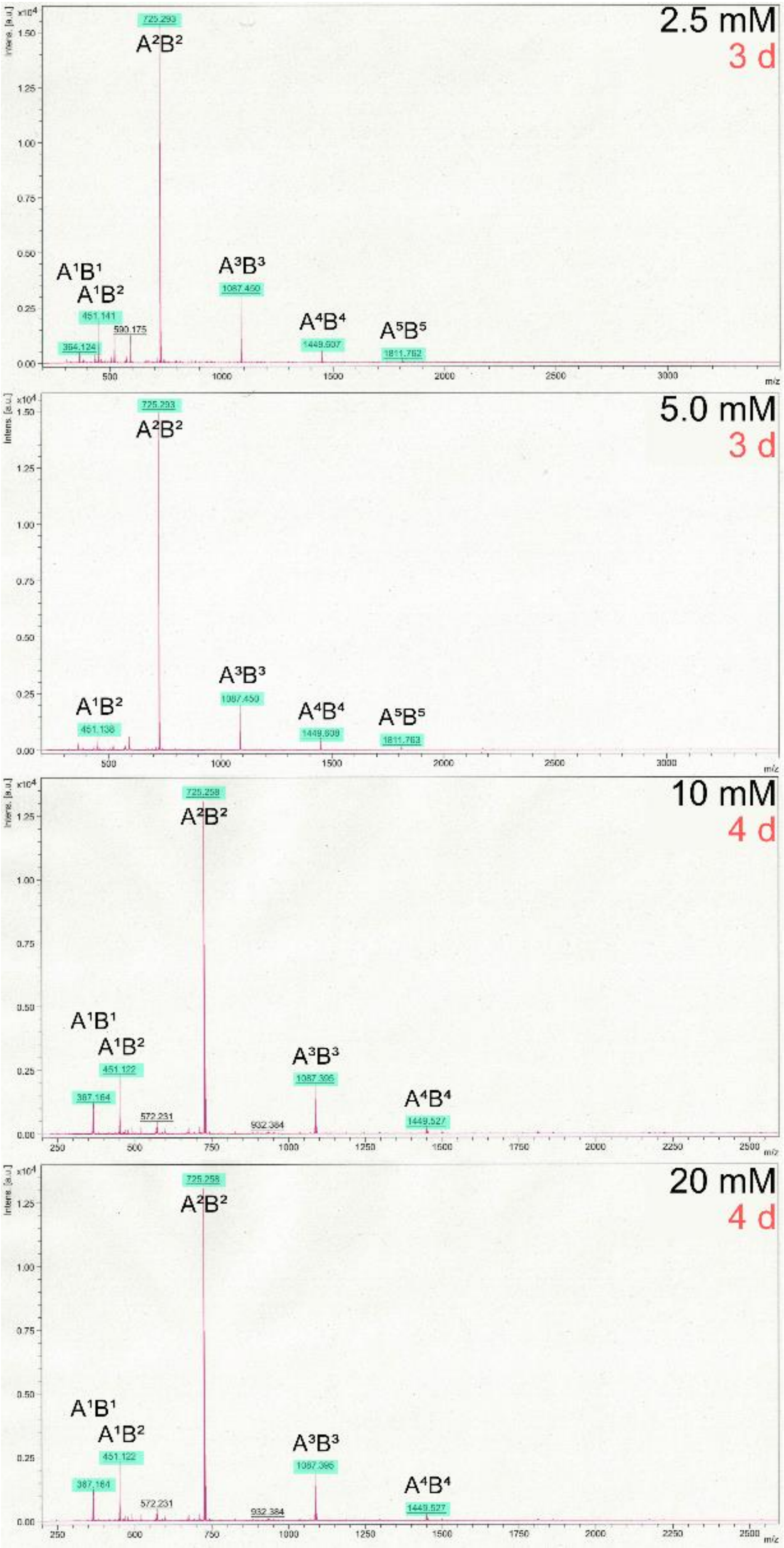

Figure S30: Comparison of MALDI-mass spectra of the reaction of *E*-**A** and **B** at different concentrations under irradiation with red light (660 nm). Peaks that can be attributed to $\mathbf{A^nB^n}$ compounds are highlighted in turquoise.

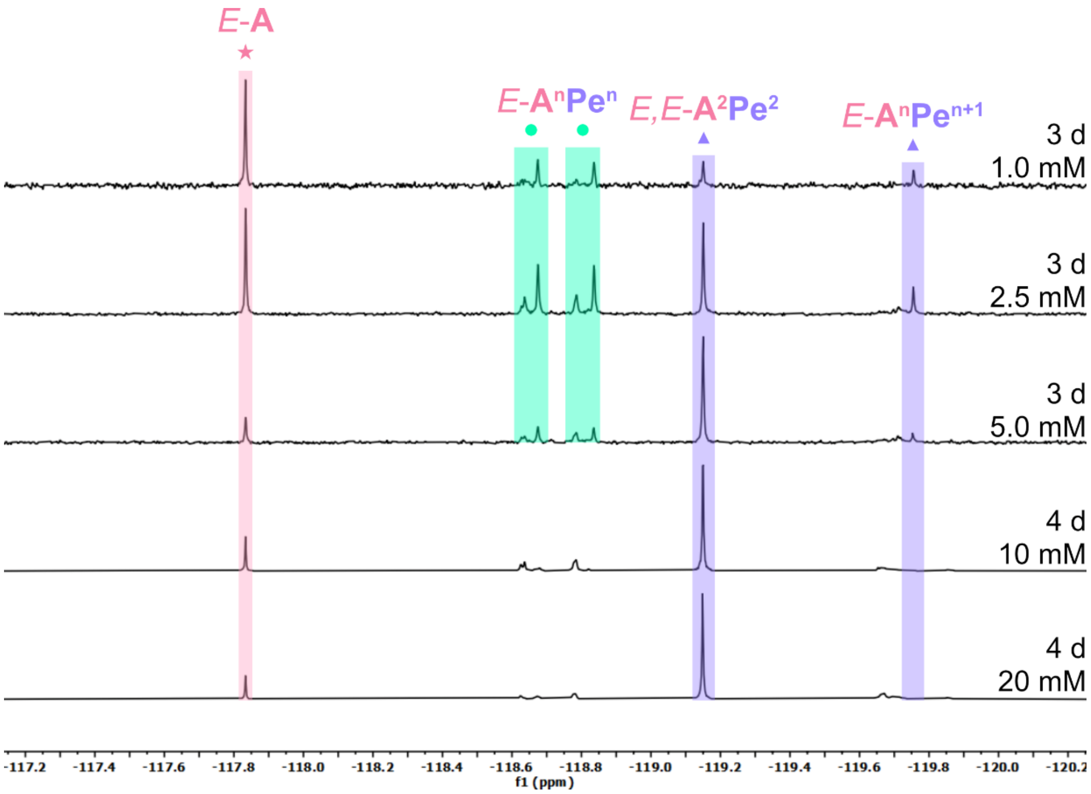

Figure S31: Comparison of $^{19}F\{^1H\}$ NMR spectra ($CDCl_3$, 282 MHz) of *E*-**A** and **Pe** at different concentrations after stirring in the dark.

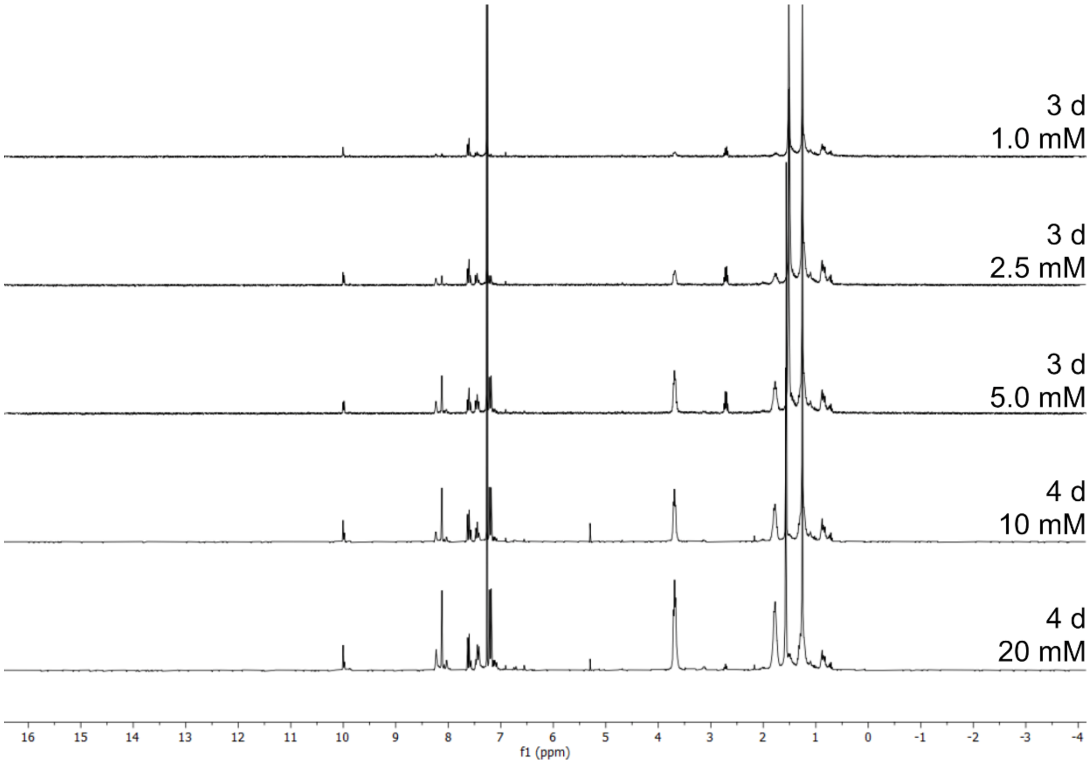

Figure S32: Comparison of ^1H NMR spectra ($CDCl_3$, 300 MHz) of *E*-**A** and **Pe** at different concentrations after stirring in the dark.

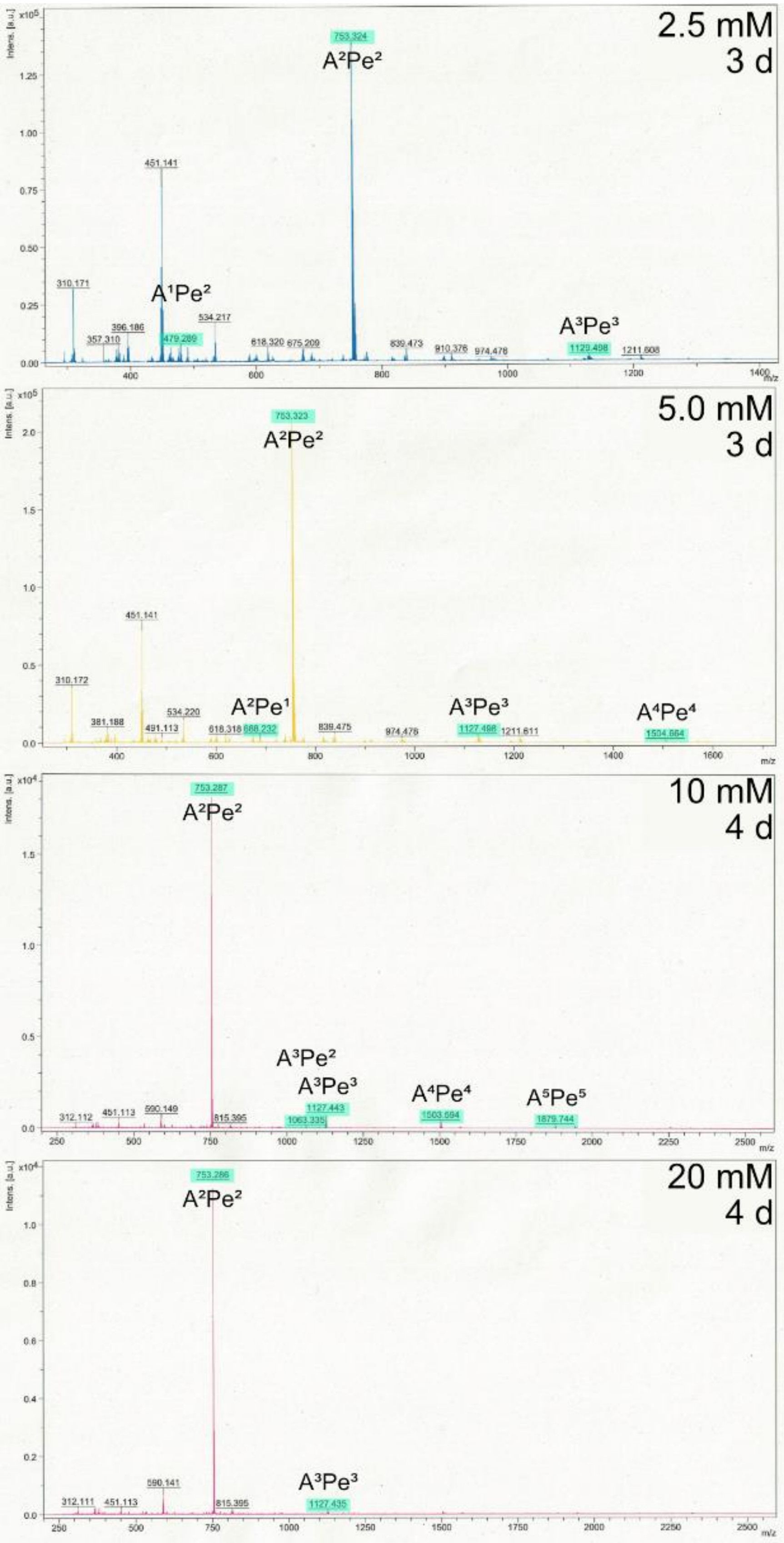

Figure S33: Comparison of MALDI-mass spectra of the reaction of *E*-**A** and **Pe** at different concentrations under irradiation in the dark. Peaks that can be attributed to **A^{n}Pe^n** compounds are highlighted in turquoise.

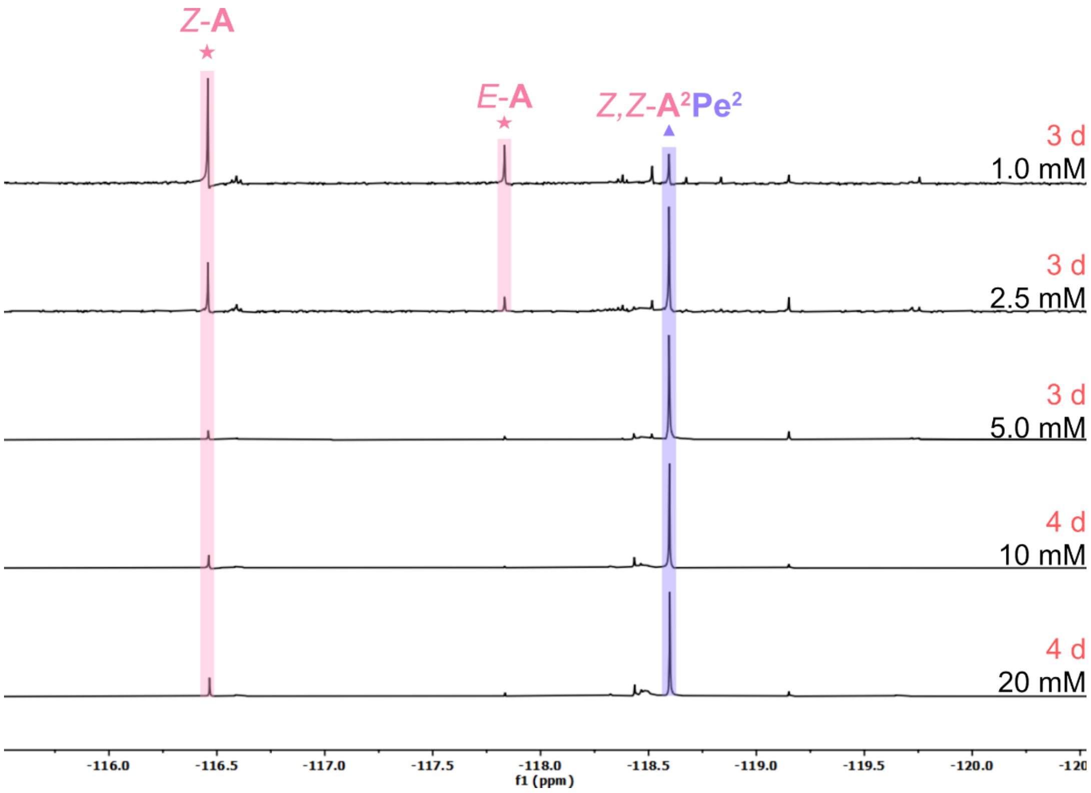

Figure S34: Comparison of ^{19}F{^{1}H} NMR spectra ($CDCl_3$, 282 MHz) of *E*-**A** and **Pe** at different concentrations after stirring under irradiation with red light.

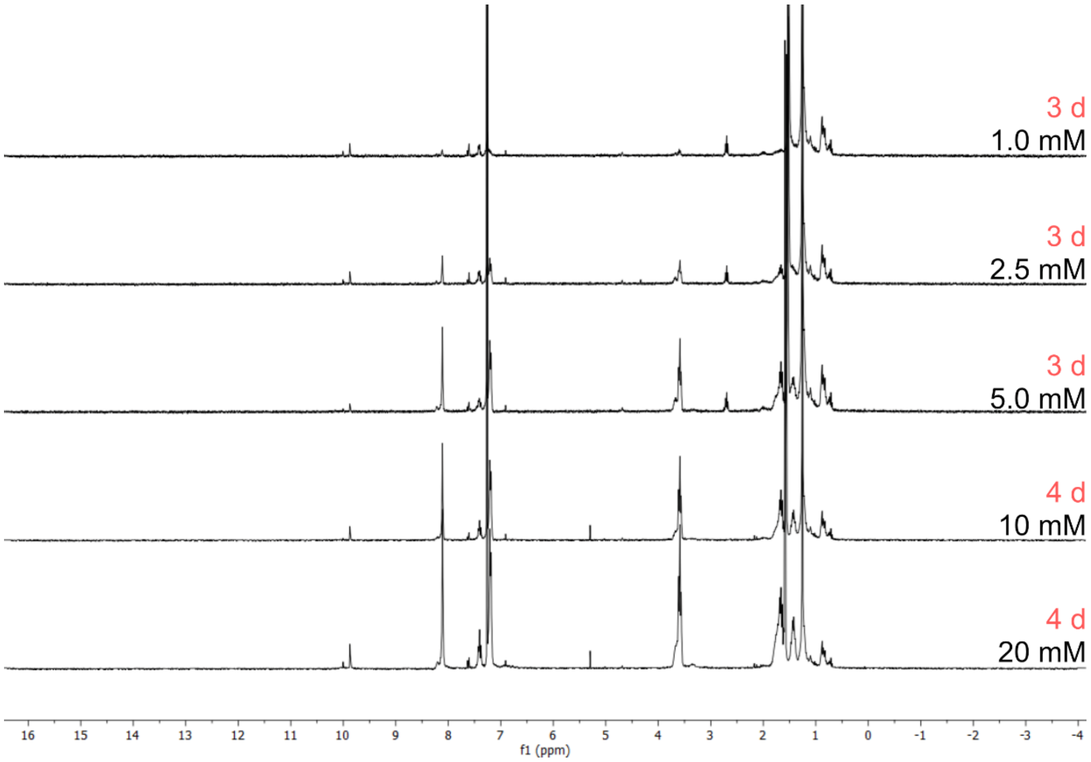

Figure S35: Comparison of ^{1}H NMR spectra ($CDCl_3$, 300 MHz) of *E*-**A** and **Pe** at different concentrations after stirring under irradiation with red light.

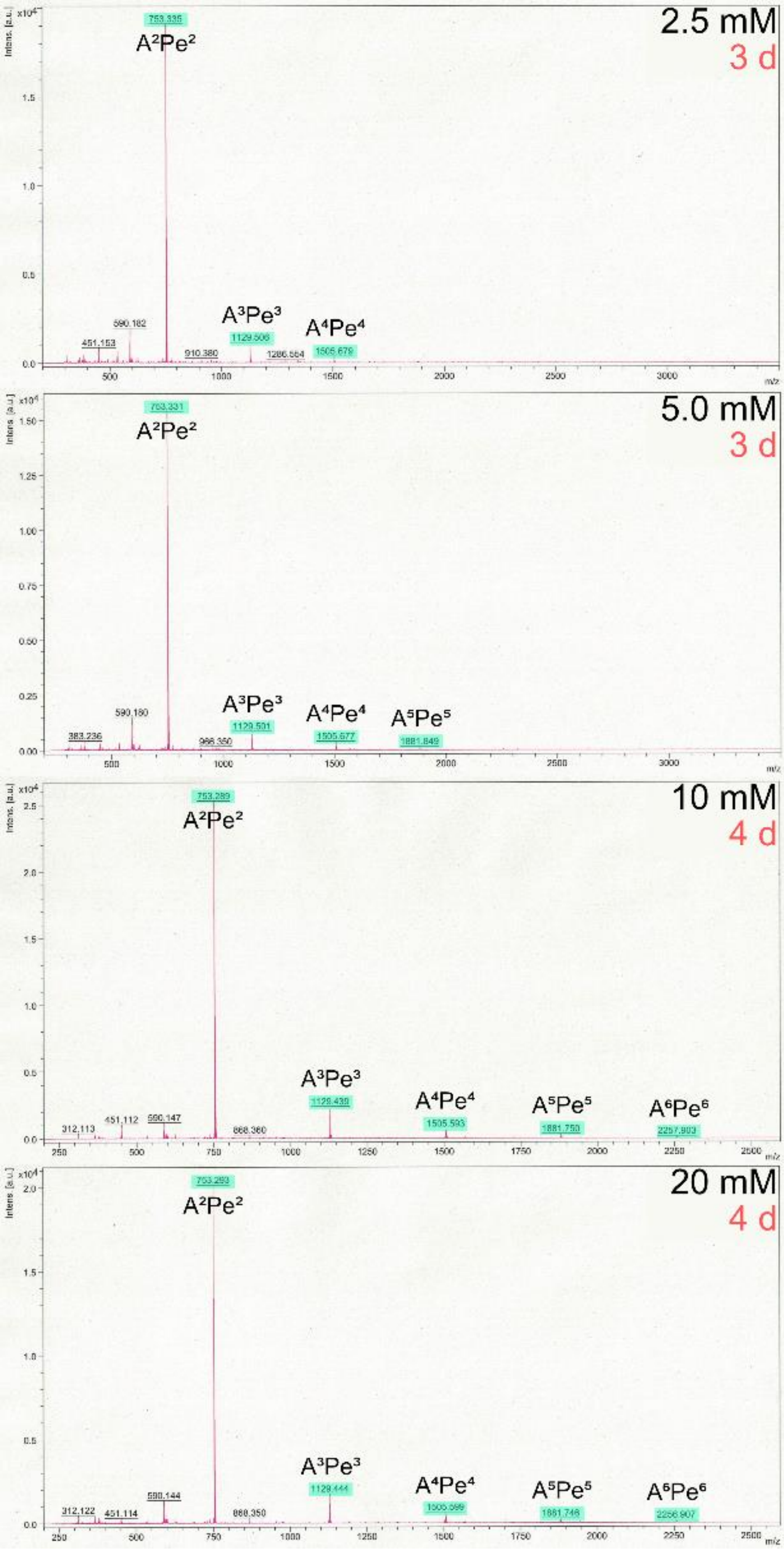

Figure S36: Comparison of MALDI-mass spectra of the reaction of *E*-**A** and **Pe** at different concentrations under irradiation with red light (660 nm). Peaks that can be attributed to $\mathbf{A^nPe^n}$ compounds are highlighted in turquoise.

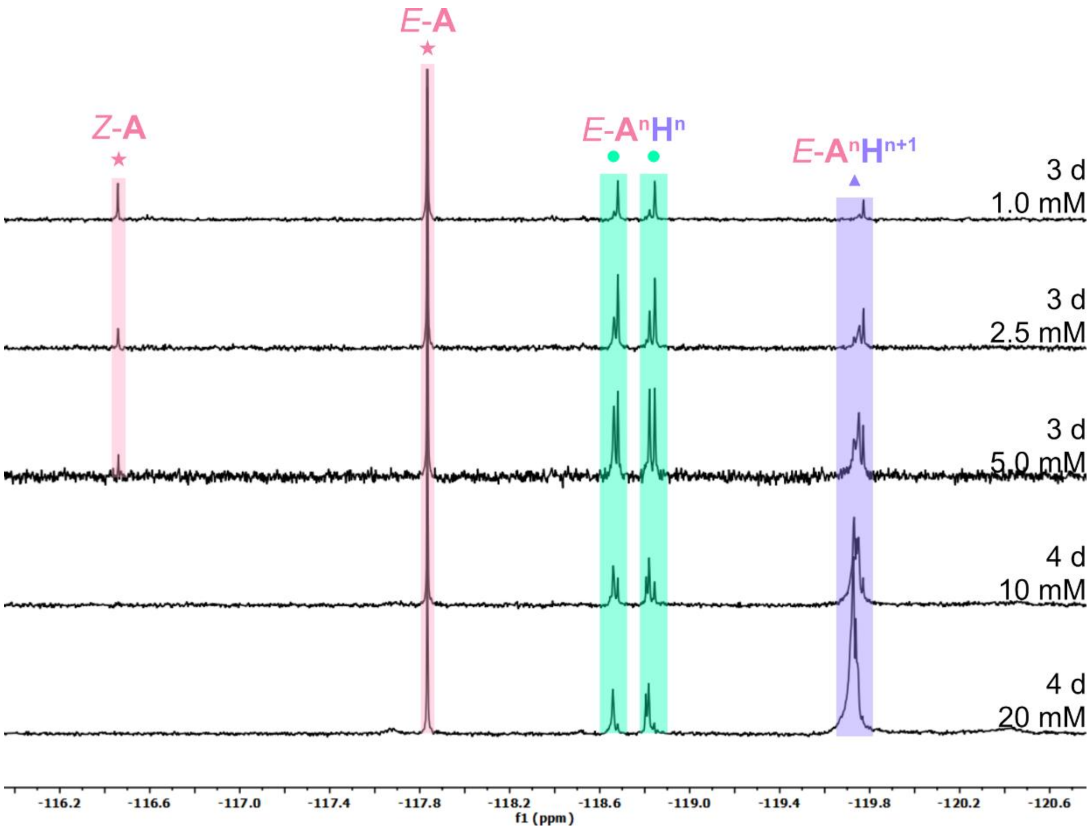

Figure S37: Comparison of $^{19}F\{^{1}H\}$ NMR spectra ($CDCl_3$, 282 MHz) of *E*-**A** and **H** at different concentrations after stirring in the dark.

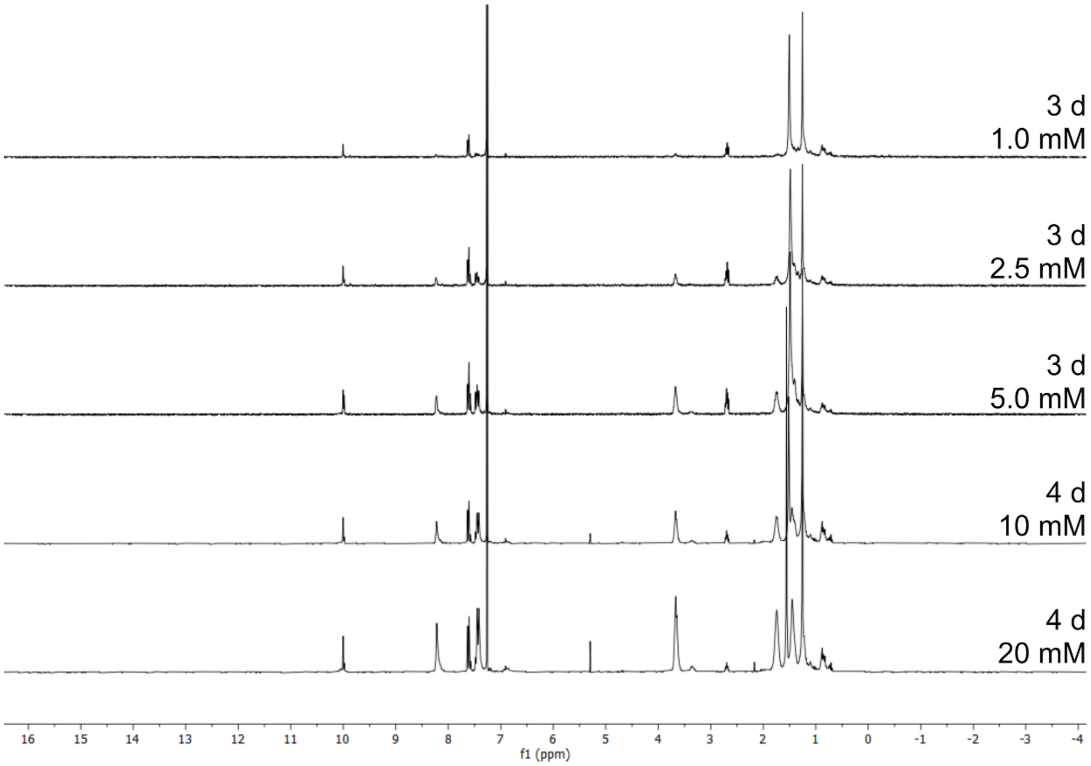

Figure S38: Comparison of ^{1}H NMR spectra ($CDCl_3$, 300 MHz) of *E*-**A** and **H** at different concentrations after stirring in the dark.

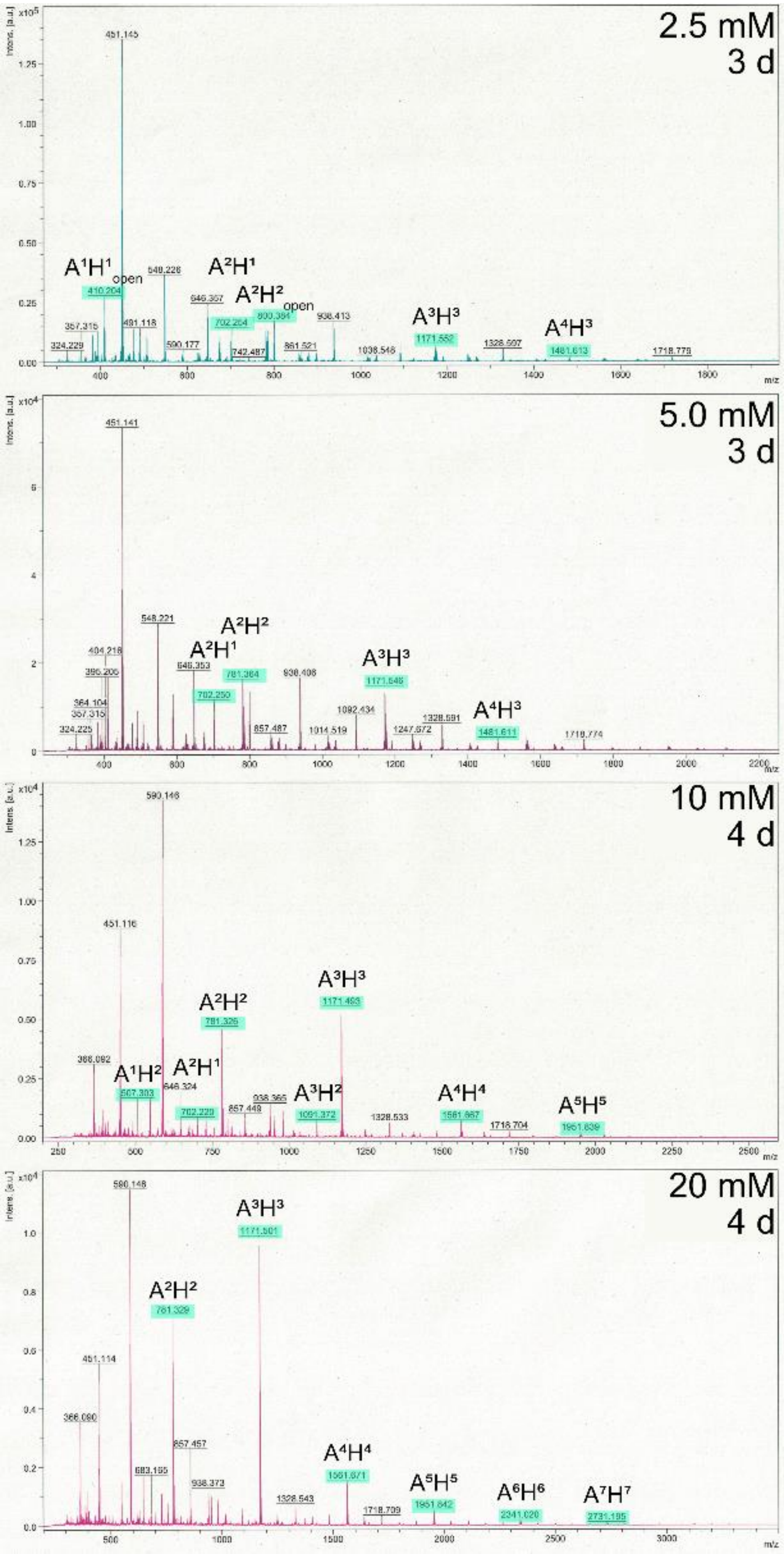

Figure S39: Comparison of MALDI-mass spectra of the reaction of *E*-**A** and **H** at different concentrations in the dark. Peaks that can be attributed to $\mathbf{A^nH^n}$ compounds are highlighted in turquoise.

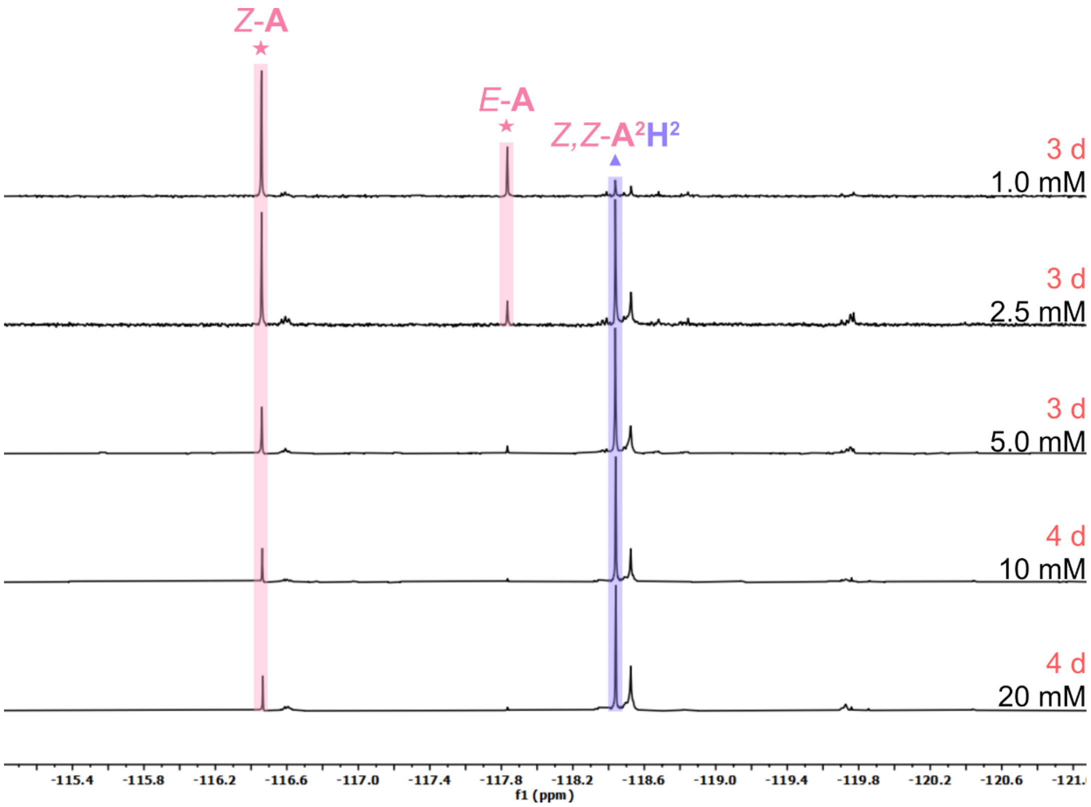

Figure S40: Comparison of ${}^{19}F\{{}^{1}H\}$ NMR spectra ($CDCl_3$, 282 MHz) of *E*-**A** and **H** at different concentrations after stirring under irradiation with red light.

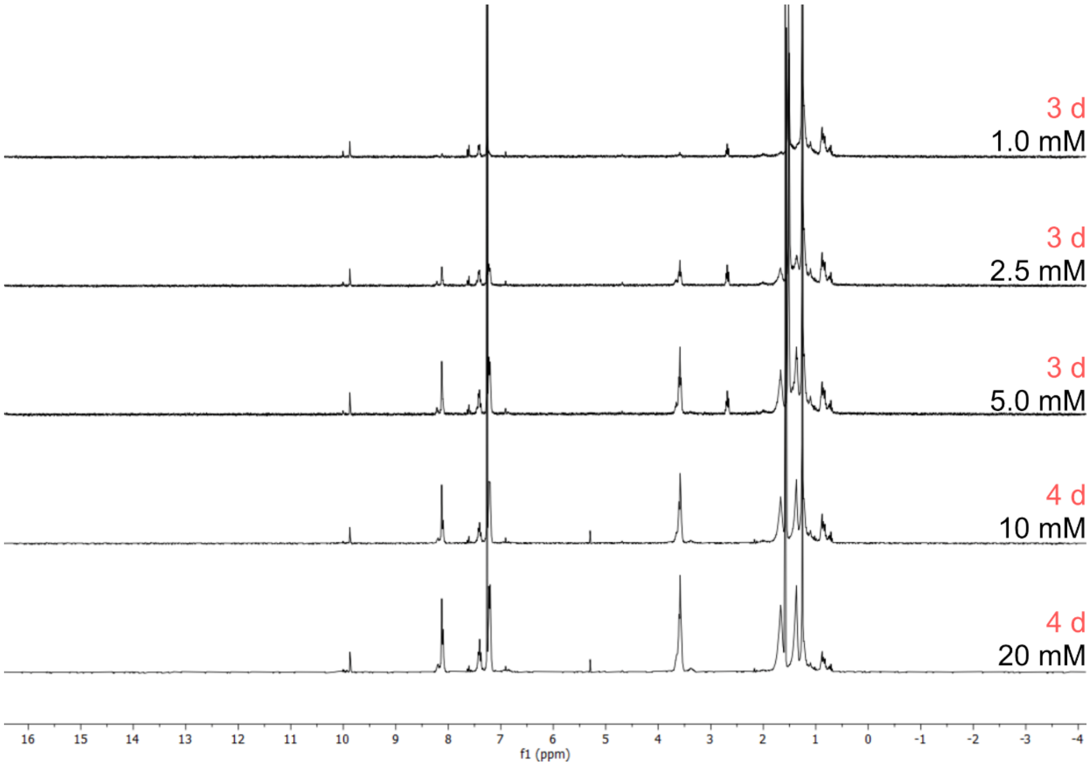

Figure S41: Comparison of ${}^{1}H$ NMR spectra ($CDCl_3$, 300 MHz) of *E*-**A** and **H** at different concentrations after stirring under irradiation with red light.

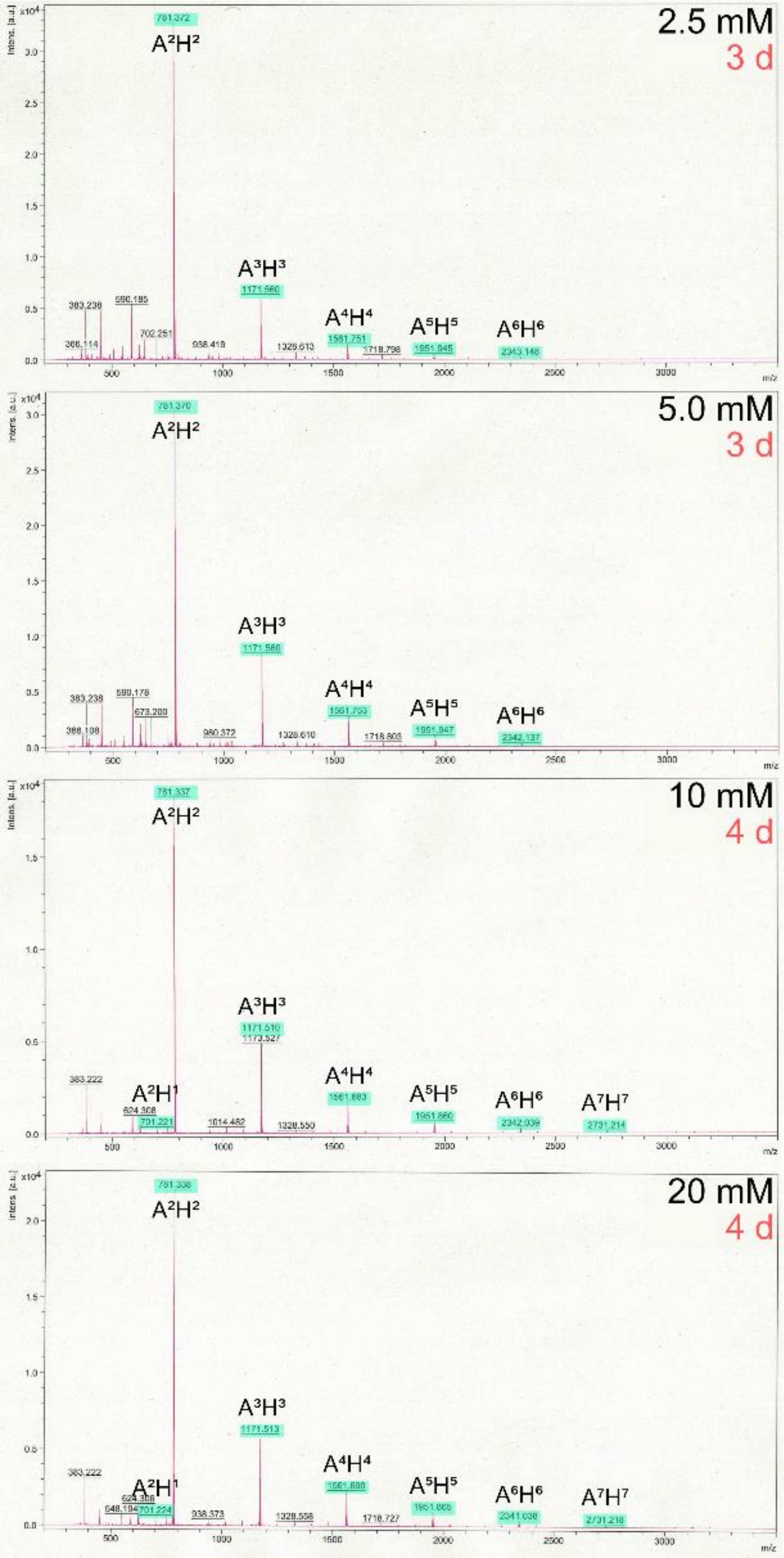

Figure S42: Comparison of MALDI-mass spectra of the reaction of *E*-**A** and **H** at different concentrations under irradiation with red light (660 nm). Peaks that can be attributed to $\mathbf{A^nH^n}$ compounds are highlighted in turquoise.

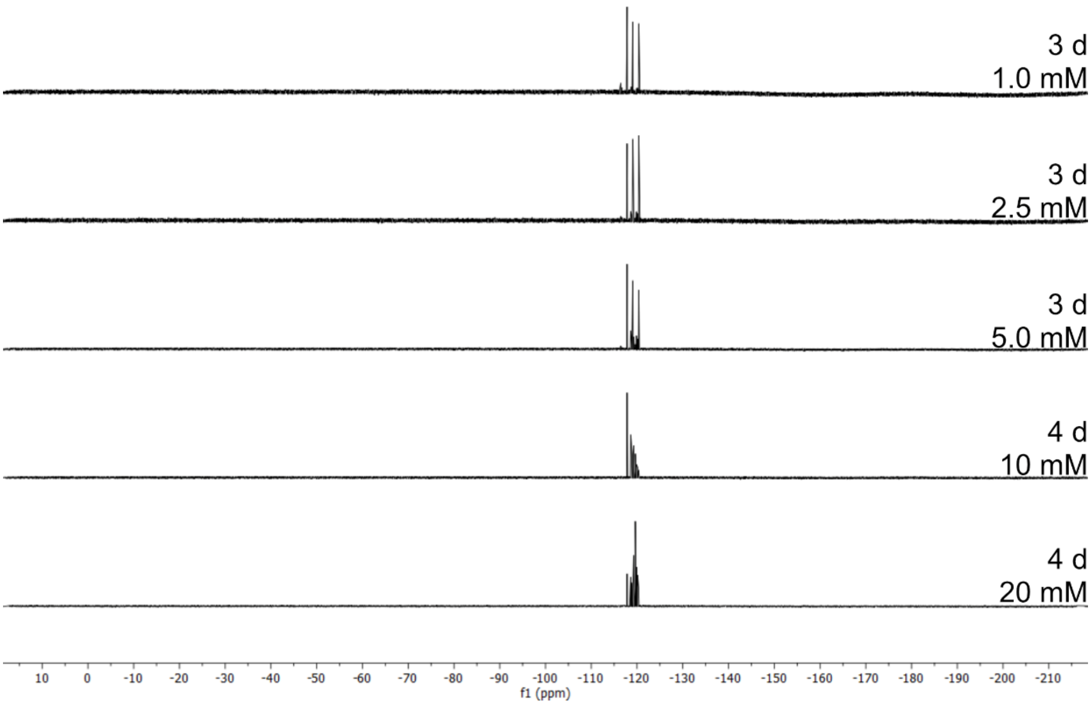

Figure S43: Comparison of $^{19}F\{^{1}H\}$ NMR spectra ($CDCl_3$, 282 MHz) of *E*-**A** and **Pr** at different concentrations after stirring in the dark.

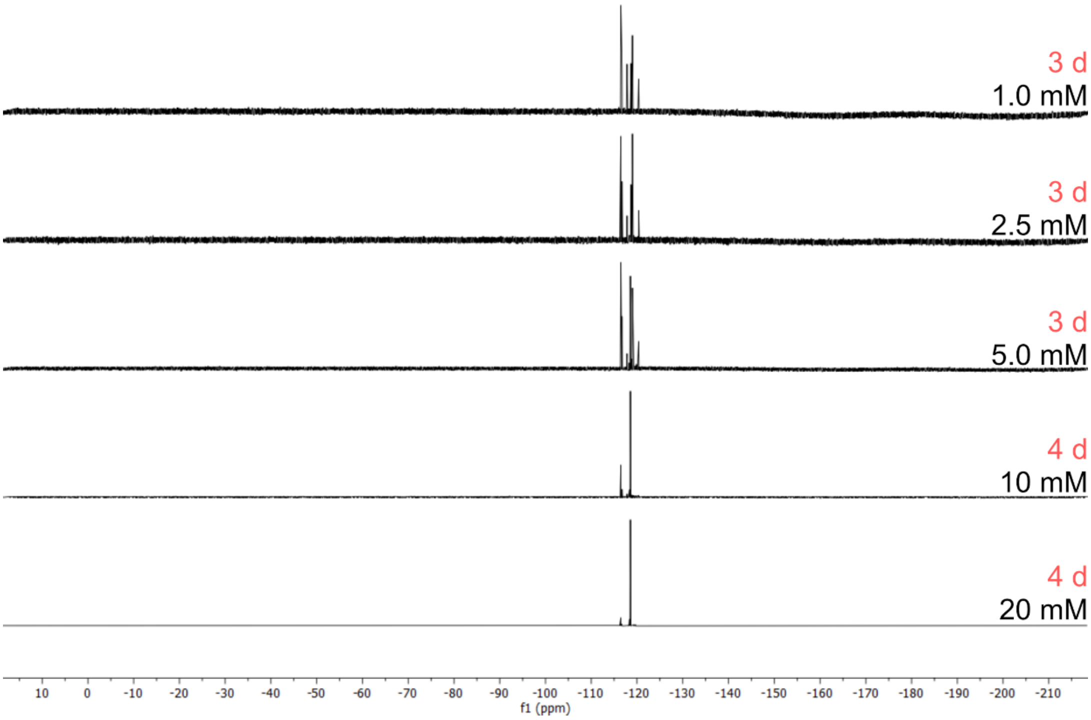

Figure S44: Comparison of $^{19}F\{^{1}H\}$ NMR spectra ($CDCl_3$, 282 MHz) of *E*-**A** and **Pr** at different concentrations after stirring under irradiation with red light (660 nm).

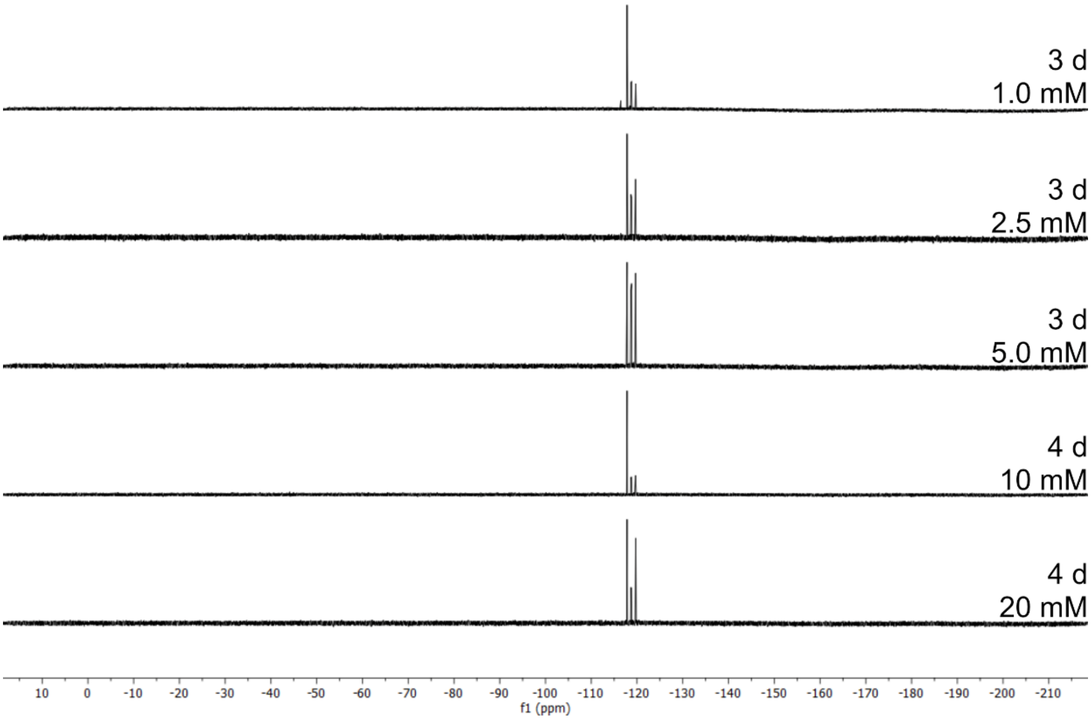

Figure S45: Comparison of $^{19}F\{^{1}H\}$ NMR spectra ($CDCl_3$, 282 MHz) of *E*-**A** and **B** at different concentrations after stirring in the dark.

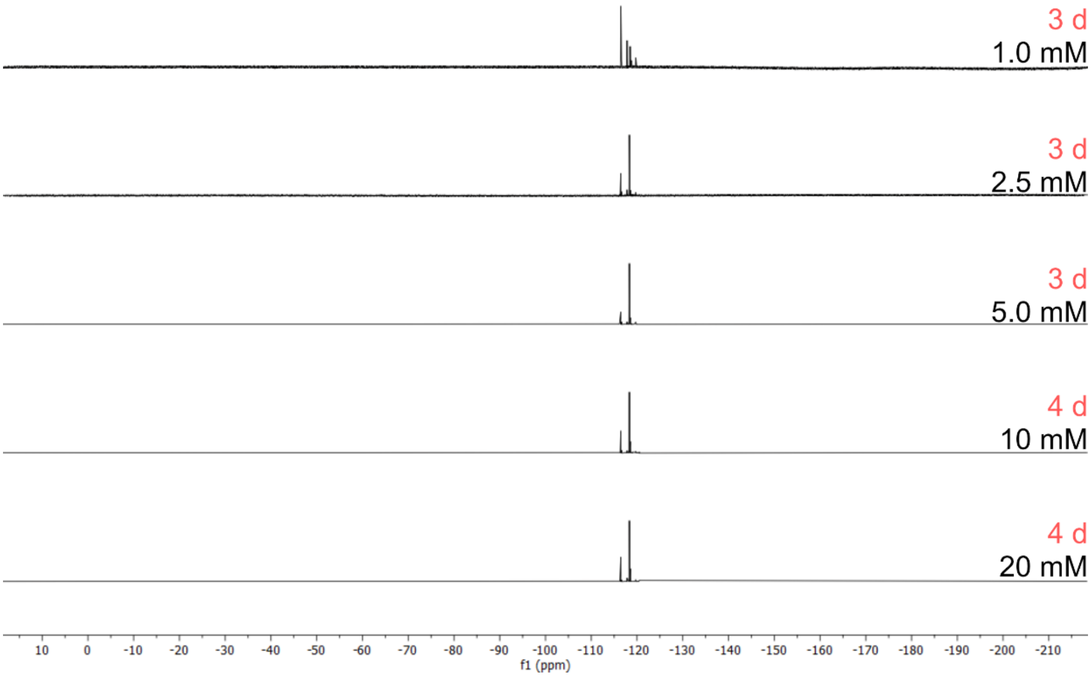

Figure S46: Comparison of $^{19}F\{^{1}H\}$ NMR spectra ($CDCl_3$, 282 MHz) of *E*-**A** and **B** at different concentrations after stirring under irradiation with red light (660 nm).

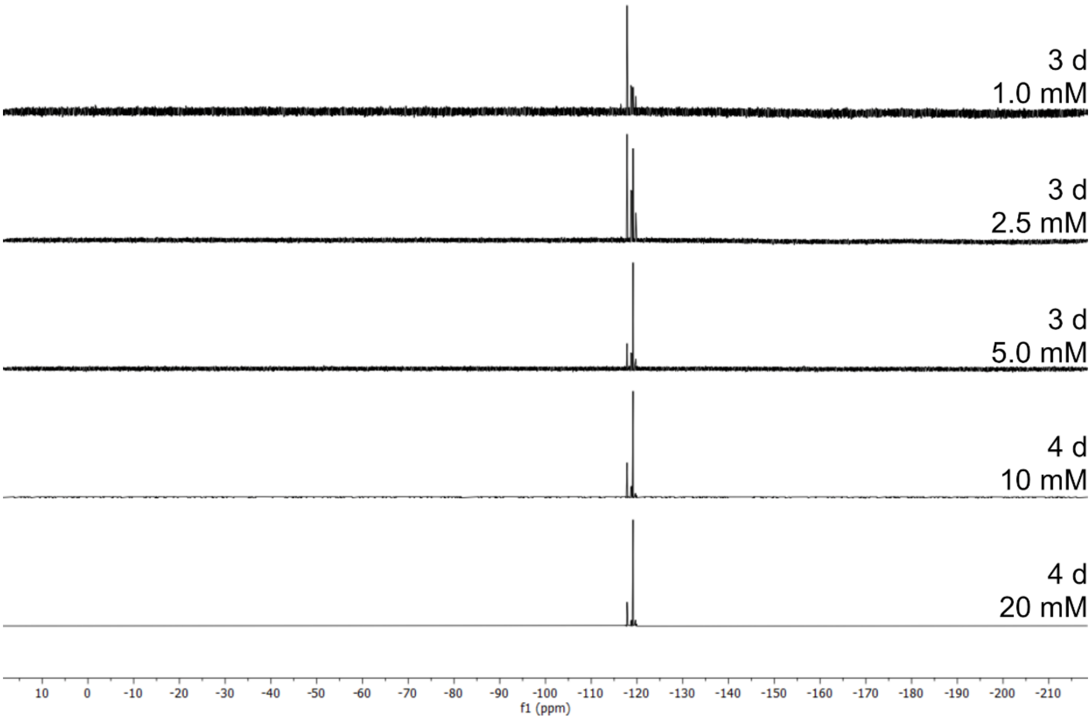

Figure S47: Comparison of $^{19}F\{^1H\}$ NMR spectra ($CDCl_3$, 282 MHz) of *E*-**A** and **Pe** at different concentrations after stirring in the dark.

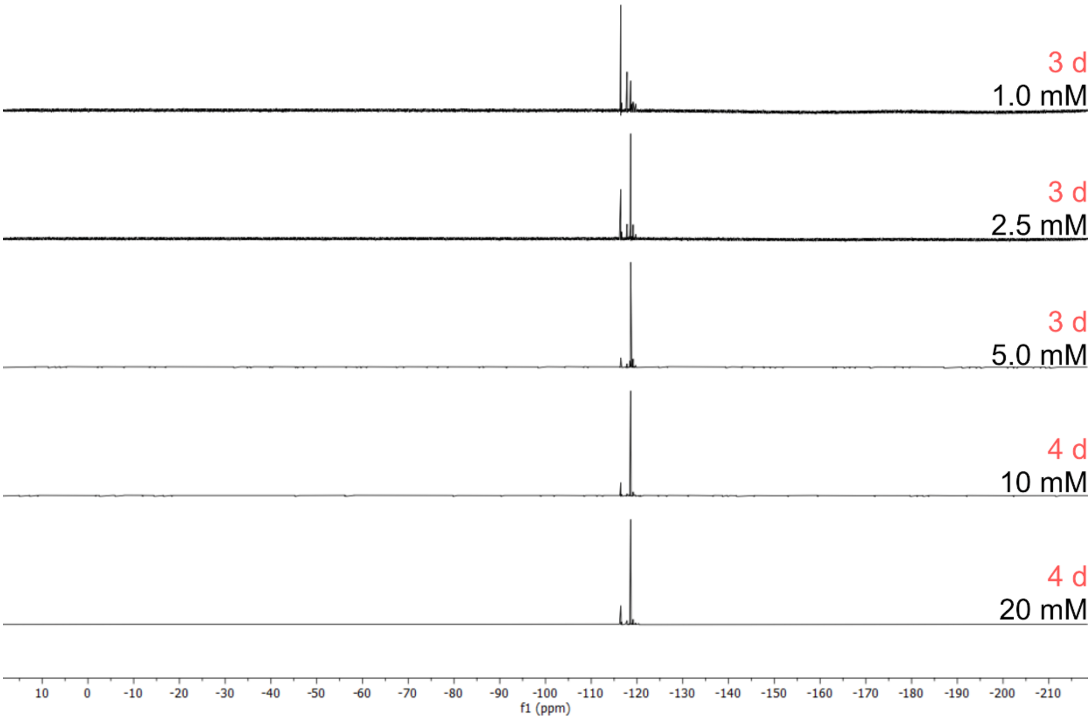

Figure S48: Comparison of $^{19}F\{^1H\}$ NMR spectra ($CDCl_3$, 282 MHz) of *E*-**A** and **Pe** at different concentrations after stirring under irradiation with red light (660 nm).

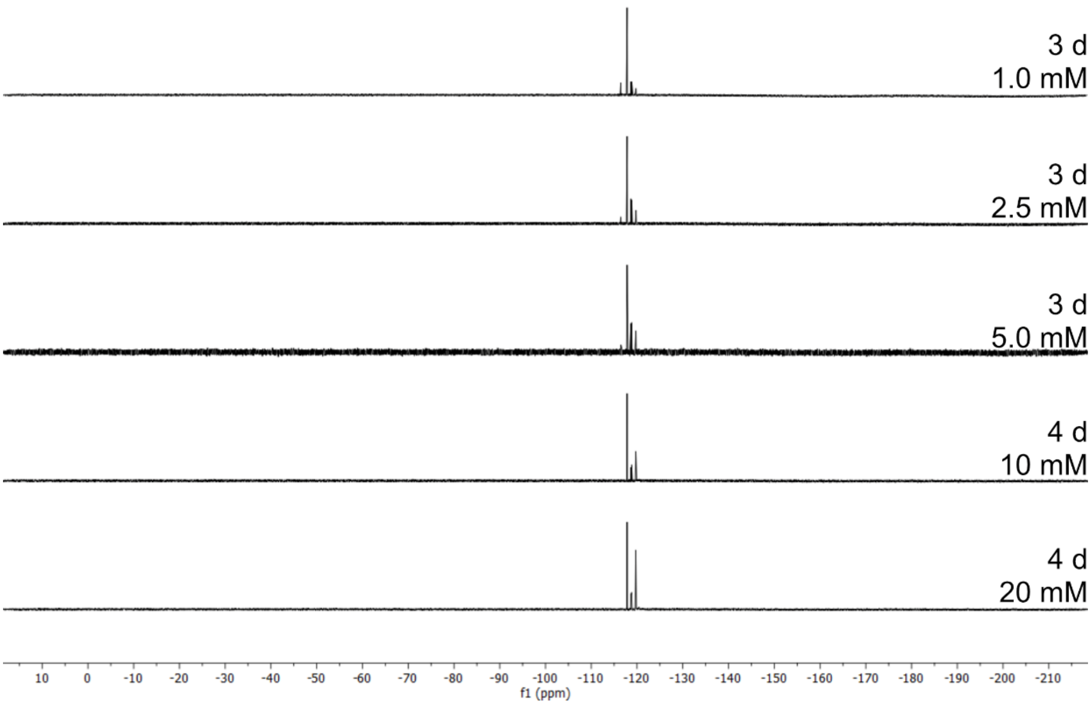

Figure S49: Comparison of $^{19}F\{^{1}H\}$ NMR spectra ($CDCl_3$, 282 MHz) of *E*-**A** and **H** at different concentrations after stirring in the dark.

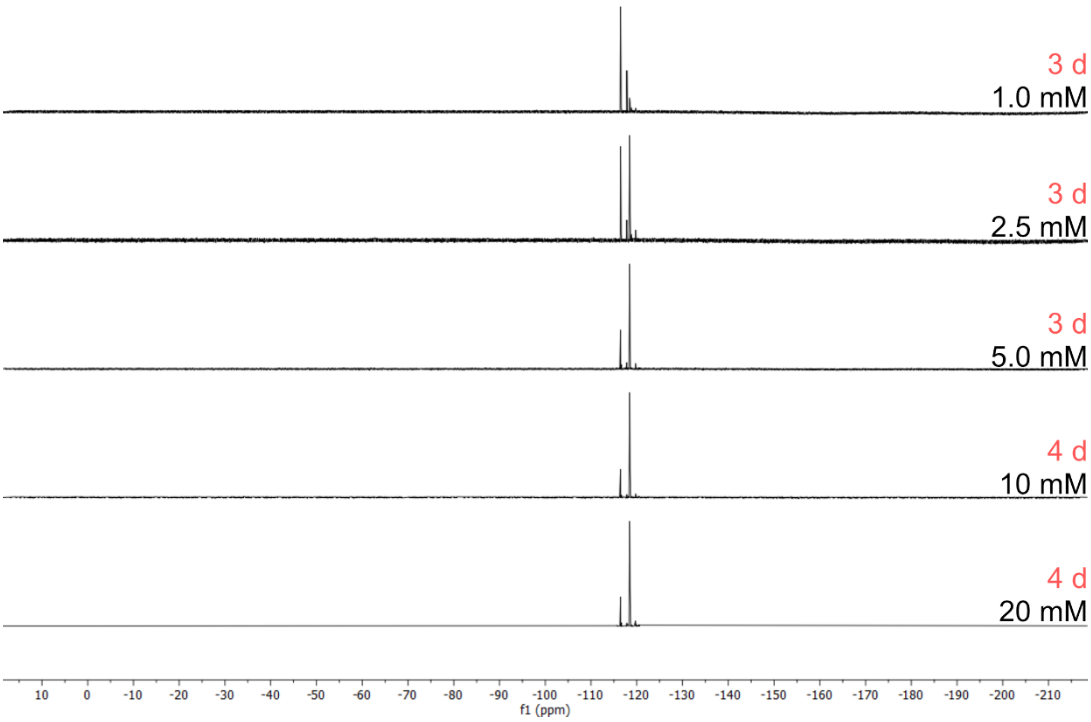

Figure S50: Comparison of $^{19}F\{^{1}H\}$ NMR spectra ($CDCl_3$, 282 MHz) of *E*-**A** and **H** at different concentrations after stirring under irradiation with red light (660 nm).

V. ^{19}F DOSY NMR measurements

^{19}F-diffusion NMR experiments have been performed on a BRUKER Avance *NEO* 600 FT NMR spectrometer, operating at a ^{1}H resonance frequency of 600.13 MHz at 293 K. The instrument was equipped with a 5 mm BBO Prodigy cryoprobe exhibiting a z-gradient coil delivering a maximum gradient strength of 6.57 G mm^{-1} at 10 A. Diffusion data has been recorded using the *dstebpgp3s* pulse sequence delivered by the manufacturer. Diffusion coefficients D have been corrected according to the diffusion coefficient of H_2O (2.299 • 10^{-9} m^2 s^{-1} at 298 K) reported in the literature.[5,6] The corresponding proportional factor $D_{H2O,lit.}/D_{H2O,est.}$ of 0.98 was determined on a sample of acetone-d_6 equipped with a capillary containing H_2O. The temperature unit of the instrument was calibrated according to the manual of the manufacturer of the instrument using the temperature dependence of the proton chemical shift difference of methanol. To obtain stable temperature conditions, the sample was kept within the magnet for at least one hour at the respective temperature prior to data collection. ^{19}F-diffusion data were recorded with 32k data points and a spectral width of 8620 Hz using a relaxation delay of 5 s. The diffusion delay time (big Delta, Δ) was set to 50 ms. The gradient duration time (little delta, δ/2) has been adjusted to values between 1300 and 1800 µs. The gradient strength within the diffusion experiments was incremented linearly using 16 steps. The diffusion data has been analysed with the T_1/T_2 module which is part of the BRUKER TopSpin® software package. The standard deviation of the experimentally determined gradient strength-dependent signal intensities to the fitted decay function was ≤ 7.9 • 10^{-3}. DOSY plots presenting uncorrected diffusion constants in the F1 dimension have been prepared with TopSpin. The solvodynamic radii r_s have been calculated by the Stokes-Einstein equation (equation (1)) and the solvodynamic volumes V_s have been calculated by the equation for a spherical volume (equation (2)),

$$D = \frac{k_B T}{6\pi\eta r_s} \tag{1}$$

$$V_s = \frac{4}{3}\pi r_s^3 \tag{2}$$

where $k_B = 1.381 \cdot 10^{-23}\, J \cdot \mathrm{K}^{-1}$ [7] is the Boltzmann constant, T is the absolute temperature, and $\eta_{CHCl_3}(293.15\ K) = 0.573 \cdot 10^{-3}\ Pa \cdot \mathrm{s}$.[8] The samples were prepared as described in Table S2.

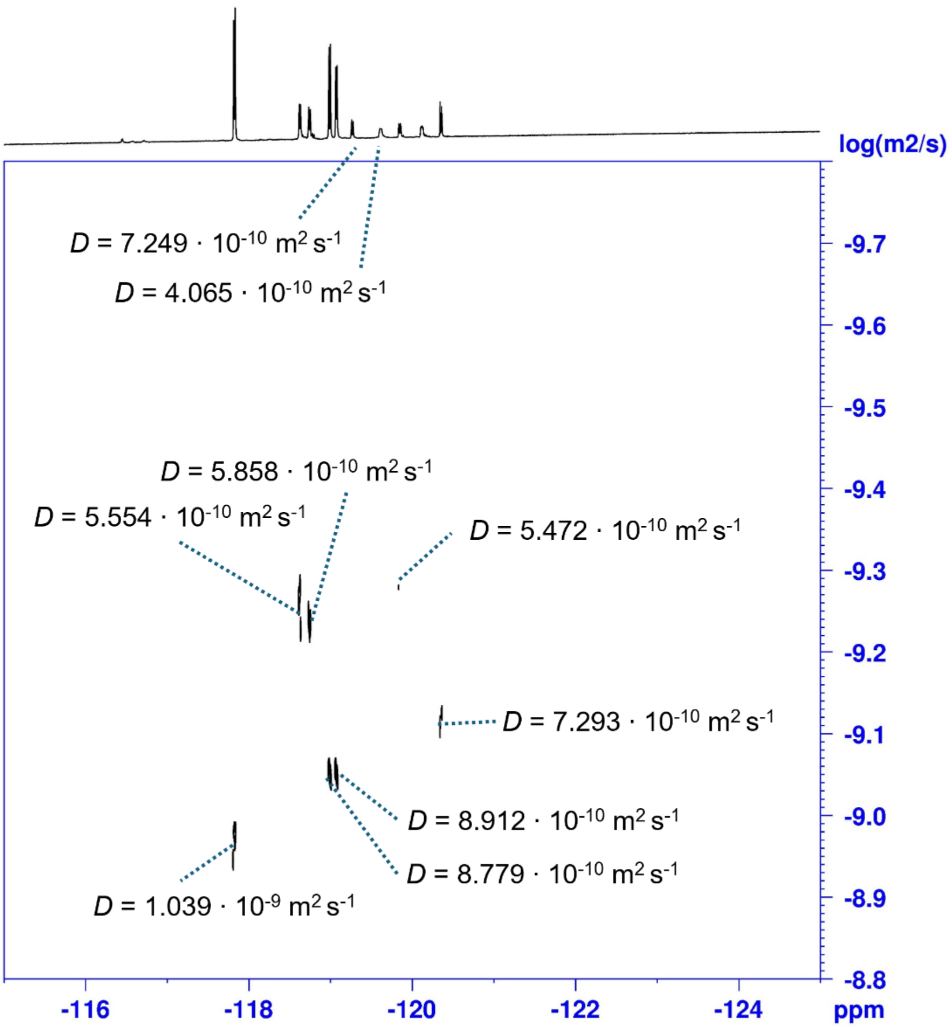

Figure S51: ^{19}F DOSY NMR spectrum ($CDCl_3$, 565 MHz) of a solution of *E*-**A** and **Pr** in the dark (refer to Table S2) with uncorrected diffusion coefficients *D*.

Table S4: Integral range, uncorrected and corrected diffusion coefficients *D*, solvodynamic radii r_s and solvodynamic volumes V_s (both calculated based on the corrected diffusion coefficients $D_{corrected}$) extracted from ^{19}F DOSY NMR spectra of a solution of *E*-**A** and **Pr** in the dark (refer to Table S2).

	integral range [ppm]		*D* [m^2 s^{-1}]	$D_{corrected}$ [m^2 s^{-1}]	r_s [Å]	V_s [Å^3]
E-**A**	-117.77	-117.888	$1.039 \cdot 10^{-9}$	$1.018 \cdot 10^{-9}$	3.679	208.6
	-118.578	-118.659	$5.554 \cdot 10^{-10}$	$5.443 \cdot 10^{-10}$	6.883	1366
	-118.705	-118.761	$5.858 \cdot 10^{-10}$	$5.741 \cdot 10^{-10}$	6.526	1164
$\mathbf{A^nPr^n}$	-118.942	-119.028	$8.779 \cdot 10^{-10}$	$8.603 \cdot 10^{-10}$	4.354	345.9
$\mathbf{A^nPr^n}$	-119.028	-119.112	$8.912 \cdot 10^{-10}$	$8.734 \cdot 10^{-10}$	4.289	330.6
	-119.236	-119.289	$7.249 \cdot 10^{-10}$	$7.104 \cdot 10^{-10}$	5.274	614.3
	-119.55	-119.662	$4.065 \cdot 10^{-10}$	$3.984 \cdot 10^{-10}$	9.404	3484
	-119.805	-119.885	$5.472 \cdot 10^{-10}$	$5.363 \cdot 10^{-10}$	6.986	1428
$\mathbf{A^nPr^{n+1}}$	-120.305	-120.389	$7.293 \cdot 10^{-10}$	$7.147 \cdot 10^{-10}$	5.242	603.3

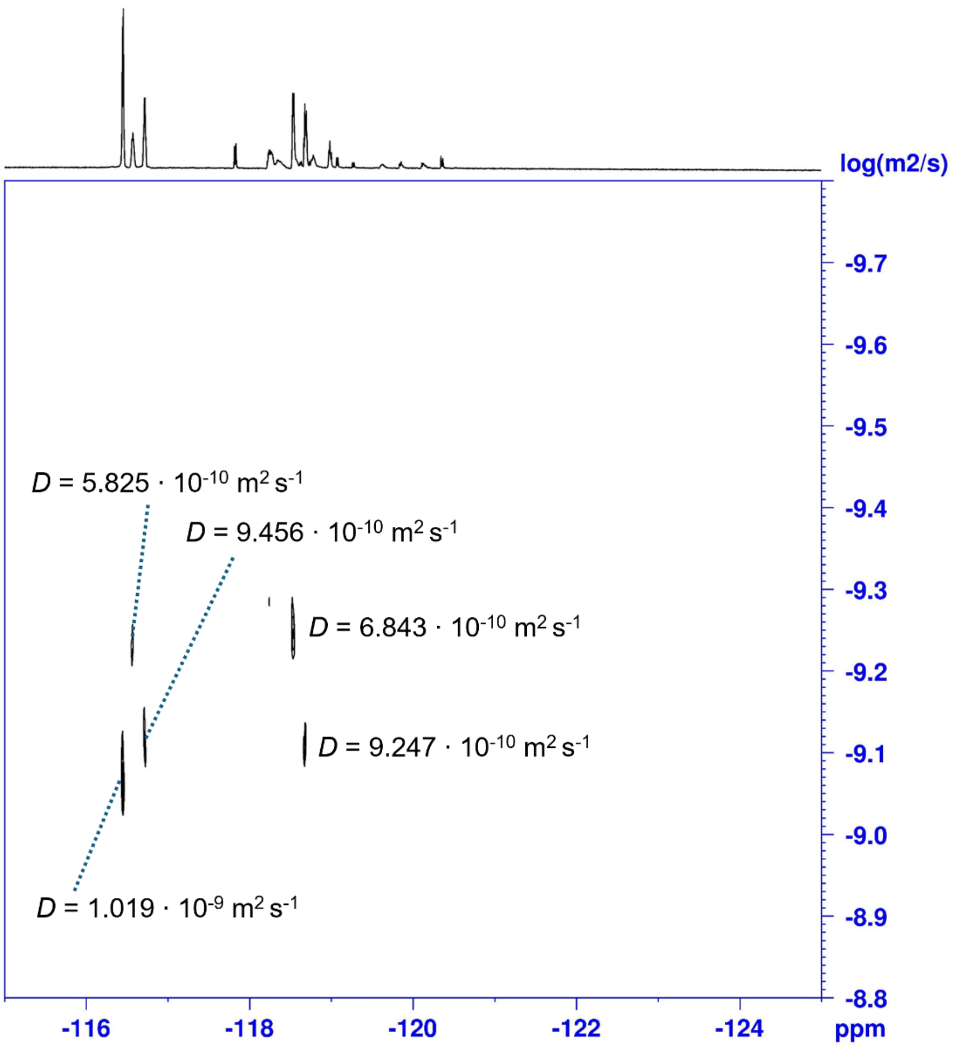

Figure S52: ^{19}F DOSY NMR spectrum ($CDCl_3$, 565 MHz) of a solution of *Z*-**A**-enriched solution with **Pr** after continuous irradiation with light of a wavelength of 660 nm (refer to Table S2) with uncorrected diffusion coefficients *D*.

Table S5: Integral range, uncorrected and corrected diffusion coefficients *D*, solvodynamic radii r_s and solvodynamic volumes V_s (both calculated based on the corrected diffusion coefficients $D_{corrected}$) extracted from ^{19}F DOSY NMR spectra of a *Z*-**A**-enriched solution with **Pr** after continuous irradiation with light of a wavelength of 660 nm (refer to Table S2).

	integral range [ppm]		D [$m^2\ s^{-1}$]	$D_{corrected}$ [$m^2\ s^{-1}$]	r_s [Å]	V_s [Å^3]
Z-**A**	-116.385	-116.497	$1.040 \cdot 10^{-9}$	$1.019 \cdot 10^{-9}$	3.676	208.0
	-116.488	-116.599	$5.944 \cdot 10^{-10}$	$5.825 \cdot 10^{-10}$	6.431	1114
	-116.658	-116.777	$9.456 \cdot 10^{-10}$	$9.267 \cdot 10^{-10}$	4.043	276.8
Z,*Z*-$\mathbf{A^2Pr^2}$	-118.464	-118.571	$6.843 \cdot 10^{-10}$	$6.706 \cdot 10^{-10}$	5.586	730.3
	-118.629	-118.728	$9.247 \cdot 10^{-10}$	$9.062 \cdot 10^{-10}$	4.134	296.0

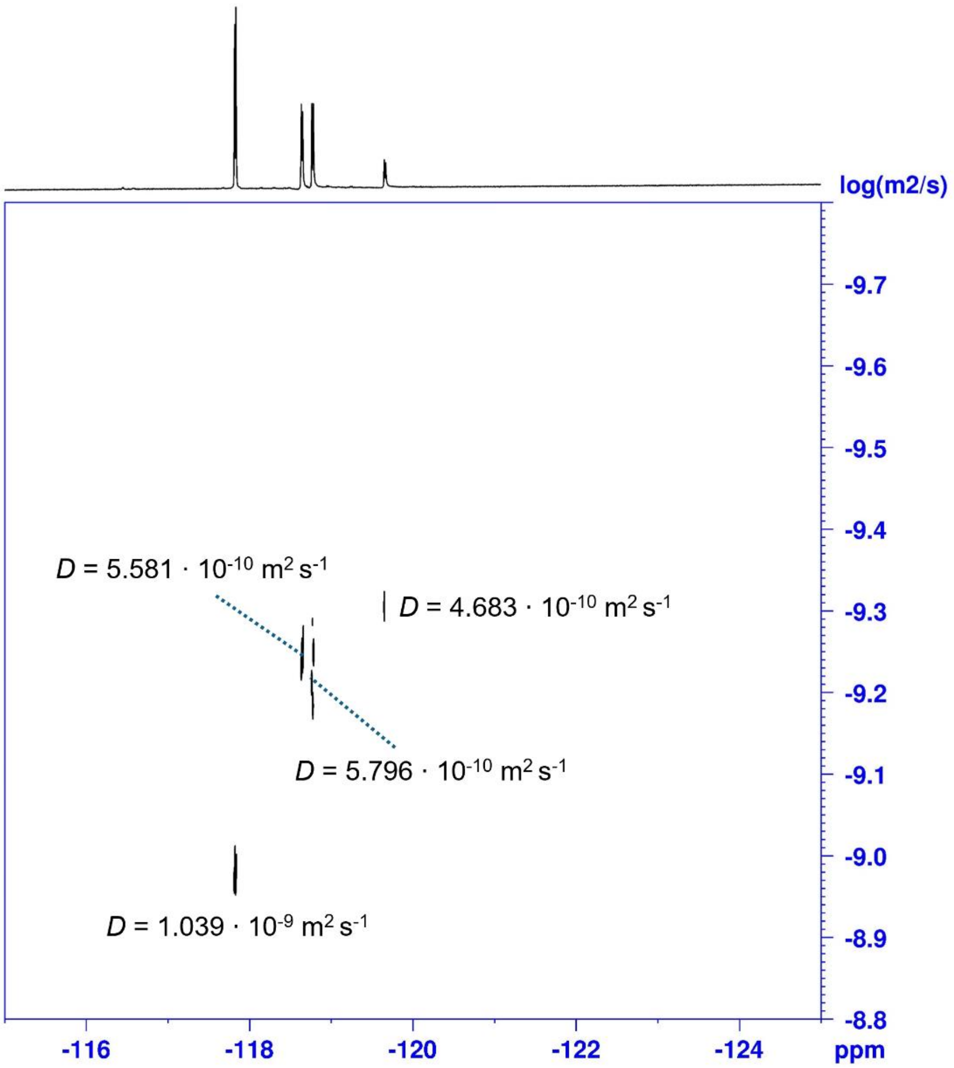

Figure S53: ^{19}F DOSY NMR spectrum ($CDCl_3$, 565 MHz) of a solution of *E*-**A** and **B** in the dark (refer to Table S2) with uncorrected diffusion coefficients *D*.

Table S6: Integral range, uncorrected and corrected diffusion coefficients *D*, solvodynamic radii r_s and solvodynamic volumes V_s (both calculated based on the corrected diffusion coefficients $D_{corrected}$) extracted from ^{19}F DOSY NMR spectra of a solution of *E*-**A** and **B** in the dark (refer to Table S2).

	integral range [ppm]		*D* [m^2 s^{-1}]	$D_{corrected}$ [m^2 s^{-1}]	r_s [Å]	V_s [Å^3]
E-**A**	-117.79	-117.865	$1.039 \cdot 10^{-9}$	$1.018 \cdot 10^{-9}$	3.679	208.6
**AB**	-118.608	-118.673	$5.581 \cdot 10^{-10}$	$5.469 \cdot 10^{-10}$	6.850	1346
**AB**	-118.736	-118.806	$5.796 \cdot 10^{-10}$	$5.680 \cdot 10^{-10}$	6.596	1202
$\mathbf{A^nB^{n+1}}$	-119.63	-119.687	$4.589 \cdot 10^{-10}$	$4.589 \cdot 10^{-10}$	8.163	2279

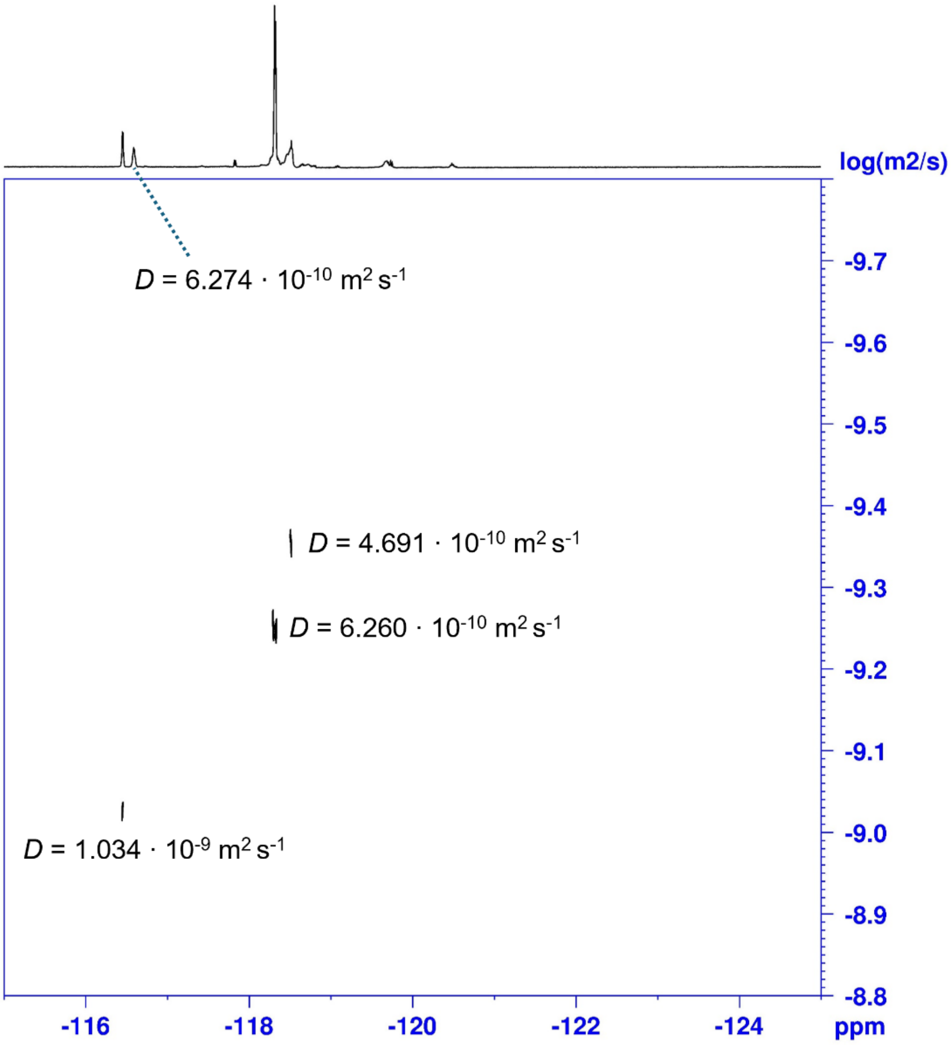

Figure S54: ^{19}F DOSY NMR spectrum ($CDCl_3$, 565 MHz) of a solution of *Z*-**A**-enriched solution with **B** after continuous irradiation with light of a wavelength of 660 nm (refer to Table S2) with uncorrected diffusion coefficients *D*.

Table S7: Integral range, uncorrected and corrected diffusion coefficients *D*, solvodynamic radii r_s and solvodynamic volumes V_s (both calculated based on the corrected diffusion coefficients $D_{corrected}$) extracted from ^{19}F DOSY NMR spectra of a *Z*-**A**-enriched solution with **B** after continuous irradiation with light of a wavelength of 660 nm (refer to Table S2).

	integral range [ppm]		D [$m^2\ s^{-1}$]	$D_{corrected}$ [$m^2\ s^{-1}$]	r_s [Å]	V_s [Å^3]
Z-**A**	-116.340	-116.504	$1.034 \cdot 10^{-9}$	$1.013 \cdot 10^{-9}$	3.697	211.7
	-116.531	-116.66	$6.274 \cdot 10^{-10}$	$6.149 \cdot 10^{-10}$	6.093	947.5
Z,*Z*-**A²B²**	-118.203	-118.402	$6.260 \cdot 10^{-10}$	$6.135 \cdot 10^{-10}$	6.107	953.9
	-118.402	-118.562	$4.691 \cdot 10^{-10}$	$4.597 \cdot 10^{-10}$	8.149	2267

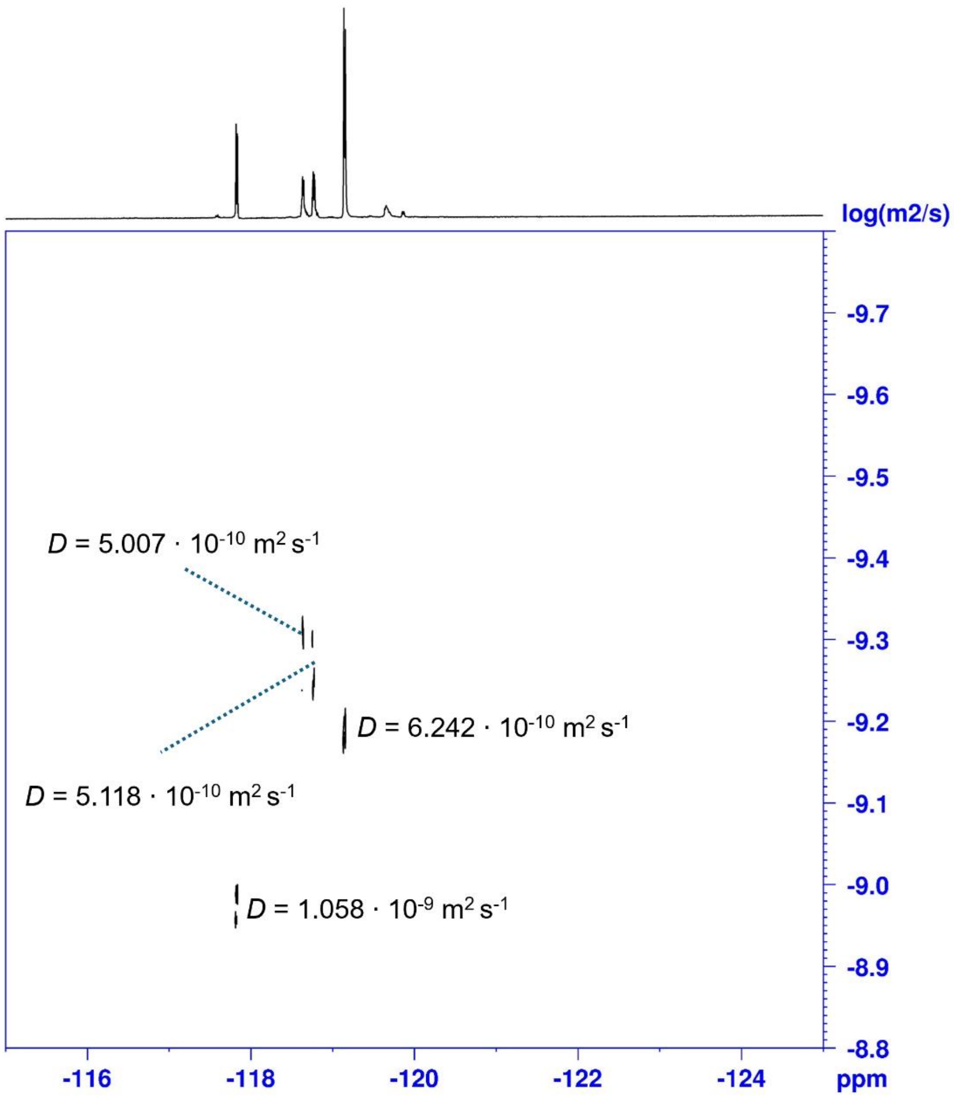

Figure S55: ^{19}F DOSY NMR spectrum ($CDCl_3$, 565 MHz) of a solution of *E*-**A** and **Pe** in the dark (refer to Table S2) with uncorrected diffusion coefficients D.

Table S8: Integral range, uncorrected and corrected diffusion coefficients D, solvodynamic radii r_s and solvodynamic volumes V_s (both calculated based on the corrected diffusion coefficients $D_{corrected}$) extracted from ^{19}F DOSY NMR spectra of a solution of *E*-**A** and **Pe** in the dark (refer to Table S2).

	integral range [ppm]		D [m^2 s^{-1}]	$D_{corrected}$ [m^2 s^{-1}]	r_s [Å]	V_s [Å^3]
E-**A**	-117.761	-117.87	1.058 · 10^{-9}	1.037 · 10^{-9}	3.613	197.6
$\mathbf{A^nPe^n}$	-118.576	-118.698	5.007 · 10^{-10}	4.907 · 10^{-10}	7.635	1864
$\mathbf{A^nPe^n}$	-118.698	-118.824	5.118 · 10^{-10}	5.016 · 10^{-10}	7.469	1746
E,*E*-$\mathbf{A^2Pe^2}$	-119.085	-119.201	6.242 · 10^{-10}	6.117 · 10^{-10}	6.124	962.2

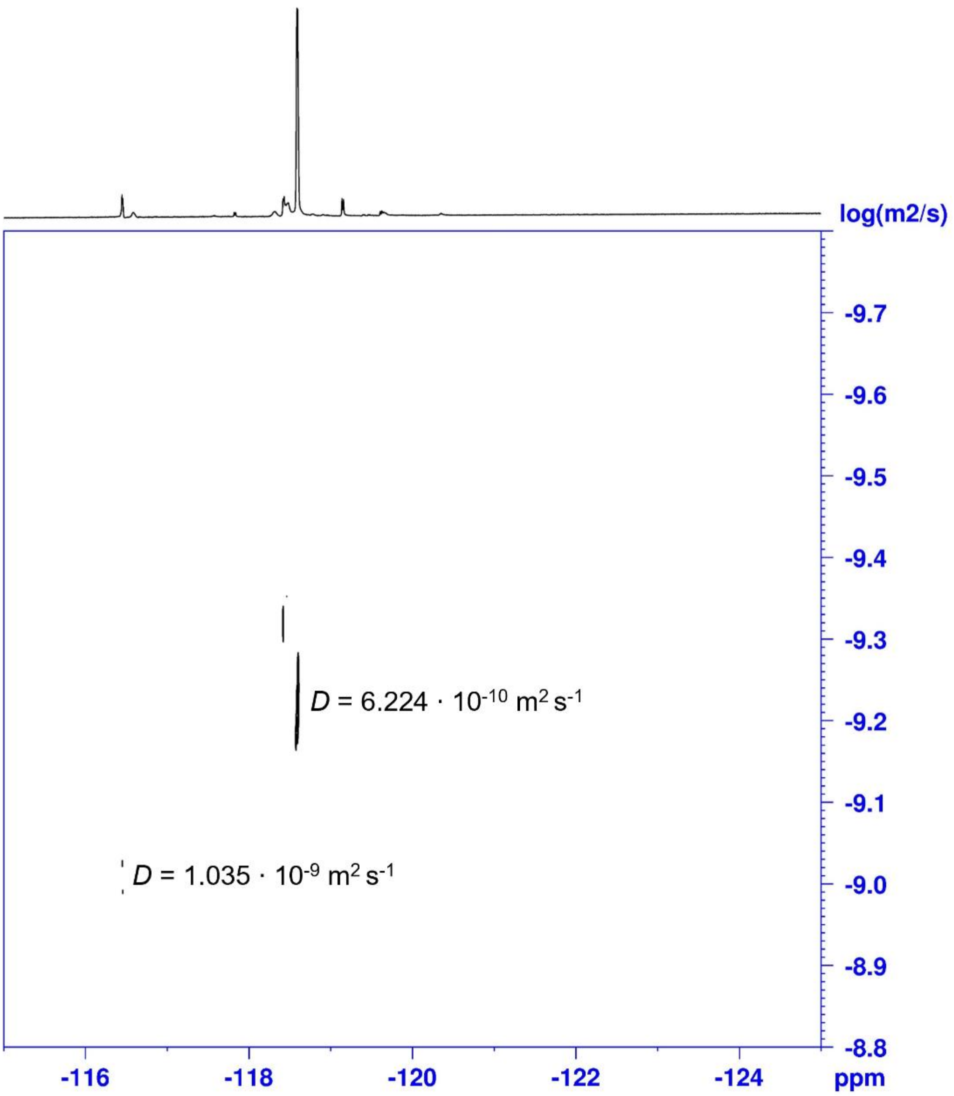

Figure S56: ^{19}F DOSY NMR spectrum ($CDCl_3$, 565 MHz) of a solution of *Z*-**A**-enriched solution with **Pe** after continuous irradiation with light of a wavelength of 660 nm (refer to Table S2) with uncorrected diffusion coefficients *D*.

Table S9: Integral range, uncorrected and corrected diffusion coefficients *D*, solvodynamic radii r_s and solvodynamic volumes V_s (both calculated based on the corrected diffusion coefficients $D_{corrected}$) extracted from ^{19}F DOSY NMR spectra of a *Z*-**A**-enriched solution with **Pe** after continuous irradiation with light of a wavelength of 660 nm (refer to Table S2).

	integral range [ppm]		D [m^2 s^{-1}]	$D_{corrected}$ [m^2 s^{-1}]	r_s [Å]	V_s [$Å^3$]
Z-**A**	-116.406	-116.486	1.035 · 10^{-9}	1.014 · 10^{-9}	3.694	211.1
Z,*Z*-$\mathbf{A^2Pe^2}$	-118.525	-118.667	6.224 · 10^{-10}	6.100 · 10^{-10}	6.142	970.6

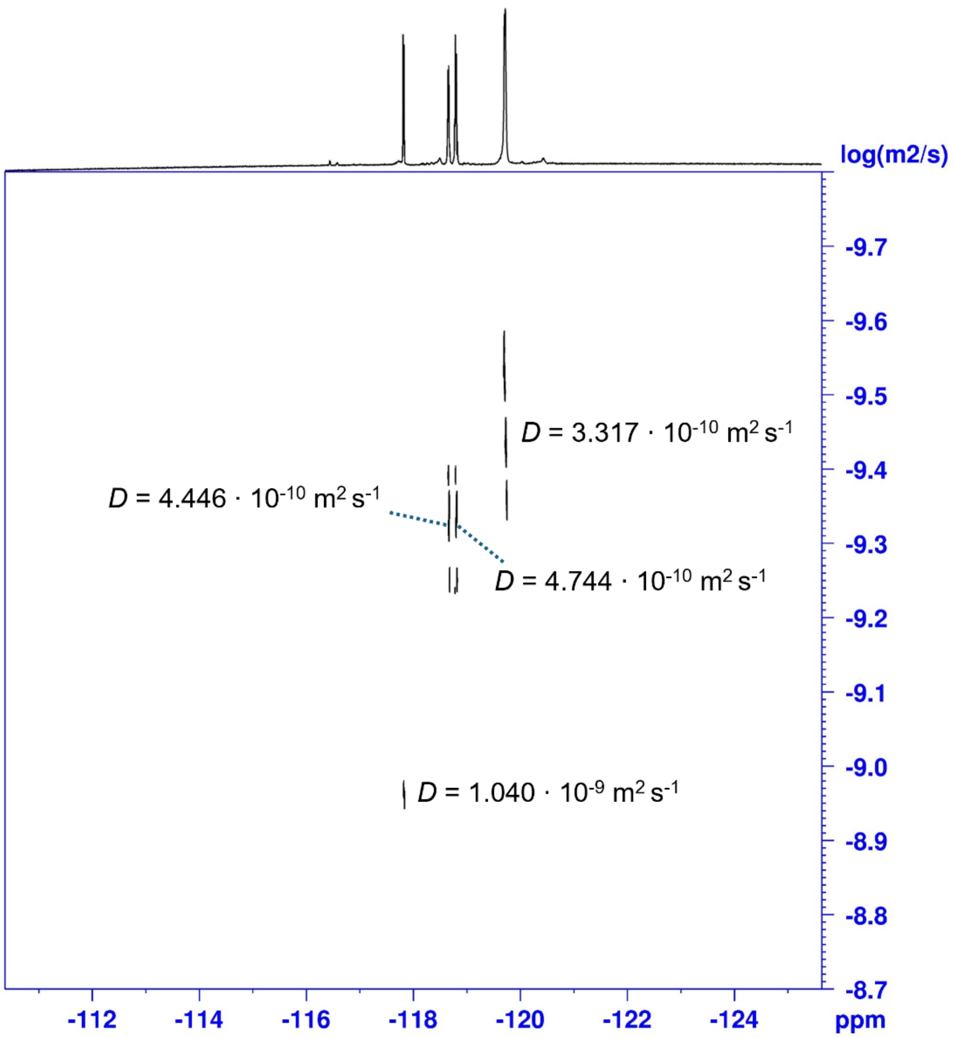

Figure S57: ^{19}F DOSY NMR spectrum ($CDCl_3$, 565 MHz) of a solution of *E*-**A** and **H** in the dark (refer to Table S2) with uncorrected diffusion coefficients *D*.

Table S10: Integral range, uncorrected and corrected diffusion coefficients *D*, solvodynamic radii r_s and solvodynamic volumes V_s (both calculated based on the corrected diffusion coefficients $D_{corrected}$) extracted from ^{19}F DOSY NMR spectra of a solution of *E*-**A** and **H** in the dark (refer to Table S2).

	integral range [ppm]		*D* [m^2 s^{-1}]	$D_{corrected}$ [m^2 s^{-1}]	r_s [Å]	V_s [Å^3]
E-**A**	-117.778	-117.86	$1.040 \cdot 10^{-9}$	$1.019 \cdot 10^{-9}$	3.676	208.0
E-$\mathbf{A^nH^n}$	-118.597	-118.707	$4.446 \cdot 10^{-10}$	$4.357 \cdot 10^{-10}$	8.598	2662.7
E-$\mathbf{A^nH^n}$	-118.734	-118.846	$4.744 \cdot 10^{-10}$	$4.649 \cdot 10^{-10}$	8.058	2192
E-$\mathbf{A^nH^{n+1}}$	-119.597	-119.792	$3.317 \cdot 10^{-10}$	$3.251 \cdot 10^{-10}$	11.525	6412

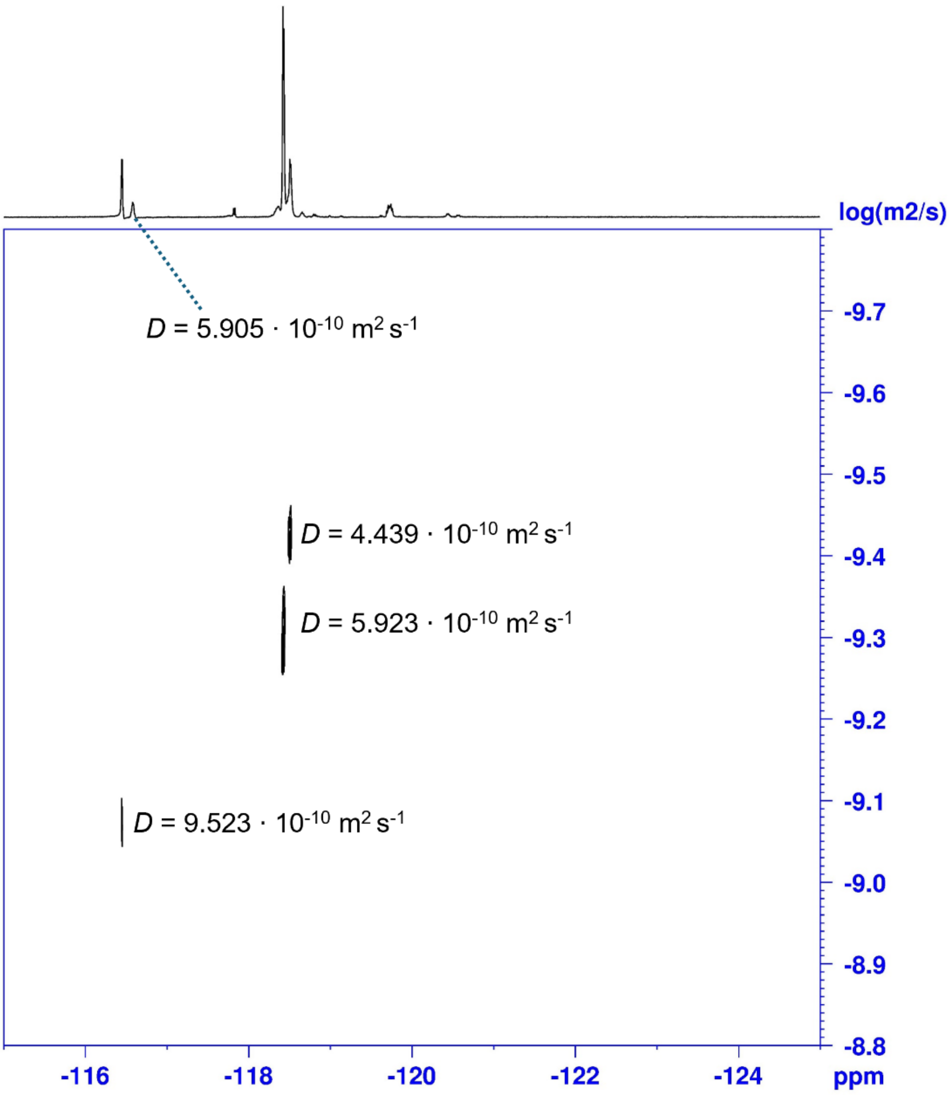

Figure S58: ^{19}F DOSY NMR spectrum ($CDCl_3$, 565 MHz) of a solution of *Z*-**A**-enriched solution with **H** after continuous irradiation with light of a wavelength of 660 nm (refer to Table S2) with uncorrected diffusion coefficients *D*.

Table S11: Integral range, uncorrected and corrected diffusion coefficients *D*, solvodynamic radii r_s and solvodynamic volumes V_s (both calculated based on the corrected diffusion coefficients $D_{corrected}$) extracted from ^{19}F DOSY NMR spectra of a *Z*-**A**-enriched solution with **H** after continuous irradiation with light of a wavelength of 660 nm (refer to Table S2).

	integral range [ppm]		D [$m^2\ s^{-1}$]	$D_{corrected}$ [$m^2\ s^{-1}$]	r_s [Å]	V_s [$Å^3$]
Z-**A**	-116.394	-116.497	$9.523 \cdot 10^{-10}$	$9.333 \cdot 10^{-10}$	4.014	271.0
	-116.497	-116.642	$6.025 \cdot 10^{-10}$	$5.905 \cdot 10^{-10}$	6.345	1070
Z,*Z*-$\mathbf{A^2H^2}$	-118.373	-118.449	$5.923 \cdot 10^{-10}$	$5.805 \cdot 10^{-10}$	6.454	1126
Z,*Z*-$\mathbf{A^2H^2}$	-118.449	-118.555	$4.439 \cdot 10^{-10}$	$4.350 \cdot 10^{-10}$	8.612	2675

VI. Crystallographic details

Single-crystals were mounted using a microfabricated polymer film crystal-mounting tool (dual-thickness MicroMount, MiTeGen) using low-viscosity oil (perfluoropolyalkylether; viscosity 1800 cSt, ABCR) to reduce X-ray absorption and scattering. The structures were solved by intrinsic phasing (SHELXT-2013) and refined by full-matrix least-squares methods on F^2 (SHELXL-2014).[9] The crystals of *E*,*E*-$\mathbf{A^2O^2}$ were grown by slow evaporation of a solution of *E*,*E*-$\mathbf{A^2O^2}$ in a solvent mixture of $CHCl_3$ and EtOH. A rectangular dark red prism was mounted, and the structure was obtained at 100 K using Cu-K_α radiation, giving the structure *E*,*E*-$\mathbf{A^2O^2}$-α (two half molecules in the asymmetric unit, CCDC 2429411). The slow vapor-liquid diffusion of Et_2O into a solution of *E*,*E*-$\mathbf{A^2O^2}$ in a solvent mixture of $CHCl_3$ and EtOH again yielded dark red rectangular prisms, and in addition, dark red platelets. Single-crystal XRD measurements of the red platelets revealed another modification, *E*,*E*-$\mathbf{A^2O^2}$-β (CCDC 2429410) with half a molecule in the asymmetric unit. Thin, needle-like crystals of *E*,*E*-$\mathbf{A^2Pr^2}$ (CCDC 2429409) where obtained by mixing a chloroform stock solution of Z-**A** with **Pr** in acetonitrile. The structure shows two B-Alerts due to the nature of the measured small platelet-shaped crystal, caused by both the poorer-quality dataset and the framework disorder of the whole macrocycle, which was not modelled. However, there is no doubt about the general structural assignment of the macrocyclic structure formed.

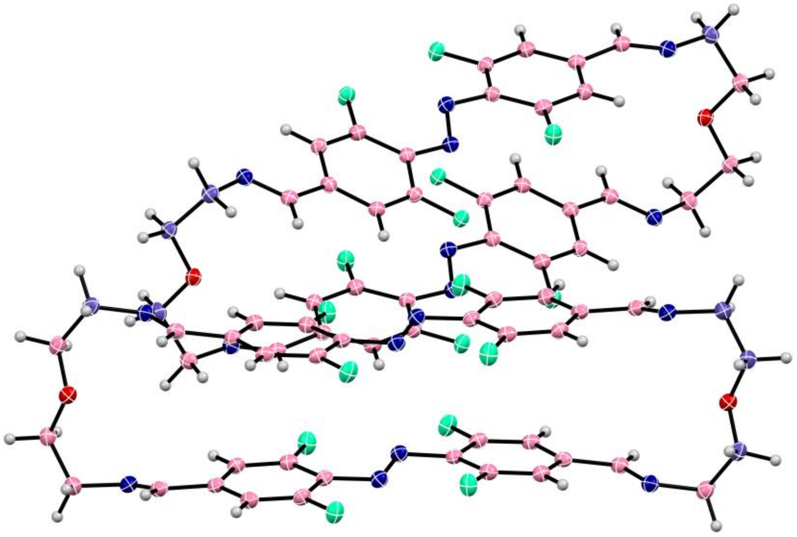

Figure S59: *E*,*E*-$\mathbf{A^2O^2}$-α with two independent macrocycles with thermal ellipsoids set at 50% probability. The structure was measured at 100 K and solved in the triclinic space group $P\overline{1}$, with R_{int} = 0.09, R_1 = 0.04, and wR_2 = 0.11.

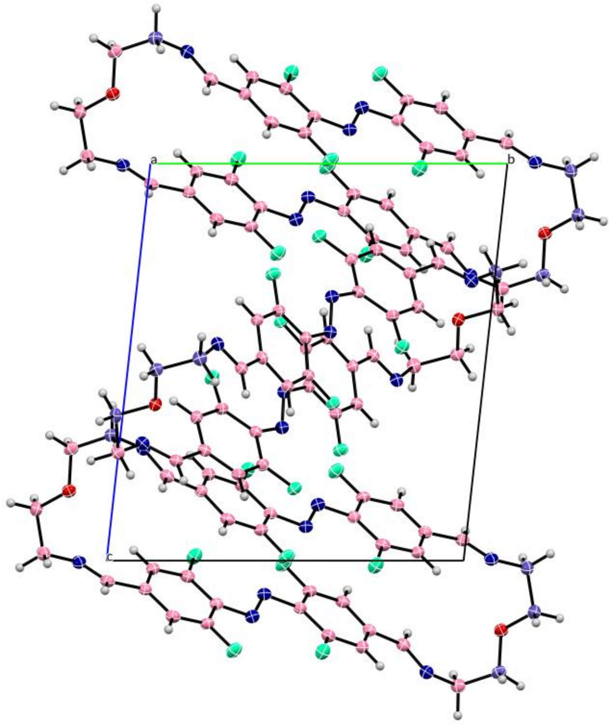

Figure S60: View of the unit cell of *E*,*E*-**A²O²**-α along the crystallographic *a* axis.

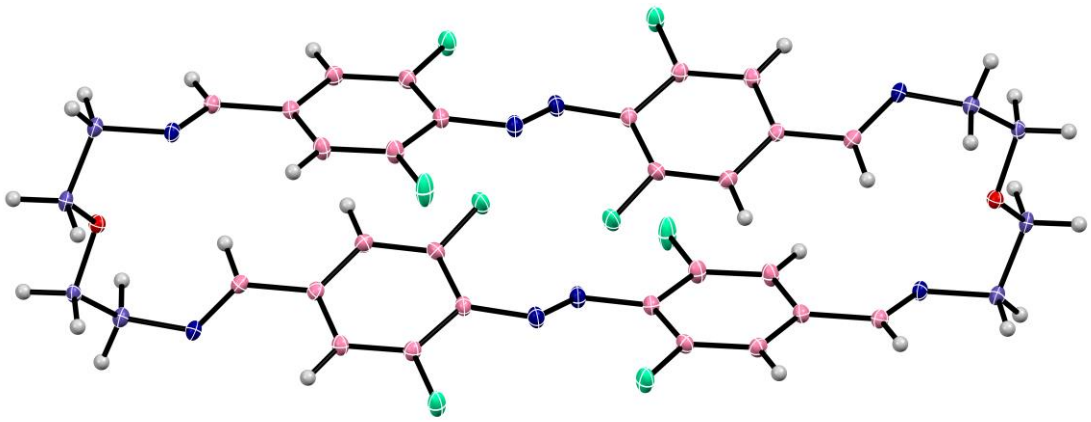

Figure S61: *E*,*E*-**A²O²**-β with one independent macrocycle in the asymmetric unit, thermal ellipsoids set at 50% probability. The structure was measured at 100 K and solved in the triclinic space group $P\overline{1}$, with R_{int} = 0.02, R_1 = 0.04, and wR_2 = 0.10.

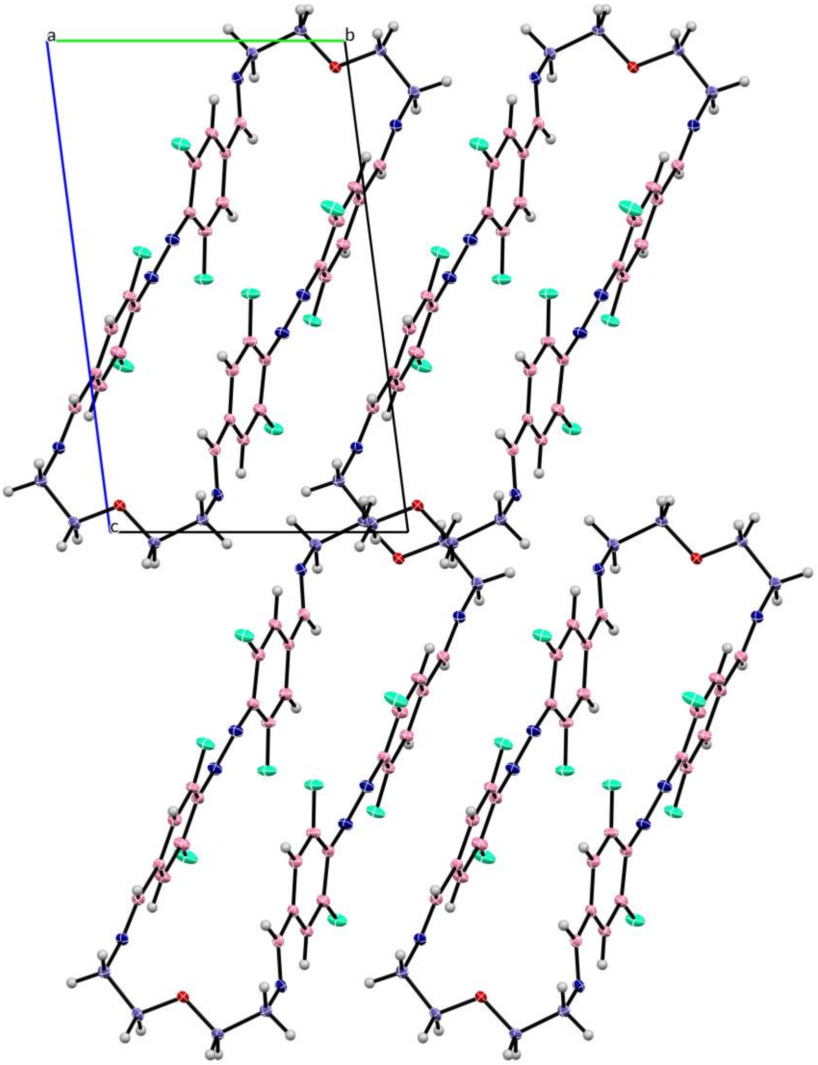

Figure S62: View of the packing of *E*,*E*-**A²O²**-β along the crystallographic *a* axis.

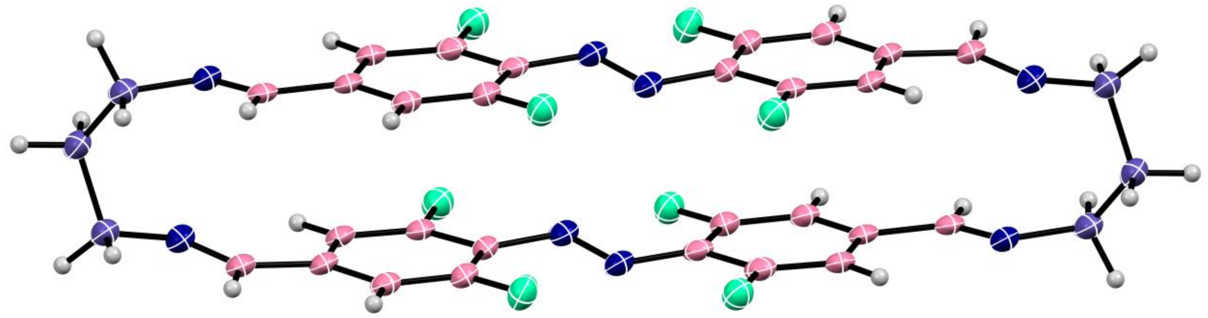

Figure S60: *E*,*E*-**A²Pr²** with half on an independent macrocycle in the asymmetric unit, one macrocycle depicted with thermal ellipsoids set at 50% probability. The structure was measured at 100 K and solved in the triclinic space group $P\overline{1}$, with R_{int} = 0.06, R_1 = 0.14, and wR_2 = 0.44 (framework disorder not modelled).

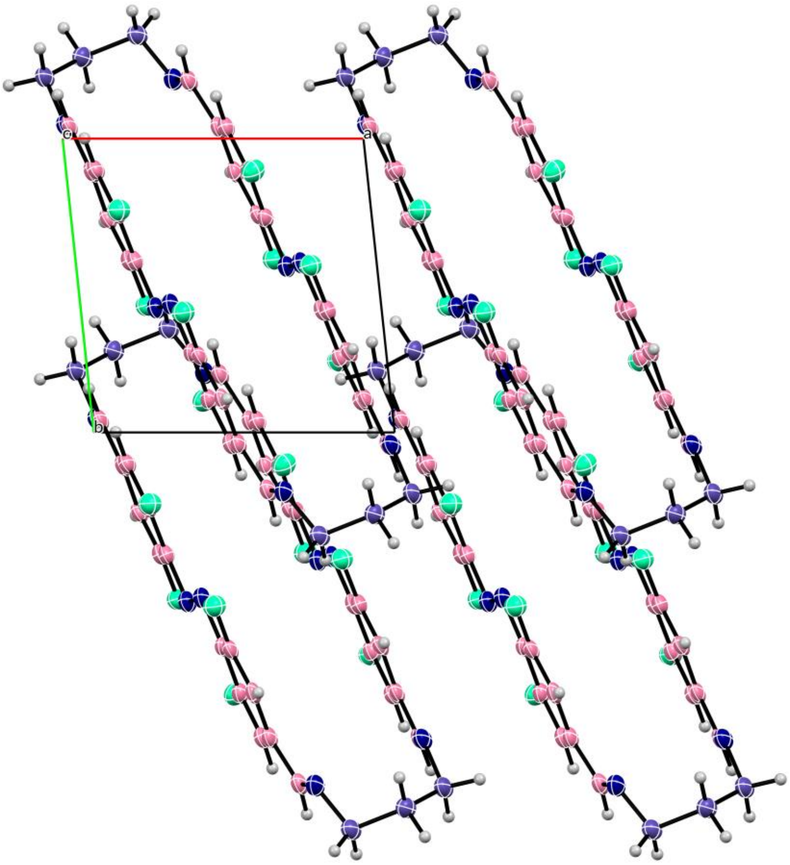

Figure S63: View of the packing of *E*,*E*-**A²Pr²** along the crystallographic *c* axis.

VII. NMR and MS Spectra

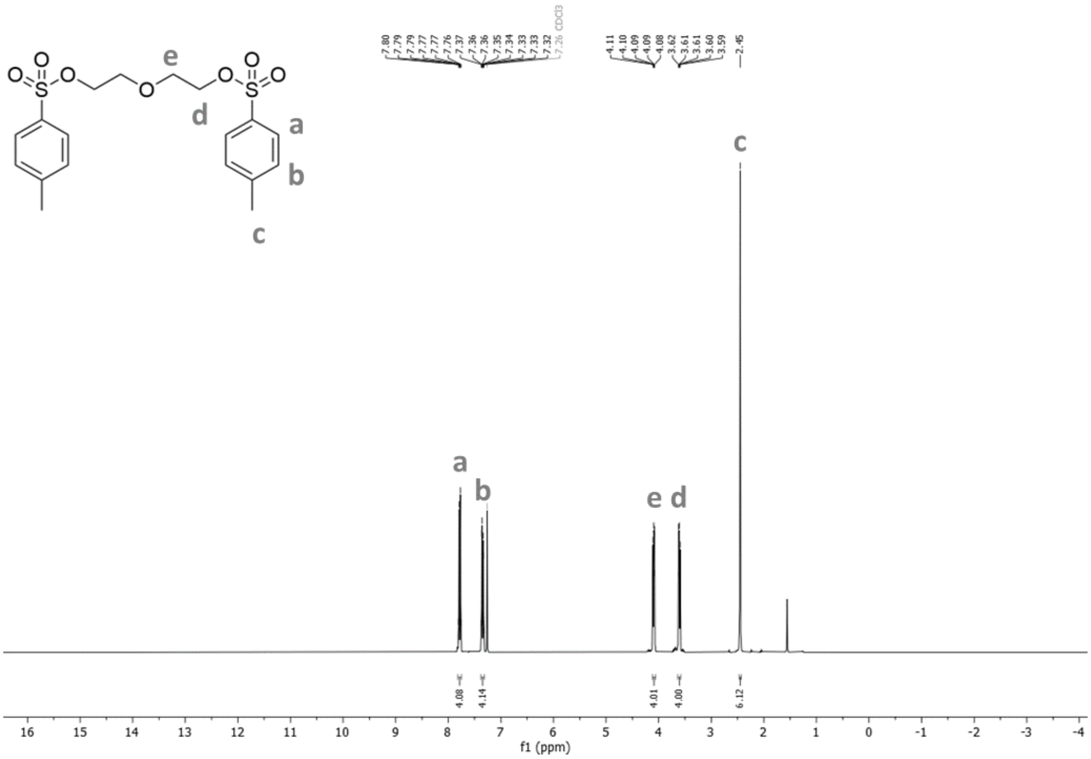

Figure S64: ^{1}H NMR spectrum of tosylate **S1** ($CDCl_3$, 300 MHz).

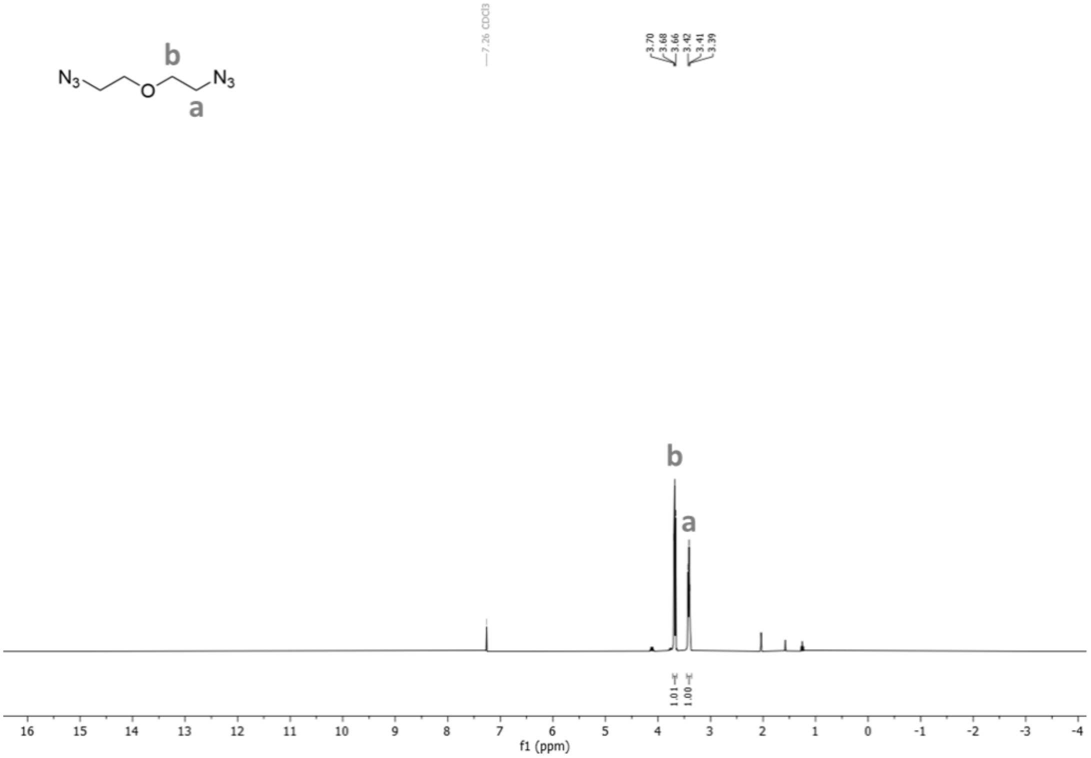

Figure S60: ^{1}H NMR spectrum of azide **S2** ($CDCl_3$, 300 MHz).

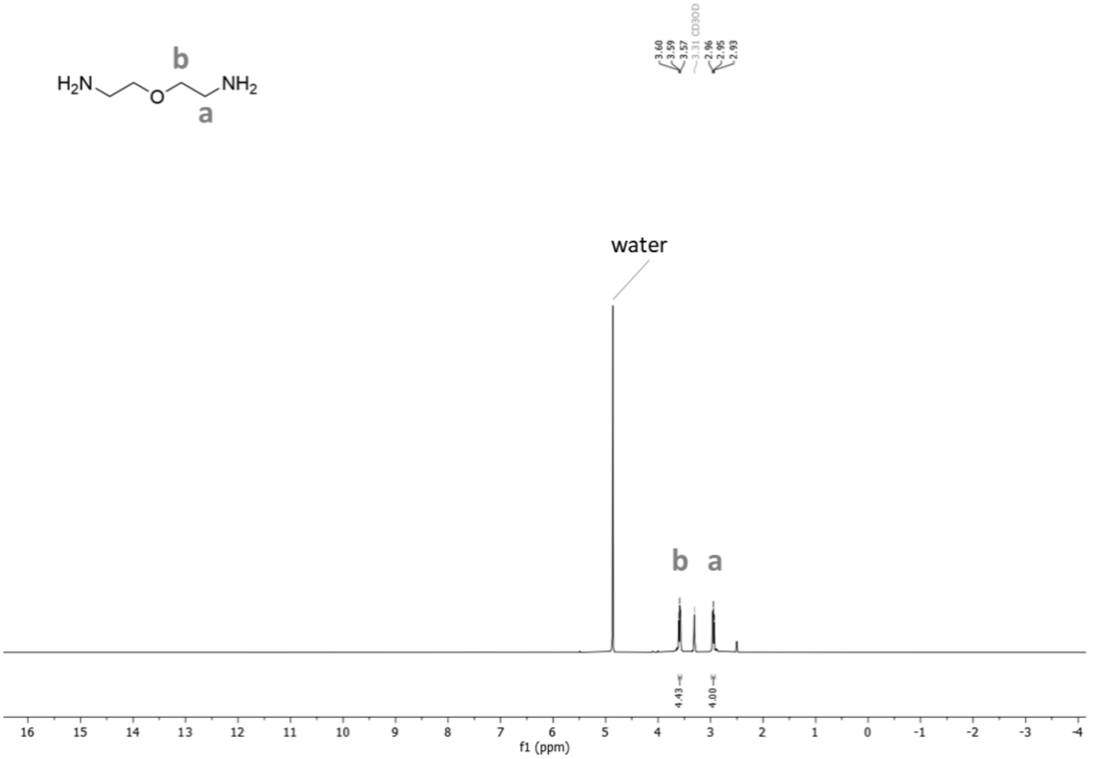

Figure S65: ^{1}H NMR spectrum of **O** ($CDCl_3$, 300 MHz).

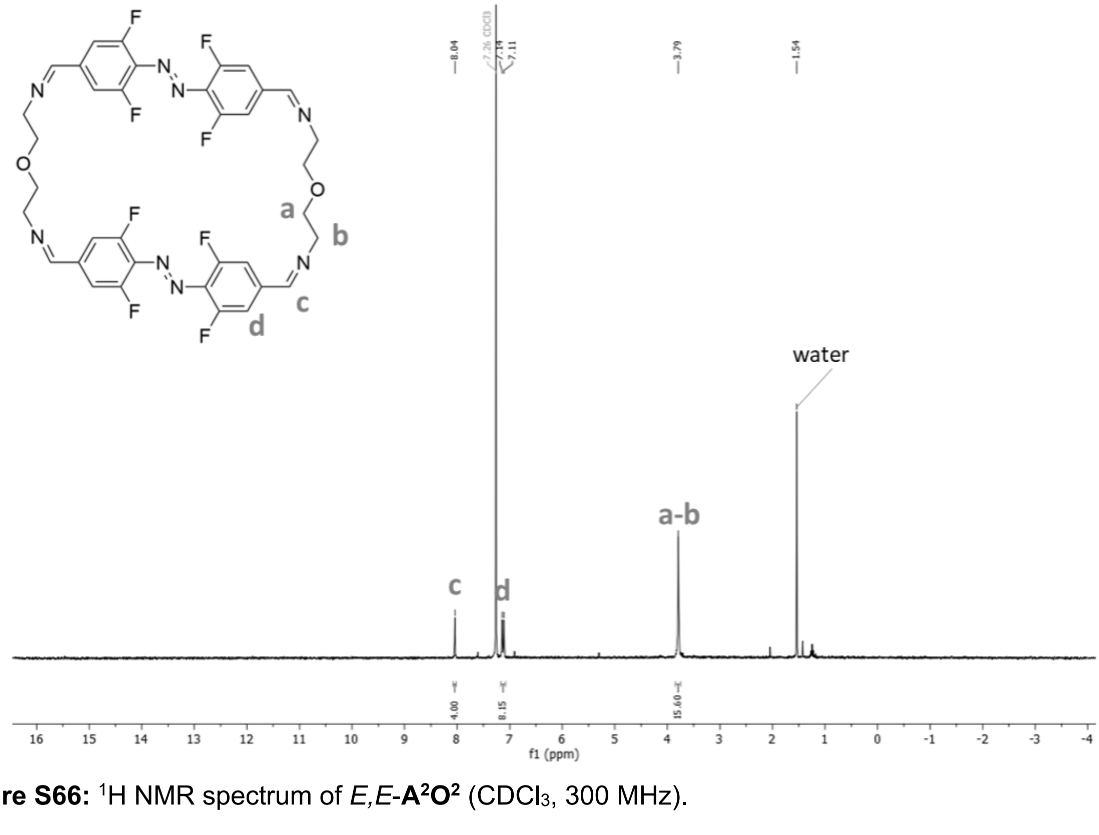

Figure S66: ^{1}H NMR spectrum of *E,E*-$\mathbf{A^2O^2}$ ($CDCl_3$, 300 MHz).

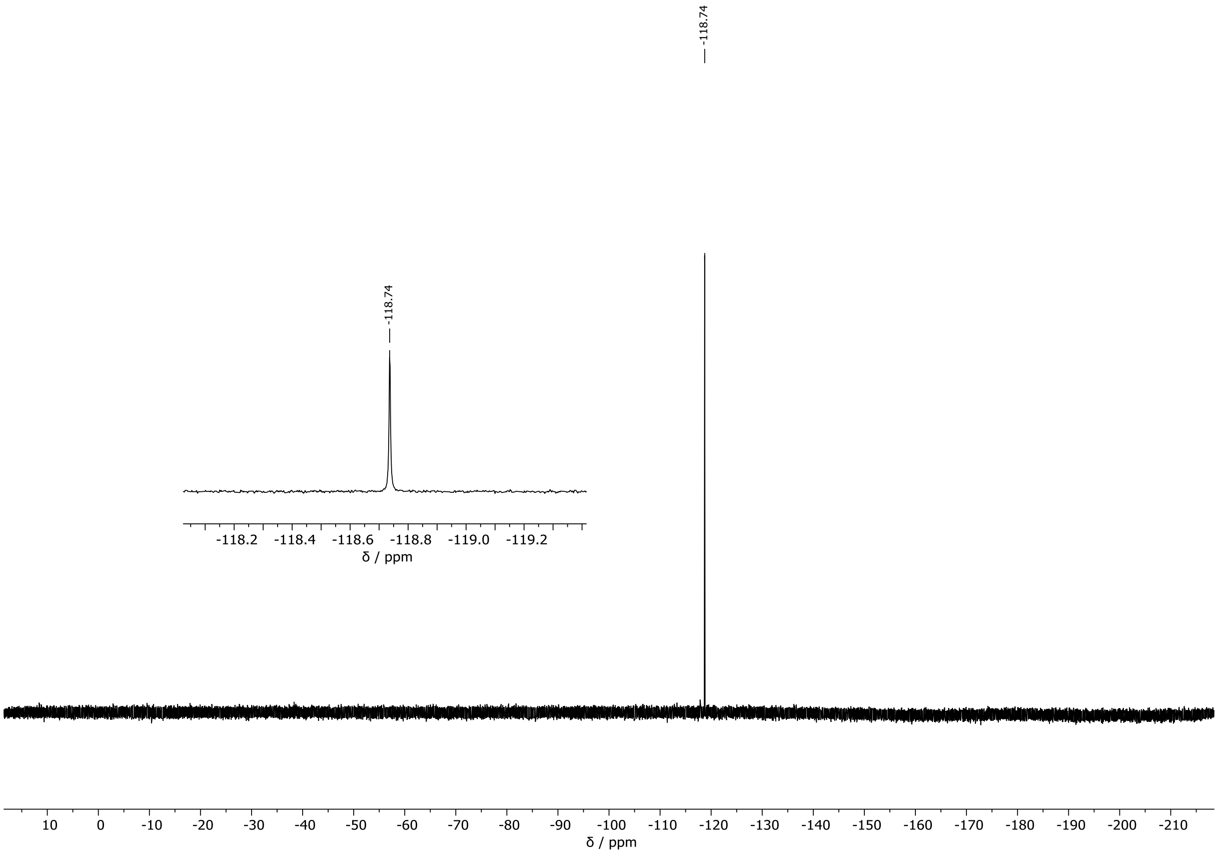

Figure S67: $^{19}F\{^{1}H\}$ NMR spectrum of *E,E*-$\mathbf{A^2O^2}$ ($CDCl_3$, 282 MHz).

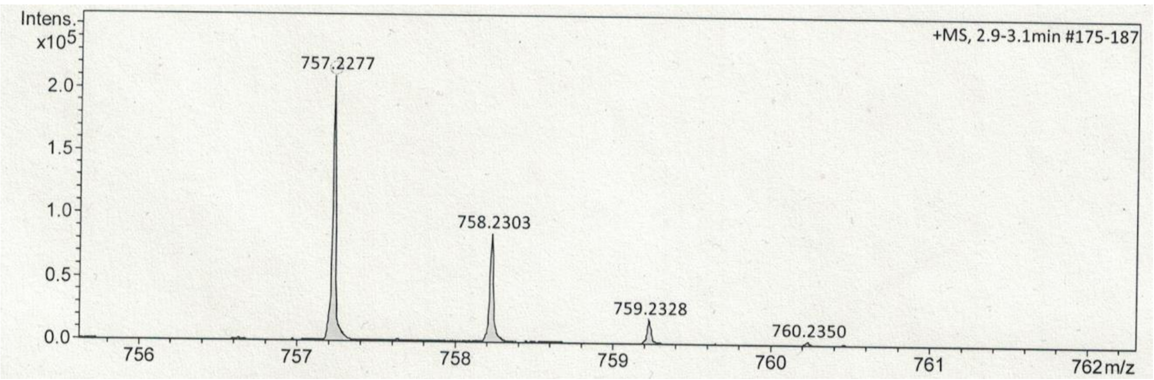

Figure S68: High resolution ESI-mass spectrum of *E,E*-$\mathbf{A^2O^2}$.

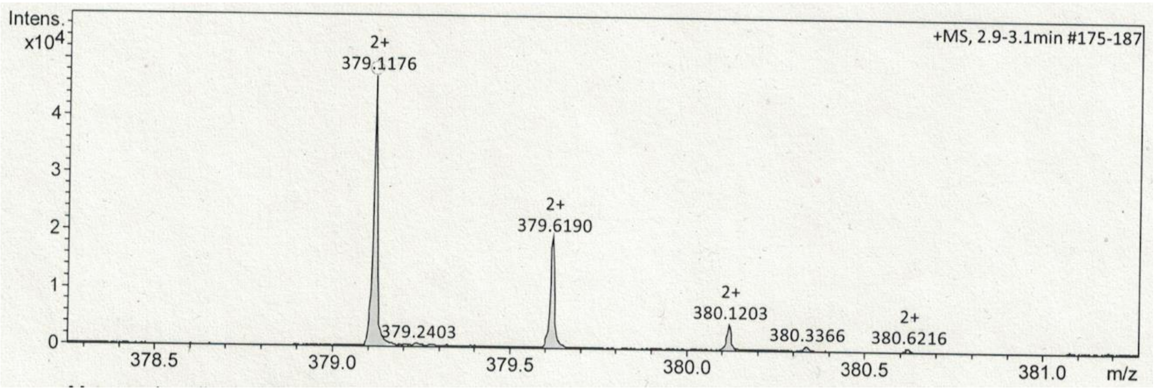

Figure S69: High resolution ESI-mass spectrum of *E,E*-$\mathbf{A^2O^2}$.

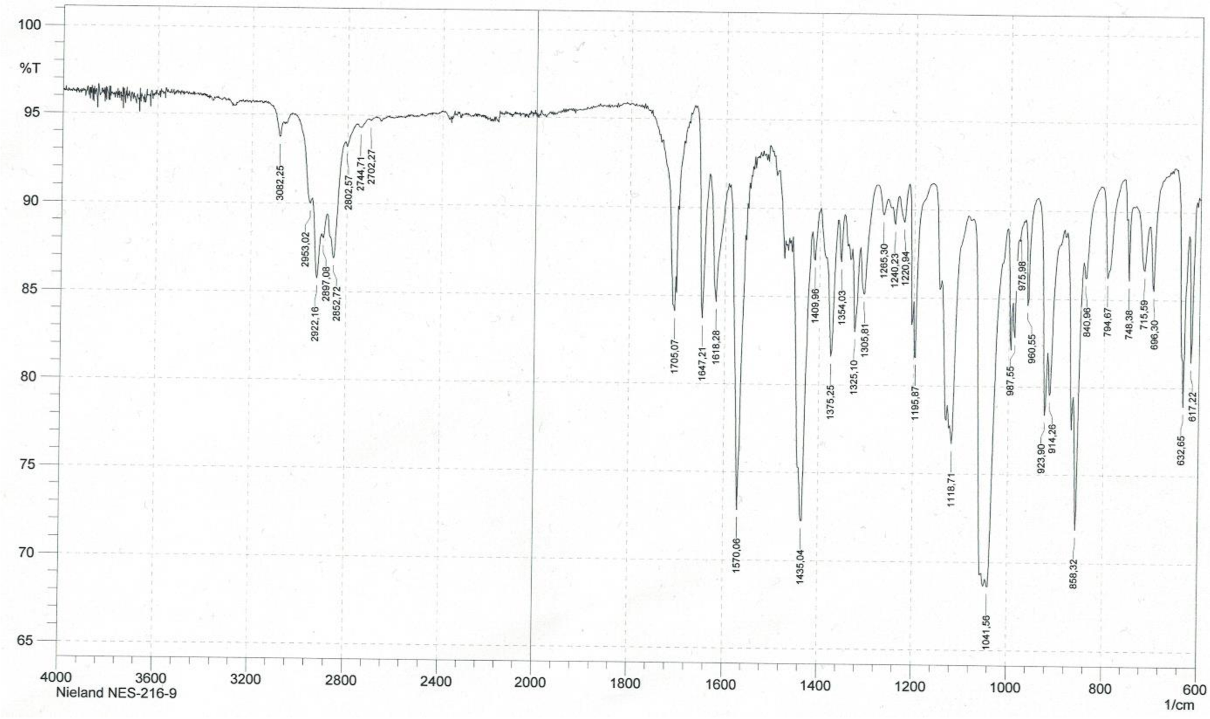

Figure S70: IR spectrum of *E,E*-$\mathbf{A^2O^2}$.

References

[1] G. R. Fulmer, A. J. M. Miller, N. H. Sherden, H. E. Gottlieb, A. Nudelman, B. M. Stoltz, J. E. Bercaw, K. I. Goldberg, *Organometallics* **2010**, *29*, 2176–2179.

[2] A.-L. Leistner, S. Kirchner, J. Karcher, T. Bantle, M. L. Schulte, P. Gödtel, C. Fengler, Z. L. Pianowski, *Chem. Eur. J.* **2021**, *27*, 8094.

[3] E. Nieland, J. Voss, A. Mix, B. M. Schmidt, *Angew. Chem. Int. Ed.* **2022**, *61*, e202212745.

[4] K. Chiba, M. Asanuma, M. Ishikawa, Y. Hashimoto, K. Dodo, M. Sodeoka, T. Yamaguchi, *Chem. Commun.* **2017**, *53*, 8751–8754.

[5] R. Mills, *J. Phys. Chem.* **1973**, *77*, 685–688.

[6] W. S. Price, H. Ide, Y. Arata, *J. Phys. Chem. A* **1999**, *103*, 448–450.

[7] D. R. Lide (Ed.) CRC Handbook of Chemistry and Physics, Internet Version. Physical Constants of Organic Compounds, CRC Press, Boca Raton, Florida, **2005**.

[8] R. A. Clará, A. C. Gómez Marigliano, H. N. Sólimo, *J. Chem. Eng. Data* **2006**, *51*, 1473–1478.
